# Comparative safety and tolerability of ketamine and esketamine for major depressive disorder: a systematic review and meta-analysis

**DOI:** 10.3389/fphar.2025.1681060

**Published:** 2025-10-29

**Authors:** Haoning Guo, Liling Tang, Miaoquan He, Wencheng Tang, Jing Liu, Silin Wu, Shuying Yuan, Jisheng Wang, Xueli Tang

**Affiliations:** ^1^ Division of Psychopharmacology, Department of Pharmacy, The Third Hospital of Mianyang, Sichuan Mental Health Center, Mianyang, China; ^2^ College of pharmacy, Chongqing Medical University, Chongqing, China; ^3^ Department of Pathology, The Third Hospital of Mianyang, Sichuan Mental Health Center, Mianyang, China; ^4^ Department of Scientific Research Management, The Third Hospital of Mianyang, Sichuan Mental Health Center, Mianyang, China

**Keywords:** ketamine, esketamine, major depressive disorder, unipolar depression, safety, meta-analysis

## Abstract

**Background:**

Ketamine and esketamine have demonstrated rapid, short-term antidepressant effects in major depressive disorder (MDD), but their relative safety remains unclear. This review aims to update the evidence on the safety of two agents for MDD and indirectly compare their safety and tolerability.

**Method:**

We systematically searched PubMed, PsycINFO, Embase, and Cochrane databases up to 1 May 2025. Eligible studies compared ketamine or esketamine with placebo, active psychotropic agents, or electroconvulsive therapy in adults with MDD.

**Results:**

We retrieved 5,473 articles, 47 of which met the inclusion criteria. For ketamine versus placebo, both dropout and incidence rates of adverse events (AEs) were statistically significant, with number needed to harm (NNH) values of 12 and 2, respectively. A similar pattern of effect sizes was found for esketamine, but with higher corresponding NNH values. Conversely, neither the meta-analysis nor NNH analyses of the incidence of serious AEs for ketamine and esketamine were statistically significant. A series of AEs like dizziness, dissociation, nausea, vertigo, and vision blurred, with relatively low NNH values, would be more likely to occur in clinical practice and exhibit dose-dependent effects. Moreover, ketamine or esketamine was associated with transient and significant psychiatric side-effects, blood pressure increases, and sedation post-dose. No significant abnormalities were observed in cognitive impairments, laboratory results, bladder symptoms, nasal examination, or addiction-related evaluations for either drug.

**Conclusion:**

Although further promising evidence supports the safety of ketamine and esketamine for MDD, the findings of this study highlight a potential tolerability advantage with esketamine over ketamine for short-term use for MDD. These findings require further validation through direct head-to-head clinical trials comparing these two drugs.

**Systematic Review registration:**

https://www.crd.york.ac.uk/PROSPERO/view/CRD42023389486.

## 1 Introduction

Major depressive disorder (MDD), commonly referred to as “unipolar depression”, affects about 350 million individuals, making it a leading global health challenge associated with severe disability, poor quality of life, and significant economic burden (American Psychiatric Association, 2024; WHO, 2016; Rapaport et al., 2005; Stecher et al., 2024). Although monoamine potentiating antidepressants have been the cornerstone of pharmacological treatment for decades, their efficacy is limited, with approximately half of patients responding inadequately, particularly in treatment-resistant depression (TRD) (Malhi and Byrow, 2016; Undurraga and Baldessarini, 2012). Moreover, these agents typically require 2–4 weeks to exert therapeutic effects (Quitkin et al., 1987; Quitkin et al., 1984), highlighting the need for more effective and faster-acting alternatives.

Growing evidence implicates that glutamatergic mechanisms and related agents may be involved in the pathophysiology and antidepressant response in mood disorders ( Gerhard et al., 2016; Lv et al.,2023; Wilkinson and Sanacora, 2019). Ketamine, a non-competitive N-methyl-D-aspartate (NMDA) receptor antagonist long used as an anesthetic, has shown rapid and potent antidepressant effects at subanesthetic doses, typically within hours (Berman et al., 2000; Kishimoto et al., 2016; Sos et al., 2013; Zarate et al., 2006). In addition, repeated intravenous injection of ketamine helps maintain short-term antidepressant efficacy (Bahji et al., 2021). However, concerns remain regarding adverse events (AEs), particularly marked dissociative states and blood pressure elevations following acute administration. Furthermore, given that intravenous ketamine is resource-intensive and relatively inconvenient in routine clinical settings, it potentially limits its broader application in depression (Bahji et al., 2021; Dean et al., 2021). Researchers have been searching for alternative formulations and delivery methods. Esketamine, the S-enantiomer of racemic ketamine with ∼4-fold greater NMDA-receptor binding affinity than R-ketamine (arketamine), makes it possible to administer lower doses of ketamine in clinical practice and reduce the dose-dependent dissociative properties of ketamine (Correia-Melo et al., 2018; Yang et al., 2019). Recently, esketamine has been developed as an intranasal formulation and approved for adults with MDD or TRD, following the completion of both acute and maintenance treatment trials (Gastaldon et al., 2021; Guo et al., 2022).

Generally, ketamine and esketamine are considered well-tolerated and relatively safe for short-term treatment of depression. Although treatment-emergent AEs, such as dissociation, anxiety, dizziness, headache, nausea, hypertension, tachycardia, and blurred vision, are frequently reported, the incidence of serious AEs and treatment discontinuation due to AEs remains relatively low (Ceban et al., 2021; McIntyre et al., 2021; Short et al., 2018). However, long-term, repeated, or high-dose ketamine use in other populations (e.g., chronic pain, anesthesia, recreational) has raised safety concerns (WHO, 2016). Previous studies have highlighted genitourinary symptoms as one of the well-documented side-effects of ketamine, including cystitis, bladder dysfunction, urinary urgency and frequency, dysuria, and occasional painful hematuria. Potential harms also include renal, hepatic, and biliary damage, neurocognitive deficits, and addiction (Katalinic et al., 2013; Morgan and Curran, 2012). Therefore, the acute and long-term safety profile of both drugs in depressed patients warrants further in-depth exploration and systematic evaluation. Since several new randomized controlled trials (RCTs) have been published recently, our primary aim is to update the evidence on the safety of ketamine and esketamine in MDD.

Moreover, previous studies have suggested that intravenous ketamine has comparable efficacy to intranasal esketamine regarding remission/response rates and depression assessment scale scores changes, while ketamine may act faster (Calder et al., 2024; Singh et al., 2023). However, their comparative safety has not been established due to a lack of head-to-head comparisons and methodological heterogeneity in clinical trials. Number needed to harm (NNH) refers to the average number of patients needed to be exposed to a treatment to experience undesirable events (Calder et al., 2024). NNH value is not necessarily an effect size per se, but its pragmatism has been identified for translating the pooled results of clinical trials into clinically relevant metrics that allow for indirect comparisons of the relative safety and effectiveness across treatments (Calder et al., 2024; Wong et al., 2024). Herein, the secondary aim of this review is to indirectly compare the relative tolerability of ketamine and esketamine for MDD when compared to the same control (i.e., placebo) using the NNH metric and pooled results from standard meta-analyses.

## 2 Materials and methods

In this study, we followed the preferred reporting items for systematic reviews and meta-analysis (PRISMA) reporting guidelines (Page et al., 2021). The protocol of this review has been registered with PROSPERO (registration number CRD42023389486).

### 2.1 Literature search

We systematically searched the Cochrane Central Register of Controlled Trials, PubMed, PsycINFO, and Embase for articles from the inception of each database to 1 May 2025 (Supplementary Appendix 2). Reference lists of retrieved articles and international trial registries (ClinicalTrials.gov) were also checked for other eligible studies. No restrictions on language or publication status were applied in the search. After the first selection based on title or abstract by two authors, the full text of all potentially eligible studies was screened independently on inclusion and exclusion criteria by two authors. Any discrepancy in judgment was resolved through discussion or, if necessary, by consulting a third author.

### 2.2 Selection criteria

#### 2.2.1 Types of studies

We included only RCTs (either crossover or parallel) comparing ketamine or esketamine to a comparator intervention in participants with unipolar depression. For studies with cross-over designs, we only considered results from the first period before cross-over. Exclusion criteria included: studies where the full-text or abstract was not available; duplicated publications; and studies that included ECT as a concomitant treatment.

#### 2.2.2 Types of participants

Eligible trials included participants of aged 18 years or older with a primary diagnosis of MDD or TRD according to the standard operational criteria, regardless of gender.

#### 2.2.3 Types of interventions

Experimental interventions included ketamine and esketamine, with no restrictions on the administration route, dose, or frequency. Comparator interventions included placebo (pill or saline infusion), any pharmacologically active agent, or ECT. All interventions could be used as monotherapy or in combination with other treatment regimens.

#### 2.2.4 Types of outcome measures

Safety and tolerability were primarily assessed through reported AEs and dropouts due to AEs. Secondary outcomes comprised supplementary safety data obtained from multiple structured assessment scales or questionnaires. AEs were coded with the preferred terms (PTs) through the Medical Dictionary for Regulatory Activities (version 25.0) and then categorized by system organ class (SOC) (Dean et al., 2021).

### 2.3 Data extraction

Two authors independently extracted study characteristics and outcome data from included studies, including bibliographic details (first author, publication year, country), participant characteristics (baseline sample sizes, age, sex, comorbidity, depression diagnosis, depression severity), intervention details (dosage range, frequency, administration route, duration), and outcome measures of interest from the included studies. Any disagreements were resolved by consensus or by consulting a third author. Two authors transferred the data into the Review Manager 5.3 (RevMan 5.3) file and double-checked that the data were entered correctly.

### 2.4 Risk of bias (quality) assessment

Two reviewers independently completed the quality assessment of all included studies using the Cochrane risk of bias tool (Higgins et al., 2024). We assessed the potential bias in random sequence generation, allocation concealment, blinding of participants and personnel, blinding of outcome assessment, incomplete outcome data, selective reporting, and other bias. Each item was judged either as high, low, or unclear risk of bias.

### 2.5 Statistical analysis

All data analyses were performed using RevMan 5.3 and STATA 16.0 in this review. When measuring effect size, risk ratio (RR) with a corresponding 95% confidence interval (CI) was calculated for dichotomous data, and mean difference (MD) or standardized mean difference (SMD) with a corresponding 95% CI for continuous data, respectively. We employed the MD when measuring an outcome using the same scale, and the SMD where different scales were applied to measure the same results (Dean et al., 2021). When a study contained over two treatment groups, we included all related treatment groups in the comparisons. If the data were dichotomous, we combined them into one group. If the data were continuous, we pooled the data using the formula in the Cochrane Handbook for Systematic Reviews of Interventions, Section 6.5.2 (Higgins et al., 2024). We performed meta-analyses only if the clinical data were sufficient and the underlying treatments were similar enough; otherwise, we provided a narrative description of the results. We calculated the absolute risk increase (ARI) for the primary safety outcomes occurring at any time during the treatment period, and the corresponding NNH as 1/ARI when the 95% CI for ARI excluded the null (Citrome et al., 2020; Feldman et al., 2024). As a rule of thumb, a lower NNH value for adverse outcomes indicates that the treatment would be less tolerable (Citrome et al., 2020). Given the potential heterogeneity in the clinical trials, we applied random effect models for all meta-analyses (Dean et al., 2021). Heterogeneity among studies was assessed by calculating the *I* ^*2*^ statistic. An *I* ^*2*^ value of 0%–40% may not be important, 30%–60% may represent moderate heterogeneity, 50%–90% substantial heterogeneity, and 75%–100% considerable heterogeneity (Higgins et al., 2024). If the direction and size of the treatment effects suggested important heterogeneity, we undertook sensitivity analyses by excluding each study sequentially and re-running the meta-analyses, when a minimum of five studies were included (Higgins et al., 2024; Rutigliano et al., 2016). To further evaluate the robustness of the primary outcomes, we planned other sensitivity analyses by excluding studies with crossover, double-randomization, or open-label design. Additionally, we planned to conduct subgroup analyses, if possible, for several variables, such as comparator interventions, dosage, and frequency of dosing. We further conducted the funnel plot and Egger’s test to investigate the possibility of publication bias, when more than 10 studies were included in the meta-analysis (Joober et al., 2012). Finally, the grading of recommendations, assessment, development, and evaluations (GRADE) assessments of the certainty of evidence for the primary outcomes were conducted using GRADEprofiler 3.6. We considered a *p* < 0.05 (two-tailed) and a 95% CI not crossing the no-effect line to be statistically significant.

## 3 Results

### 3.1 Search results

**FIGURE 1:**
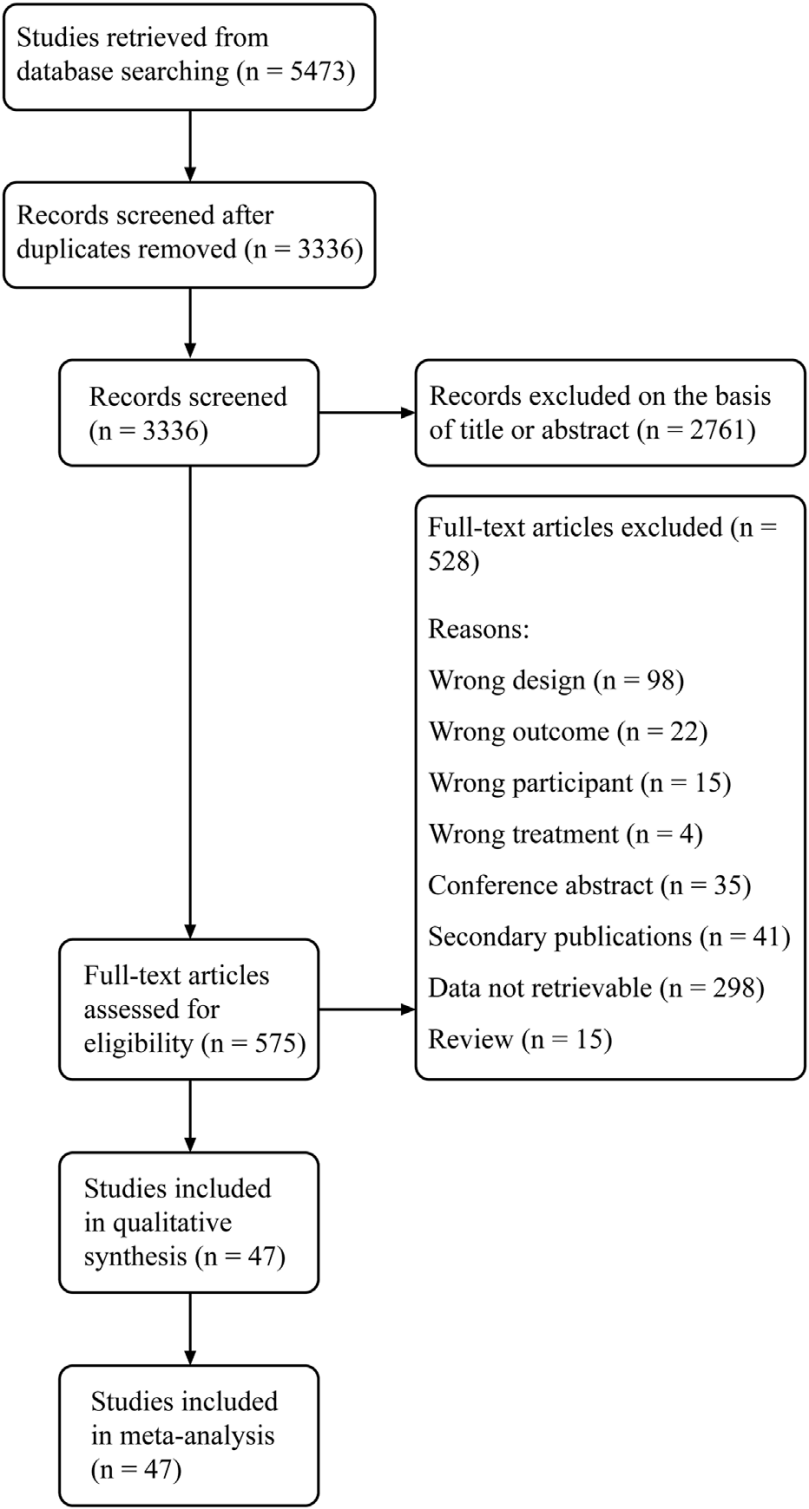
PRISMA study flow diagram. PRISMA, preferred reporting items for systematic reviews and meta-analysis.

The study flow diagram of literature search and study selection is shown in Figure 1. From a total of 5,473 articles retrieved from electronic and manual searches, we removed 2,137 duplications and further excluded 2,761 records on the basis of the title and abstract. After the full-text screening of the remaining 575 records, 47 eligible studies were eventually included in this review.

### 3.2 Characteristics of included studies

Of these 47 studies, 33 trials evaluated the safety of ketamine (Arabzadeh et al., 2018; Berman et al., 2000; Colla et al., 2024; Domany et al., 2019; Downey et al., 2016; Ekstrand et al., 2022; Fava et al., 2020; Gálvez et al., 2018; Ghasemi et al., 2014; Glue et al., 2024; Grunebaum et al., 2018; Hu et al., 2016; Ionescu et al., 2019; Keilp et al., 2021; Kheirabadi et al., 2020; Lapidus et al., 2014; Li et al., 2016; Lijffijt et al., 2022; Loo et al., 2023; Murrough et al., 2015; Murrough et al., 2013; Ohtani et al., 2024; Pattanaseri et al., 2024; Price et al., 2014; Seraj et al., 2025; Shiroma et al., 2020; Singh et al., 2016b; Sos et al., 2013; Su et al., 2023; Su et al., 2017; Tiger et al., 2020; Zarate et al., 2006; Zolghadriha et al., 2024), and the remaining evaluated esketamine (Canuso et al., 2018; Chen et al., 2023; Daly et al., 2018; Daly et al., 2019; Fedgchin et al., 2019; Fu et al., 2020; Hong et al., 2025; Ionescu et al., 2021; Ochs-Ross et al., 2020; Popova et al., 2019; Reif et al., 2023; Singh et al., 2016a; Smith-Apeldoorn et al., 2024; Takahashi et al., 2021). The total number of participants enrolled in these studies was 5,046, with sample sizes ranging from five to 676. Only one trial focused on recruiting elderly adults over the age of 65. In all included relevant studies, esketamine was compared with placebo and quetiapine in 13 and one studies, respectively. Similarly, ketamine was also compared with different controls, including placebo in 17 trials, midazolam in 13, and ECT in three. In most studies, ketamine was administered intravenously; among the remaining nine studies, six administered by oral route, two by nasally, and one by subcutaneously. Intranasal administration of esketamine was employed in all but two studes. The detailed clinical characteristics and outcome measures of the included studies are presented in Supplementary Tables S1 and S2, respectively.

### 3.3 Risk of bias assessment

**FIGURE 2:**
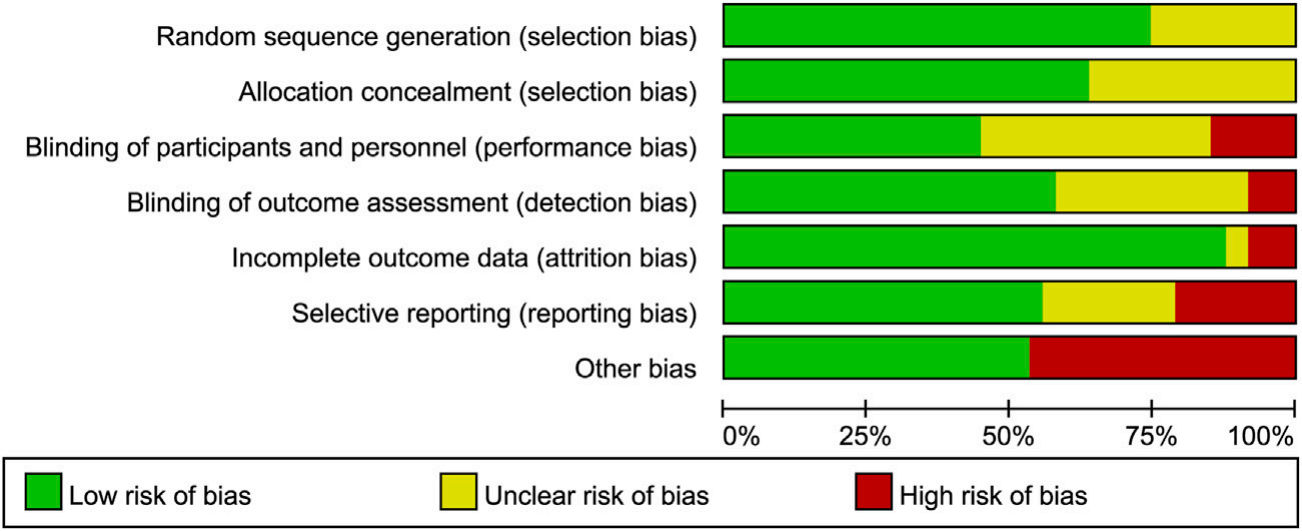
Risk of bias graph. The authors’ judgements for each risk of bias item are presented as a percentage of all included studies.

**FIGURE 3:**
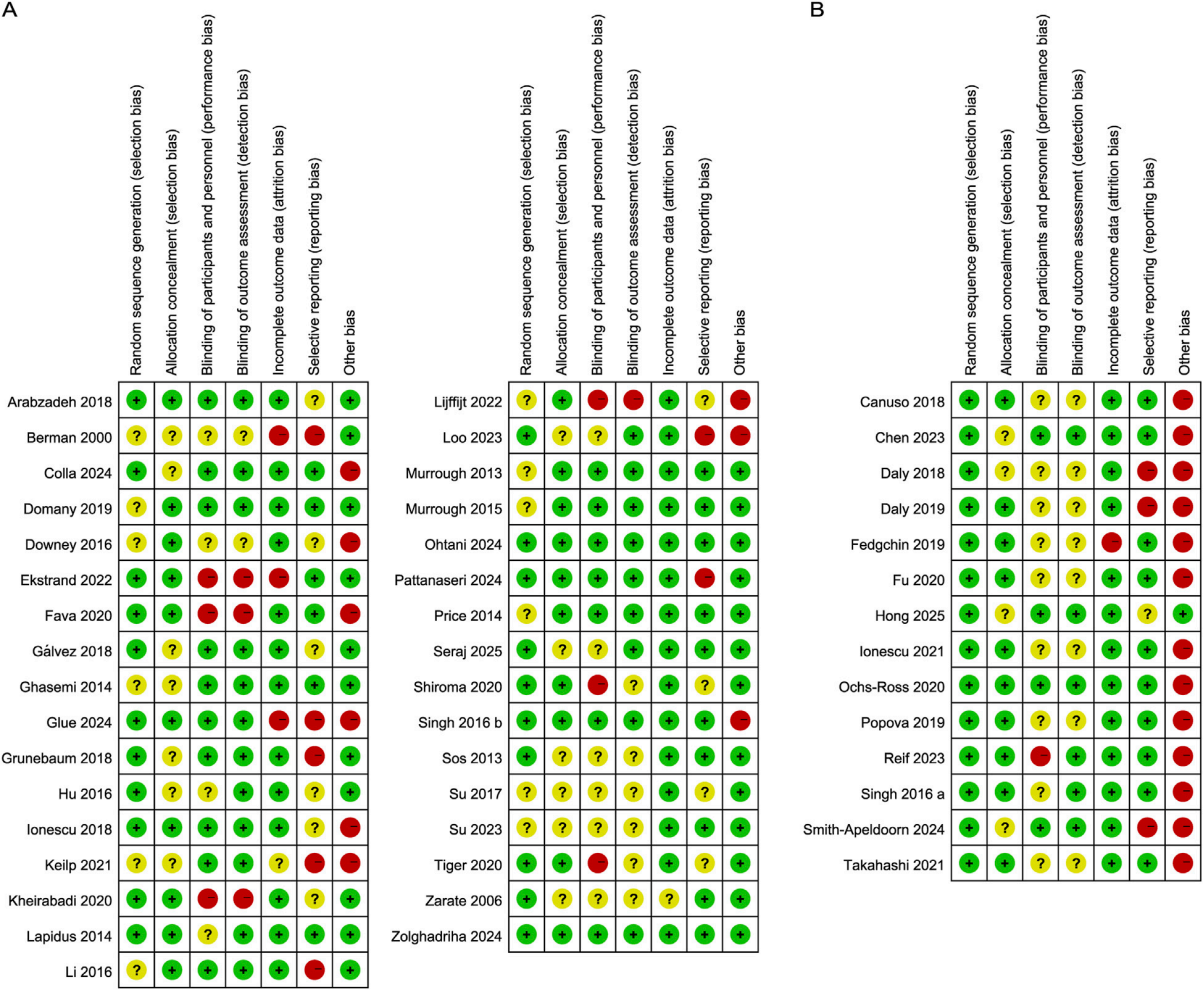
Risk of bias summary. The authors’ judgements on the risk of bias items for **(A)** Ketamine and **(B)** Esketamine included studies.

Figures 2, 3 summarize the risk of bias for all included studies, indicating that a certain degree of bias was present across the included trials. Notably, performance bias (i.e., blinding of participants and personnel) and detection bias (i.e., blinding of outcome assessment) were rated as unclear or high risk of bias in over half of the included studies. Moreover, 22 studies were directly industry-sponsored or authors had potential links to pharmaceutical industry, introducing other biases.

### 3.4 Primary safety outcomes of ketamine

#### 3.4.1 Dropouts due to AEs

A total of 27 participants dropped out of the trials prematurely due to AEs, with 23 in the ketamine group and four in the control group. Overall, ketamine was associated with a significantly higher dropout rate (RR = 4.06, 95% CI [1.63, 10.11], *p* = 0.003). Heterogeneity was low (*I* ^*2*^ = 0%, *p* = 0.96). Subgroup analysis included three placebo-controlled, one ECT-controlled and one midazolam-controlled studies. We verified that ketamine significantly increased the dropout rate compared to ECT, while no significant difference were found among other subgroup comparisons (Supplementary Figure S1).

#### 3.4.2 Number of participants experiencing at least one AE

Overall, there was a significant difference in the number of participants experiencing at least one AE between ketamine and control groups (RR = 1.36, 95% CI [1.01, 1.84], *p* = 0.04). Heterogeneity was *I* ^*2*^ = 89% (*p* < 0.00001). Sensitivity analysis showed no significant changes in heterogeneity (Supplementary Table S3). To further explore sources of heterogeneity, subgroup analyses were conducted by dividing the comparators into placebo, midazolam, and ECT. Ketamine significantly increased the incidence of AEs compared with placebo, but not with midazolam or ECT. However, heterogeneity remained substantial (Supplementary Figure S2).

#### 3.4.3 Number of participants experiencing at least one serious AE

No significant difference was found between ketamine and control in the number of participants experiencing at least one serious AE (RR = 0.68, 95% CI [0.42, 1.11], *p* = 0.12). Heterogeneity was *I* ^*2*^ = 0% (*p* = 0.85). Moreover, subgroup analysis showed no significant effects of comparators on heterogeneity and meta-analysis outcomes (Supplementary Figure S3).

#### 3.4.4 Number of participants experiencing the specific AEs

**FIGURE 4:**
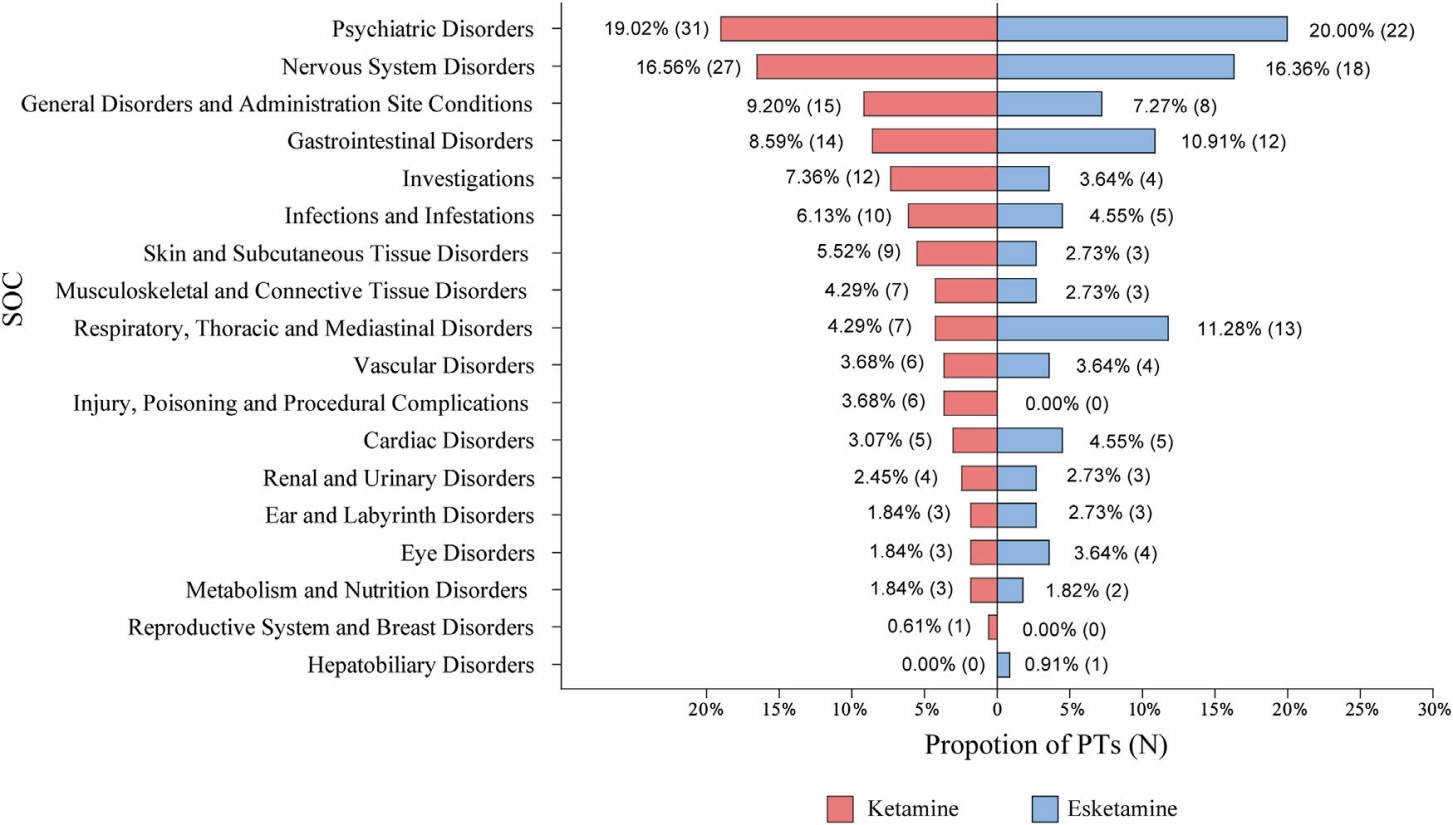
The categories of PTs reported in randomized controlled trials. AEs were coded with the PTs through the Medical Dictionary for Regulatory Activities and then categorized by SOC. AEs, adverse events; PTs, preferred terms; SOC, system organ class; N, number of PTs.

The most commonly reported AEs across all categories in the ketamine group were psychiatric disorders and nervous system disorders, which also applied to esketamine (Figure 4). Among 163 PTs, the five most common were dizziness (number of participants = 211, incidence rate = 18.84%), dissociation (202, 18.04%), nausea (149, 13.30%), anxiety (134, 11.96%), and vertigo (123, 10.98%). Table 1 presents the detailed results. We next performed meta-analyses for each specific AEs, showing that ketamine significantly increased the incidence of 25 AEs, including dissociation, dizziness, vision blurred, anxiety, vertigo, nausea, paralysis, salivary hypersecretion, euphoric mood, depersonalisation/derealisation disorder, restlessness, diplopia, hyperhidrosis, moodiness, chest discomfort, dysgeusia, movement disorder, constipation, paraesthesia, feeling hot, dyspnoea, affect lability, emotional disorder, hypoaesthesia, and autonomic nervous system imbalance compared to the control. We found no statistically significant differences for other AEs (Supplementary Table S4). Subgroup analyses of ketamine-reported AEs by comparators (placebo, midazolam, and ECT) are shown in online Supplementary Table S5.

**TABLE 1:** The commonly reported AEs in participants treated with ketamine.

SOC	PTs	Ketamine		Control	
		N	Incidence rate (%)	N	Incidence rate (%)
Nervous System Disorders	Dizziness	211	18.84	61	7.41
Psychiatric Disorders	Dissociation	202	18.04	34	4.13
Gastrointestinal Disorders	Nausea	149	13.30	68	8.26
Psychiatric Disorders	Anxiety	134	11.96	45	5.47
Ear and Labyrinth Disorders	Vertigo	123	10.98	40	4.86
General Disorders and Administration Site Conditions	Fatigue	106	9.46	72	8.75
Eye Disorders	Vision blurred	102	9.11	16	1.94
Gastrointestinal Disorders	Dry mouth	89	7.95	39	4.74
Psychiatric Disorders	Confusional state	74	6.61	35	4.25
Psychiatric Disorders	Restlessness	71	6.34	32	3.89
Nervous System Disorders	Paralysis	63	5.63	12	1.46
Gastrointestinal Disorders	Vomiting	56	5.00	26	3.16
Skin and Subcutaneous Tissue Disorders	Hyperhidrosis	43	3.84	15	1.82
Gastrointestinal Disorders	Salivary hypersecretion	42	3.75	2	0.24
Psychiatric Disorders	Euphoric mood	40	3.57	1	0.12
Psychiatric Disorders	Depersonalisation/derealisation disorder	39	3.48	7	0.85
Ear and Labyrinth Disorders	Tinnitus	35	3.13	15	1.82
Psychiatric Disorders	Dissociative disorder	33	2.95	0	0.00
Nervous System Disorders	Disturbance in attention	31	2.77	11	1.34
Nervous System Disorders	Dysgeusia	31	2.77	13	1.58
Eye Disorders	Diplopia	29	2.59	2	0.24
General Disorders and Administration Site Conditions	Feeling cold	28	2.50	16	1.94
General Disorders and Administration Site Conditions	Feeling hot	26	2.32	12	1.46
General Disorders and Administration Site Conditions	Malaise	26	2.32	19	2.31
Gastrointestinal Disorders	Diarrhoea	25	2.23	12	1.46
General Disorders and Administration Site Conditions	Decreased energy	25	2.23	14	1.70
Investigations	Blood pressure increased	22	1.96	5	0.61
Cardiac Disorders	Palpitations	22	1.96	9	1.09
Psychiatric Disorders	Moodiness	21	1.88	2	0.24
General Disorders and Administration Site Conditions	Chest discomfort	21	1.88	5	0.61
Nervous System Disorders	Movement Disorder	20	1.79	5	0.61
Psychiatric Disorders	Depression	17	1.52	3	0.36
Nervous System Disorders	Dizziness postural	17	1.52	11	1.34
Gastrointestinal Disorders	Constipation	17	1.52	4	0.49
Nervous System Disorders	Paraesthesia	16	1.43	4	0.49
Skin and Subcutaneous Tissue Disorders	Dry skin	16	1.43	6	0.73
Nervous System Disorders	Ataxia	15	1.34	6	0.73
Nervous System Disorders	Tremor	15	1.34	7	0.85
Respiratory, Thoracic and Mediastinal Disorders	Dyspnoea	15	1.34	4	0.49
Psychiatric Disorders	Insomnia	14	1.25	7	0.85
Psychiatric Disorders	Affect lability	13	1.16	3	0.36
General Disorders and Administration Site Conditions	Feeling abnormal	13	1.16	2	0.24
Skin and Subcutaneous Tissue Disorders	Pruritus	13	1.16	3	0.36

The commonly reported AEs for ketamine in the table are defined as those with an incidence rate greater than 1%. Simultaneously, the incidence of each AE induced by ketamine is higher than that in the control group. AEs were coded with the PTs through the Medical Dictionary for Regulatory Activities and then categorized by SOC. AEs, adverse events; PTs, preferred terms; SOC, system organ class; N, number of participants reporting relevant PT.

#### 3.4.5 Additional subgroup analyses and GRADE assessment

To evaluate the effect of dose or administration frequency on the occurrence of AEs, we further conducted subgroup analyses comparing the primary outcomes of ketamine. We found no significant differences in any of the comparisons between repeated- and single-dosing groups (Supplementary Table S6). In dose subgroup analyses, significantly higher rates of both overall and specific AEs were observed with relative high-dose ketamine, but not with serious AEs (Supplementary Table S7). According to the GRADE assessments, the evidence quality for the primary outcomes of dropouts due to AEs, number of patients experiencing at least one AE, or one serious AE was rated as moderate, low, or moderate certainty, respectively (Supplementary Table S8). Publication bias for ketamine’s primary safety outcomes could not be assessed due to insufficient studies in each meta-analysis.

### 3.5 Primary safety outcomes of esketamine

#### 3.5.1 Dropouts due to AEs

A total of 147 patients discontinued due to AEs, with 86 in the esketamine group and 61 in the control group. The esketamine group showed a significantly higher dropout rate due to AEs compared with the control group (RR = 1.85, 95% CI [1.01, 3.40], *p* = 0.05). Heterogeneity was *I* ^*2*^ = 53% (*p* = 0.009). Subgroup analysis included 13 placebo-controlled and one quetiapine-controlled studies. Esketamine significantly increased the dropout rate versus. placebo (RR = 2.21, 95% CI [1.39, 3.49], *p* = 0.0007). In contrast, significantly fewer esketamine-treated participants discontinued due to AEs compared to quetiapine. Moreover, heterogeneity dropped to non-significant levels (Supplementary Figure S4).

#### 3.5.2 Number of participants experiencing at least one AE

More participants receiving esketamine than controls reported at least one AE (RR = 1.35, 95% CI [1.22, 1.49], *p* < 0.00001). Heterogeneity was *I* ^*2*^ = 78% (*p* < 0.00001). Subgroup analyses by comparators showed that the effect sizes remained significant when comparing esketamine with either placebo or quetiapine. However, heterogeneity remained unchanged (Supplementary Figure S5). Further sensitivity analysis identified the study by Daly et al. (2019) as an outlier, significantly influencing the heterogeneity (Supplementary Table S9). This may be due to differences in recruitment, as the outlier study recruited patients pre-exposed to esketamine to reach the response or remission status.

#### 3.5.3 Number of participants experiencing at least one serious AE

We found no significant difference between esketamine and control in the number of participants experiencing at least one serious AE (RR = 1.14, 95% CI [0.74, 1.73], *p* = 0.56). Heterogeneity was *I* ^*2*^ = 0% (*p* = 0.95). Moreover, subgroup analysis showed no impact of comparators on heterogeneity or effect sizes (Supplementary Figure S6).

#### 3.5.4 Number of participants experiencing the specific AEs

Esketamine was associated with 110 distinct PTs. Among them, the five most common were dizziness (583, 35.38%), dissociation (471, 28.58%), nausea (418, 25.36%), headache (323, 19.60%), and somnolence (272, 16.50%). Table 2 presents the detailed results. Meta-analyses were conducted for each AE. Among psychiatric disorders, subjects receiving esketamine were more likely to report confusion, depersonalization/derealisation disorder, dissociation, and euphoric mood. Across nervous system disorders, esketamine significantly increased the incidence of dizziness, dizziness postural, dysgeusia, headache, hypoaesthesia, mental impairment, paraesthesia, sedation, and somnolence. For gastrointestinal disorders, esketamine was more likely to cause hypoaesthesia oral, nausea, paraesthesia oral, and vomiting compared to the control. Other AEs were also significant for esketamine, including asthenia, feeling drunk, vertigo, diplopia, vision blurred, blood pressure increased, tachycardia, and throat irritation. No significant differences were found for other AEs (Supplementary Table S10). Subgroup analyses of esketamine-reported AEs by comparators (placebo and quetiapine) are shown in online Supplementary Table S11.

**TABLE 2:** The commonly reported AEs in participants treated with esketamine.

SOC	PTs	Esketamine		Control	
		N	Incidence rate (%)	N	Incidence rate (%)
Nervous System Disorders	Dizziness	583	35.38	124	8.52
Psychiatric Disorders	Dissociation	471	28.58	44	3.02
Gastrointestinal Disorders	Nausea	418	25.36	101	6.94
Nervous System Disorders	Headache	323	19.60	179	12.30
Nervous System Disorders	Somnolence	272	16.50	153	10.52
Nervous System Disorders	Dysgeusia	230	13.96	91	6.25
Ear and Labyrinth Disorders	Vertigo	230	13.96	20	1.37
Investigations	Blood pressure increased	193	11.71	47	3.23
Gastrointestinal Disorders	Vomiting	156	9.47	31	2.13
Nervous System Disorders	Paraesthesia	142	8.62	23	1.58
Nervous System Disorders	Hypoaesthesia	138	8.37	13	0.89
Eye Disorders	Vision blurred	135	8.19	25	1.72
Gastrointestinal Disorders	Hypoaesthesia oral	95	5.76	6	0.41
Nervous System Disorders	Sedation	89	5.40	38	2.61
Psychiatric Disorders	Anxiety	82	4.98	43	2.96
General Disorders and Administration Site Conditions	Fatigue	68	4.13	59	4.05
Psychiatric Disorders	Insomnia	61	3.70	51	3.51
Nervous System Disorders	Dizziness postural	60	3.64	7	0.48
Respiratory, Thoracic and Mediastinal Disorders	Throat irritation	51	3.09	15	1.03
Gastrointestinal Disorders	Paraesthesia oral	49	2.97	8	0.55
Respiratory, Thoracic and Mediastinal Disorders	Nasal discomfort	48	2.91	26	1.79
General Disorders and Administration Site Conditions	Feeling drunk	44	2.67	5	0.34
Psychiatric Disorders	Euphoric mood	43	2.61	6	0.41
Gastrointestinal Disorders	Diarrhoea	38	2.31	19	1.31
Psychiatric Disorders	Confusion	29	1.76	1	0.07
Gastrointestinal Disorders	Dry mouth	26	1.58	14	0.96
Gastrointestinal Disorders	Constipation	25	1.52	19	1.31
Infections and Infestations	Nasopharyngitis	23	1.40	11	0.76
Eye Disorders	Diplopia	21	1.27	0	0.00
General Disorders and Administration Site Conditions	Asthenia	20	1.21	2	0.14
Nervous System Disorders	Mental impairment	17	1.03	1	0.07
Musculoskeletal and Connective Tissue Disorders	Back pain	17	1.03	9	0.62

The commonly reported AEs for esketamine in the table are defined as those with an incidence rate greater than 1%. Simultaneously, the incidence of each AE induced by esketamine is higher than that in the control group. AEs were coded with the PTs through the Medical Dictionary for Regulatory Activities and then categorized by SOC. AEs, adverse events; PTs, preferred terms; SOC, system organ class; N, number of participants reporting relevant PT.

#### 3.5.5 Additional subgroup analyses and GRADE assessment

To evaluate the effect of dose on the occurrence of AEs, we further conducted subgroup analyses comparing the primary outcomes of esketamine. In dose subgroup analyses, significantly higher rates of dropout due to AEs and overall AEs were observed with relative high-dose esketamine, but not with serious AEs (Supplementary Table S12). Moreover, esketamine significantly increased the incidence of dizziness, hypoaesthesia oral, hypoaesthesia, vertigo, and blood pressure increased across all dose groups, while other AEs were more likely to report in the relative high-dose group. According to the GRADE assessments, the evidence quality for the primary outcomes of esketamine was rated as high, low, or moderate certainty, respectively (Supplementary Table S13).

#### 3.5.6 Publication bias

**FIGURE 5:**
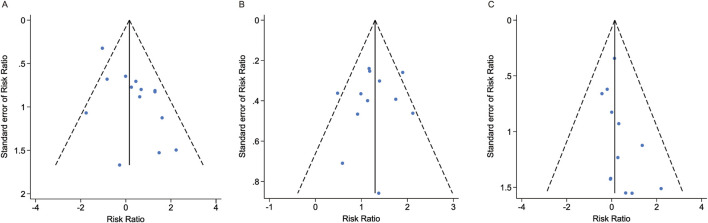
Funnel plots and publication bias tests for three primary outcomes of esketamine. **(A)** Dropout rate due to AEs. **(B)** Number of participants experiencing at least one AE. **(C)** Number of participants experiencing at least one serious AE. AE, adverse event.

We conducted publication bias tests for three primary outcomes of esketamine. Both visual inspection of the funnel plots (Figure 5) and Egger’s tests showed no significant publication bias in the meta-analyses. The results of Egger’s tests for dropouts due to AEs, number of participants with at least one AE, and number of participants with at least one serious AE were Pr > |z| = 0.105, 0.646, and 0.130, respectively.

### 3.6 NNH analysis for the primary safety outcomes of ketamine and esketamine

For ketamine versus placebo, both dropout and incidence rates of AEs were statistically significant, with NNH values (ARI [95% CI]) of 12 (8.44%, [0.78%–16.09%]) and 2 (46.52%, [31.66%–61.38%]), respectively. Conversely, NNH analysis for the incidence rate of serious AEs was insignificant. A similar pattern of effect sizes was found when comparing esketamine with placebo, but with higher corresponding NNH values (Table 3). The detailed NNH values for esketamine- and ketamine-associated AEs are shown in Tables 3, 4, respectively.

**TABLE 3:** The NNT results of the primary safety outcomes for esketamine.

Outcomes	Event rates (%)		ARI		NNH^a^
	Esketamine group	Control group	%	95% CI	
Dropout due to AEs	5.09	4.22	0.87	[ns]^d^	N/A
Esketamine vs. Placebo^b^	5.31	2.16	3.15	[1.68–4.62]	32
Esketamine vs. Quetiapine^b^	4.19	11.01	-6.82	[ns]^d^	N/A
Number of participants experiencing at least one AE	85.18	64.57	20.60	[17.42–23.78]	5
Esketamine vs. Placebo^b^	83.26	59.77	23.50	[19.7–27.29]	4
Esketamine vs. Quetiapine^b^	91.92	77.98	13.94	[8.63–19.25]	7
Number of participants experiencing at least one serious AE	3.37	2.94	0.44	[ns]^d^	N/A
Esketamine vs. Placebo^b^	2.72	2.19	0.52	[ns]^d^	N/A
Esketamine vs. Quetiapine^b^	5.69	5.06	0.63	[ns]^d^	N/A
Treatment-emergent AEs^c^					
Dizziness	35.38	8.52	26.85	[24.14–29.57]	4
Dissociation	28.58	3.02	25.56	[23.20–27.91]	4
Nausea	25.36	6.94	18.42	[15.95–20.90]	5
Vertigo	13.96	1.37	12.58	[10.80–14.36]	8
Blood pressure increased	11.71	3.23	8.48	[6.68–10.28]	12
Dysgeusia	13.96	6.25	7.70	[5.62–9.79]	13
Hypoaesthesia	8.37	0.89	7.48	[6.06–8.90]	13
Vomiting	9.47	2.13	7.34	[5.74–8.93]	14
Headache	19.60	12.30	7.30	[4.74–9.85]	14
Paraesthesia	8.62	1.58	7.04	[5.54–8.53]	14
Vision blurred	8.19	1.72	6.47	[4.99–7.96]	15
Somnolence	16.50	10.52	5.99	[3.60–8.38]	17
Hypoaesthesia oral	5.76	0.41	5.35	[4.18–6.52]	19
Dizziness postural	3.64	0.48	3.16	[2.19–4.13]	32
Sedation	5.40	2.61	2.79	[1.42–4.15]	36
Paraesthesia oral	2.97	0.55	2.42	[1.52–3.33]	41
Feeling drunk	2.67	0.34	2.33	[1.49–3.16]	43
Euphoric mood	2.61	0.41	2.20	[1.36–3.03]	46
Throat irritation	3.09	1.03	2.06	[1.08–3.05]	48
Confusion	1.76	0.07	1.69	[1.04–2.34]	59
Diplopia	1.27	0.00	1.27	[0.73–1.82]	78
Asthenia	1.21	0.14	1.08	[0.51–1.64]	93
Mental impairment	1.03	0.07	0.96	[0.46–1.47]	104
Depersonalisation/Derealisation disorder	0.61	0.00	0.61	[0.23–0.98]	165

^a^ NNH was calculated only when the 95% confidence intervals for the risk difference excluded the null. By convention, NNH values were rounded up to the next whole number.

^b^ The subgroup analyses of the primary outcomes were conducted by dividing the controls into placebo and quetiapine.

^c^ The threshold for calculating the NNHs for spontaneously reported AEs was that the incidences were significantly higher in the esketamine group than those reported in the control group from the meta-analyses.

^d^ ns: not significant at the *p* < 0.05 threshold, thus the 95% CI, is not shown.

N/A, not available; ARI, absolute risk increase; AE adverse events; CI, confidence interval; NNH, number needed to harm.

**TABLE 4:** The NNT results of the primary safety outcomes for ketamine.

Outcomes	Event rates (%)		ARI		NNH^a^
	Ketamine group	Control group	%	95% CI	
Dropout due to AEs	9.16	1.65	7.52	[3.61–11.43]	13
Ketamine vs. Placebo^b^	10.00	1.56	8.44	[0.78–16.09]	12
Ketamine vs. ECT^b^	12.63	3.30	9.33	[1.71–16.96]	11
Ketamine vs. Midazolam^b^	4.65	0.00	4.65	[0.20–9.10]	22
Number of participants experiencing at least one AE	73.24	63.88	9.37	[1.35–17.38]	11
Ketamine vs. Placebo^b^	84.62	38.10	46.52	[31.66–61.38]	2
Ketamine vs. ECT^b^	93.41	94.44	−1.04	[ns]^d^	N/A
Ketamine vs. Midazolam^b^	55.24	48.65	6.60	[ns]^d^	N/A
Number of participants experiencing at least one serious AE	4.91	8.48	−3.57	[ns]^d^	N/A
Ketamine vs. Placebo^b^	3.91	1.20	2.71	[ns]^d^	N/A
Ketamine vs. ECT^b^	15.38	25.56	−10.17	[ns]^d^	N/A
Ketamine vs. Midazolam^b^	2.33	4.17	−1.83	[ns]^d^	N/A
Treatment-emergent AEs^c^					
Dissociation	18.04	4.13	13.90	[11.27–16.53]	7
Dizziness	18.84	7.41	11.43	[8.52–14.33]	9
Vision blurred	9.11	1.94	7.16	[5.23–9.09]	14
Anxiety	11.96	5.47	6.50	[4.04–8.95]	15
Vertigo	10.98	4.86	6.12	[3.77–8.47]	16
Nausea	13.30	8.26	5.04	[2.30–7.78]	20
Paralysis	5.63	1.46	4.17	[2.59–5.75]	24
Salivary hypersecretion	3.75	0.24	3.51	[2.34–4.67]	29
Euphoric mood	3.57	0.12	3.45	[1.39–4.56]	29
Depersonalisation/Derealisation disorder	3.48	0.85	2.63	[1.39–3.88]	38
Restlessness	6.34	3.89	2.45	[0.51–4.40]	41
Diplopia	2.59	0.24	2.35	[1.36–3.34]	43
Hyperhidrosis	3.84	1.82	2.02	[0.57–3.47]	50
Moodiness	1.88	0.24	1.63	[0.77–2.49]	61
Chest discomfort	1.88	0.61	1.27	[0.31–2.22]	79
Dysgeusia	2.77	1.58	1.19	[ns]^d^	N/A
Movement disorder	1.79	0.61	1.18	[0.24–2.12]	85
Constipation	1.52	0.49	1.03	[0.17–1.89]	97
Paraesthesia	1.43	0.49	0.94	[0.10–1.78]	106
Feeling hot	2.32	1.46	0.86	[ns]^d^	N/A
Dyspnoea	1.34	0.49	0.85	[0.03–1.68]	117
Affect lability	1.16	0.36	0.80	[0.05–1.55]	126
Emotional disorder	0.71	0.00	0.71	[0.22–1.21]	140
Hypoaesthesia	0.71	0.00	0.71	[0.22–1.21]	140
Autonomic nervous system imbalance	0.89	0.36	0.53	[ns]^d^	N/A

^a^ NNH was calculated only when the 95% confidence intervals for the risk difference excluded the null. By convention, NNH values were rounded up to the next whole number.

^b^ The subgroup analyses of the primary outcomes were conducted by dividing the controls into placebo, midazolam, and ECT.

^c^ The threshold for calculating the NNHs for spontaneously reported AEs was that the incidences were significantly higher in the ketamine group than those reported in the control group from the meta-analyses.

^d^ ns: not significant at the *p* < 0.05 threshold, thus the 95% CI, is not shown.

N/A, not available; ARI, absolute risk increase; AEs, adverse events; CI, confidence interval; ECT, electroconvulsive therapy; NNH, number needed to harm.

### 3.7 Secondary safety outcomes of ketamine and esketamine

#### 3.7.1 Psychiatric or psychotomimetic side-effects

**FIGURE 6:**
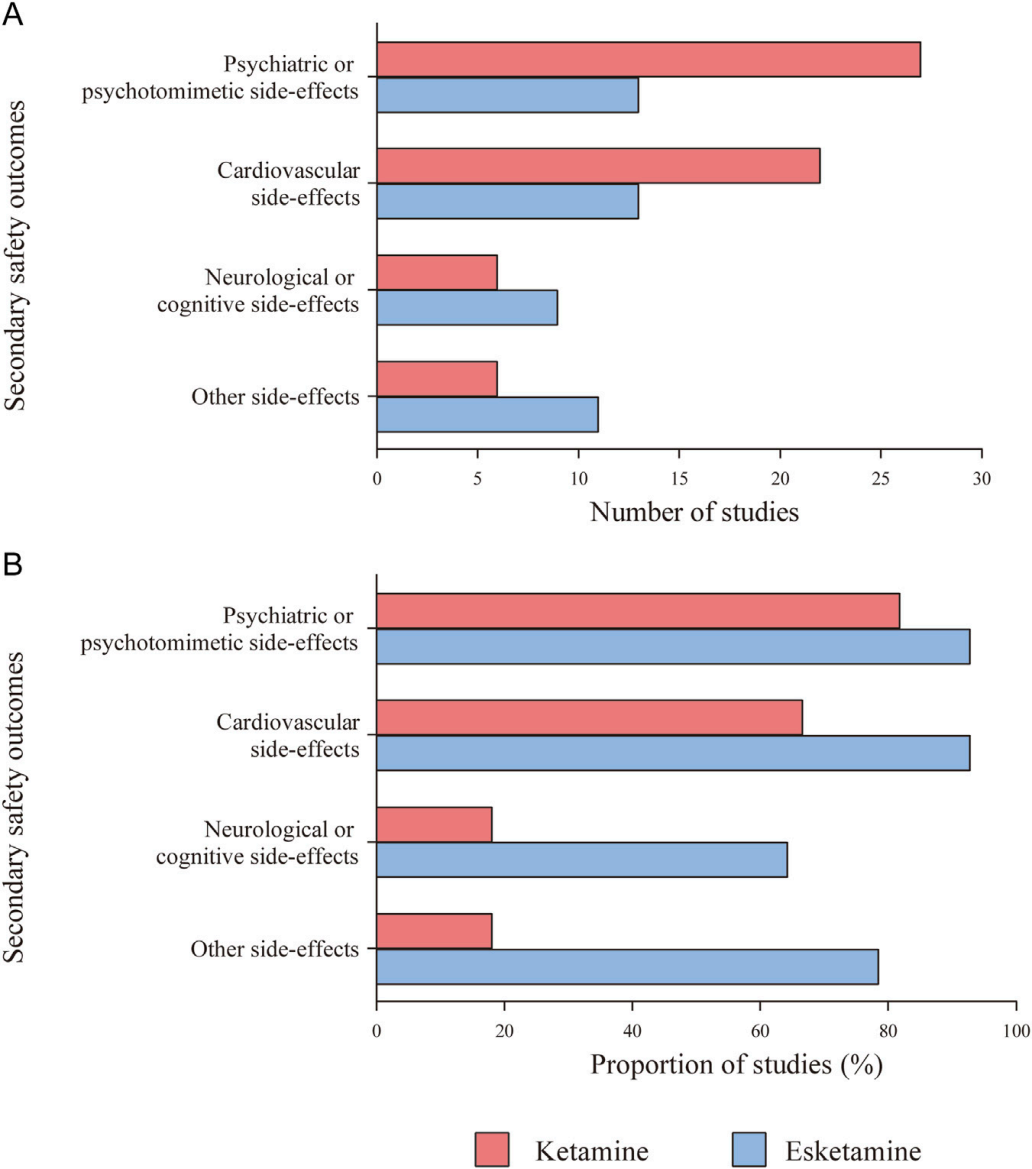
Use of structured side-effect assessment scales and categories. **(A)** Number of studies that used relevant structured scales or questionnaires to describe assessment for secondary safety outcomes. **(B)** Proportion of studies that used relevant structured scales or questionnaires to describe assessment for secondary safety outcomes.

Treatment-emergent psychiatric or psychotomimetic side-effects (e.g., dissociative symptoms, psychotic symptoms, elevated mood, and suicidal ideation) were reported in 27 (81.82%) ketamine studies and 13 (92.86%) esketamine studies (Figure 6). Ketamine significantly increased clinician administered dissociative states scale (CADSS) scores at 40 min (MD = 6.83, 95% CI [2.71, 10.94], *p* = 0.001) and 60 min (MD = 6.50, 95% CI [4.42, 8.58], *p* < 0.00001) post-infusion, with no differences thereafter (Supplementary Table S14). Moreover, the effects were dose-dependent, being significant at doses of 0.5 mg/kg or higher, but not at lower doses (Supplementary Table S15). Esketamine significantly increased CADSS score at 40 min and 1.5 h post-administration (Supplementary Table S16), with similar effects observed at higher doses (56–84 mg) in subgroup analysis (Supplementary Table S17). Both drugs transiently and significantly increased the brief psychiatric rating scale positive symptom subscale (BPRS+) scores, ketamine from 10 to 60 min (Supplementary Table S18) and esketamine from 30 min to 1.5 h (Supplementary Figure S7) post-administration. Elevated Young mania rating scale (YMRS) scores were observed only with ketamine at 40 min (Supplementary Figure S8), and ketamine increased the visual analogue scale score for intoxication ‘high’ (VAS-high) scores at 10 and 40 min post-dose (Supplementary Figure S9).

#### 3.7.2 Cardiovascular side-effects

Treatment-emergent hemodynamic changes, including systolic blood pressure (SBP), diastolic blood pressure (DBP), heart rate, respiratory rate, oxygen saturation, and electrocardiogram (ECG), were reported in 22 ketamine and 13 esketamine studies (Figure 6). Ketamine significantly elevated SBP and DBP at 15–60 min post-dose (Supplementary Table S19), with significantly more participants exceeding predefined hypertensive thresholds (peak SBP ≥180 mmHg or DBP ≥110 mmHg) (Supplementary Table S20). Esketamine produced similar BP increases up to 1.5 h (Supplementary Table S21), without excessively exceeding the severe hypertensive thresholds (Supplementary Table S22). No significant differences in heart rate changes were observed for either drug (Supplementary Figures S10, S11). Ketamine significantly reduced respiratory rate at 40 min (Supplementary Figure S12), whereas oxygen saturation changes remained within normal ranges in both groups (Supplementary Figures S13, S14). Esketamine did not differ from placebo in any ECG outcome (Supplementary Table S23).

#### 3.7.3 Neurological or cognitive side-effects

Treatment-emergent neurological or cognitive side-effects, including sedation and cognition impairment, were reported in 6 (18.18%) ketamine studies and 9 (64.29%) esketamine studies (Figure 6). Sedation severity was classified using the modified observer’s assessment of alertness/sedation (MOAA/S) scale: ≤4 score (any sedation), ≤3 (moderate), ≤2 (severe) (Fu et al., 2020). According to the MOAA/S score, esketamine significantly increased sedation across all severity levels (Supplementary Figure S15).

Six studies assessed ketamine’s cognitive effects, with three contributing data for meta-analyses. The timing of post-dose measurements varied. One study measured 24-h post-baseline neurocognitive changes across multiple domains, including attention, memory, processing speed, language skills, and concentration (Keilp et al., 2021). Another assessed the MATRICS consensus cognitive battery scores on Day 7 (Murrough et al., 2015). The third used the CogState test to assess multiple cognitive domains (e.g., attention, processing speed, memory, visual learning, and executive function) on Day 28 following repeated dosing (Gálvez et al., 2018). Overall, there were no cognitive differences between ketamine and control in 21 independent comparisons, except for one that favored ketamine in delayed memory 24 h post-dose, as assessed by the Buschke Selective Reminding Test (Supplementary Table S24).

A study using the Massachusetts General Hospital-cognitive and physical functioning questionnaire assessed the overall impact of esketamine on subjects 1 day post-dose (Singh et al., 2016a). Another evaluated cognitive performance on Day 28 using the Hopkins verbal learning test (revised) and CogState computerized cognitive battery (Ochs-Ross et al., 2020). The third estimated the cognitive function of participants by the Montreal cognitive assessment scale (MoCA) during the 2-month treatment period and 6-month follow-up (Hong et al., 2025). Esketamine significantly improved cognitive and physical function at 24 h post-dose, with no significant cognitive differences for other comparisons on Day 28. The most surprising finding revealed by MoCA data is that esketamine significantly improved cognitive function during the 1- to 8-month assessment period (Supplementary Table S25).

#### 3.7.4 Other side-effects

Other potential side-effects, including laboratory abnormalities, bladder symptoms, withdrawal symptoms, nasal symptoms, and abuse/addiction, were reported in 6 (18.18%) ketamine studies and 11 (78.57%) studies (Figure 6). No significant laboratory abnormalities were detected for esketamine treatment (Supplementary Figure S16). No data were available for ketamine. Ketamine did not significantly worsen bladder symptoms at Week 4 post-dose, as assessed by bladder pain/interstitial cystitis symptom score (BPIC-SS), and esketamine data were lacking (Supplementary Figure S17). There was no significant difference between the esketamine and control group in the number of patients with nasal discomfort, as assessed by nasal examination (Supplementary Figure S18) and nasal symptoms questionnaire (Supplementary Figure S19), respectively. Esketamine was associated with delayed discharge readiness at 1–1.5 h, assessed by clinical global assessment of discharge readiness (CGADR) scale (Supplementary Figure S20), and with transient withdrawal symptoms (e.g., fatigue, restlessness) during the first 1–2 weeks post-discontinuation (Supplementary Table S26). Given the lack of quantitative data to assess abuse liability, a narrative description of the results was provided in this part. In total, six studies preliminarily suggested no signs of esketamine/ketamine abuse or addiction in depression treatment. Three studies reported no requests to increase the esketamine dose and/or frequency during treatment (Canuso et al., 2018; Daly et al., 2019; Popova et al., 2019), while another two reported no symptoms of esketamine craving during follow-up (Ionescu et al., 2021; Takahashi et al., 2021). A study on ketamine reported that during the 4-week treatment period, three subjects reported craving for the study drug: 2 in the ketamine group and 1 in the midazolam group. Moreover, no subjects in either group reported using ketamine outside of supervised medical or research settings (Loo et al., 2023).

## 4 Discussion

Previous reviews have consistently demonstrated that both racemic ketamine and esketamine are associated with higher rates of AEs in depression treatment. However, the incidence of serious AEs and treatment discontinuation due to AEs remains relatively low, most of which are mild-to-moderate and resolve on the day of administration (Ceban et al., 2021; Rhee et al., 2022; Short et al., 2018). Our findings are broadly consistent with the existing evidence. Further subgroup analyses revealed significant differences in primary safety outcomes for both drugs based on dose stratification, but not between single and repeated ketamine dosing. Therefore, an implication of these findings is the possibility that the administered dose, rather than cumulative exposure, may increase the risk of adverse effects occurring in subjects during treatment. Another important finding is that the categories of treatment-emergent AEs observed during ketamine and esketamine treatments for depression were generally identical, with a discrepancy in the percentage, further supporting evidence from previous studies (Ceban et al., 2021; McIntyre et al., 2021). This discrepancy may be attributed to multiple factors, including the heterogeneity in ketamine formulations, administration routes and doses, patient populations, concomitant medications, and receptor binding affinity (McIntyre et al., 2021; Yang et al., 2019). Another noteworthy factor is the methodological disparity in AEs reporting. Most included AEs in ketamine trials are not reported systematically and are likely subject to the reporting bias of participants. This stands in distinct contrast to the safety and tolerability data for esketamine, which primarily relied on systematic data collection methods (McIntyre et al., 2021; Short et al., 2018). Importantly, through dose-subgroup stratification and NNH analyses, we further confirm that a series of treatment-emergent AEs, such as dizziness, dissociation, nausea, vertigo, dysgeusia, hypoesthesia, paraesthesia, vision blurred, euphoric mood, and diplopia, with relatively low NNH values, would be more likely to occur in clinical practice and exhibit dose-dependent effects. Therefore, our study adds a clinically relevant perspective to existing literature by applying the NNH metric, highlighting which adverse effects are more likely to occur in routine clinical practice, thus complementing conventional AEs reporting.

Our analysis also extends previous work by differentiating the tolerability profiles of ketamine and esketamine. We found that esketamine was associated with significantly more AE-related dropouts than placebo, whereas ketamine was not. In contrast to an earlier review, suggesting no evidence of significant differences in dropout rates when comparing ketamine or esketamine to placebo, respectively (Dean et al., 2021). Moreover, both drugs had statistically significant dropouts due to AEs within NNH analysis, with a higher value observed for eketamine. This contrasts with Calder et al. (2024), who reported the opposite pattern. This discrepancy likely reflects differences in how dropout rates were assessed across studies, with previous studies counting the overall number of people who dropped out during the trial regardless of being due to lack of efficacy or side-effects. Overall, our review suggests that short-term esketamine use may be slightly better tolerated than ketamine, further supporting the notion that statistically significant results in systematic review and meta-analysis do not necessarily translate into clinical significance (Wong et al., 2024).

Both ketamine and esketamine were associated with transient and significantly psychiatric side-effects, with dissociative symptoms the most prominent. Ketamine significantly increased CADSS, BPRS+, YMRS, and VAS-high scores within the first hour post-infusion, but not at later time points. However, esketamine-related symptoms appeared to persist up to 1.5 h, suggesting a delayed effect compared to ketamine changes. Additionally, both drugs exhibited dose-dependent effects on CADSS scores, with significant effects only observed at higher doses. This further support previous findings that dissociative symptoms are dose-dependent, with the incidence and intensity diminishing following repeated infusions (Ceban et al., 2021; McIntyre et al., 2021).

Ketamine induced statistically significant changes in hemodynamic parameters at 15–60 min post-dose, with peak increases in SBP and DBP of 13.64 and 8.97 mm Hg, respectively, and a reduction in respiratory rate of 1.73 breaths/min at 40 min. Esketamine produced similar SBP and DBP elevations, persisting up to 1.5 h, with peak increases of 9.24 and 7.27 mm Hg, respectively. This finding is consistent with a previous meta-analysis indicating that esketamine and subanesthetic dose of ketamine induce mild but significant elevations in BP among adult psychiatric patients, with this increase being transient in nature (Vankawala et al., 2021). Moreover, participants receiving ketamine were more likely to exceed the thresholds of hypertensive urgency [SBP >180 or DBP >110 mm Hg (Gauer, 2017; Vankawala et al., 2021)], but not esketamine. A strong relationship between ketamine and BP increases has been reported in the previous literature, with BP exceeding 180/100 mm Hg affecting 20%–30% of ketamine-receiving patients (typically for TRD) (Short et al., 2018; Szarmach et al., 2019). It has also been reported that up to 20% of patients receiving ketamine treatment for TRD in a community clinic setting may require medication intervention to manage hypertension (Rodrigues et al., 2020). However, in the esketamine development program for TRD, the incidence of hypertension was relatively low, with 2.1% of patients requiring antihypertensive treatment (Doherty et al., 2020). Overall, esketamine has a smaller and clinically insignificant effect size on BP changes compared to ketamine. However, the findings of the current study do not support a previous real-world pharmacovigilance research suggesting a higher risk of serious BP-related AEs with esketamine (Gastaldon et al., 2021). This discrepancy likely stems from excluding participants with unstable or untreated hypertension in esketamine trials, thus omitting susceptible individuals in this analysis (Janssen, 2020; Vankawala et al., 2021). Therefore, we strongly agree with expert guidance to monitor the participants’ vital signs for at least 2 hours before discharge (McIntyre et al., 2021).

Cognitive deficits affect around 40% of TRD patients, making the impacts of ketamine and esketamine on cognitive function clinically crucial (American Psychiatric Association, 2024; Gill et al., 2021). Preliminary evidence supports short-term cognitive safety of subanesthetic ketamine in depressed patients, though with limited data for esketamine (Dean et al., 2021; Marchi et al., 2022; Martin et al., 2024). A placebo-controlled study in healthy volunteers found no impaired driving performance (a cognitive proxy) 8 hours after 84 mg intranasal esketamine (van de Loo et al., 2017). Consistent with the literature, we found that ketamine and esketamine did not show significant effects from controls in most cognitive domains. Moreover, both drugs significantly outperformed the control in potential areas like delayed memory at 24 h post-dose. However, this contradicts several previous studies associating acute ketamine use in healthy volunteers with memory impairment and chronic recreational use with broader cognitive and psychological deficits (Morgan et al., 2004; Morgan et al., 2010). Furthermore, Ketamine adjunct to ECT treatment showed consistent cognitive decline in depressed patients (Loo et al., 2012). These discrepancies may stem from ketamine’s antidepressant effect mitigating its cognitive harm (Marchi et al., 2022) and the known cognitive risks of ECT synergizing negatively when combined (Loo et al., 2012; Marchi et al., 2022). Therefore, we excluded ketamine-ECT combination trials in this review to focus on drug effects alone. Overall, no convincing evidence shows that ketamine or esketamine improves cognition over comparators, but their use in depressed patients appears unlikely to worsen it. Another notable neurological side-effect is sedation. In our analysis, esketamine significantly increased the incidence of sedation, including severe cases; in contrast, ketamine did not. This finding aligns with previous studies reporting that esketamine may induce severe sedative reactions (Gastaldon et al., 2021; Guo et al., 2022; McIntyre et al., 2021). This discrepancy may partially relate to ketamine’s higher anesthetic doses (1–4.5 mg/kg) compared to subanesthetic doses (usually ≤0.5 mg/kg) used in depression trials (U.S. Food and Drug Administration, 2022; Molero et al., 2018).

No significant abnormalities were observed in laboratory results, BPIC-SS, nasal examination, or addiction-related evaluations for either drug. CGADR data suggest esketamine-treated participants should remain under observation for at least 1.5 h post-dose, with most ready for discharge by 3 hours (Ochs-Ross et al., 2020; Popova et al., 2019). Furthermore, no definitive withdrawal symptoms were observed during 1–2 weeks post-esketamine discontinuation, and symptom changes appeared to mirror the fluctuations in depressive symptomatology (Popova et al., 2019; Takahashi et al., 2021). Overall, no compelling evidence links either drug to abuse, dependence, urinary or hepatic toxicity, or nasal side-effects in depression treatment.

This review has several limitations. Firstly, the data completeness of evidence in this review was limited. Despite comprehensive searches, limited data were available for several pre-specified outcomes, particularly for the secondary safety outcomes, including cognition, bladder symptoms, laboratory parameters, and abuse/addiction. Notably, several outcomes included only one or two studies with very few participants, resulting in extremely wide CIs for corresponding outcomes. The low precision and possible low statistical power may ultimately limit the reliability of these findings. Secondly, there were considerable variations in study design across trials (e.g., administration route, concomitant medications, dosing frequency, and duration), which potentially introduced methodological heterogeneity in trials and may impact the assessment of treatment effects. Thirdly, the quality of evidence was difficult to assess due to the potential risk of bias in many studies. Blinding-related performance and detection bias were rated unclear or high in almost half of the trials. Most ketamine trials lacked detailed descriptions of blinding procedures, and the psychotomimetic effects of both drugs may have compromised blinding, allowing participants to infer treatment allocation (Dean et al., 2021). Selective reporting bias was also evident due to missing study protocols or discrepancies between reported and pre-specified outcomes in many studies. Notably, many esketamine trials were industry-sponsored, potentially introducing other biases and limiting the reliability of the findings. Lastly, the NNH parameter is not a real effect size per se and enables no direct comparison of the effects between different treatments.

## 5 Conclusion

Although we have further provided promising evidence supporting the safety of ketamine and esketamine in depression treatment, the findings of this study highlight a potential tolerability advantage with esketamine over ketamine for short-term use in subjects with MDD. Furthermore, given that intravenous ketamine is resource-intensive and relatively inconvenient in routine clinical settings, esketamine appears to offer a more promising and practical short-term treatment option for depression. However, it is not possible to draw direct conclusions regarding the superiority of eketamine over ketamine in terms of safety and tolerability within this analysis. Therefore, there is an urgent need for additional large-scale and methodologically rigorous head-to-head clinical trials directly comparing these two drugs.

## Data Availability

The original contributions presented in the study are included in the article/Supplementary Material, further inquiries can be directed to the corresponding authors.
