# Supplementary material for: Comparative safety and tolerability of ketamine and esketamine for major depressive disorder: a systematic review and meta-analysis
Source: Front Pharmacol. 2025 Oct 29;16:1681060. doi: 10.3389/fphar.2025.1681060 (PMC12605052; doi:10.3389/fphar.2025.1681060)
Supplement: Supplementary file 1 [file DataSheet1.pdf]

Supplementary Material

Appendix 1. Supplementary tables and figures

Supplementary Table 1. The detailed characteristics of studies included in the meta-analysis
Supplementary Table 2. Reported measures of safety outcomes in included trials
Supplementary Table 3. Combined effect of ketamine after excluding each individual study in turn
Supplementary Table 4. Meta-analyses for each specific AE associated with ketamine
Supplementary Table 5. Subgroup analyses by comparators for each specific AE associated with ketamine
Supplementary Table 6. Subgroup analyses comparing the primary outcomes between repeated and single dosing of ketamine
Supplementary Table 7. Subgroup analyses comparing the primary outcomes between low-dose and very low-dose ketamine
Supplementary Table 8. The GRADE assessments of the evidence certainty for the primary outcomes of ketamine
Supplementary Table 9. Combined effect of esketamine after excluding each individual study in turn
Supplementary Table 10. Meta-analyses for each specific AE associated with esketamine
Supplementary Table 11. Subgroup analyses for each specific AEs associated with esketamine
Supplementary Table 12. Subgroup analyses by dose comparing the primary outcomes of esketamine.
Supplementary Table 13. The GRADE assessments of the evidence certainty for the primary outcomes of esketamine
Supplementary Table 14. Changes in ketamine-related CADSS scores from baseline to each predefined time point
Supplementary Table 15. Subgroup analyses of ketamine dose on changes in CADSS scores at 40 minutes post-dose
Supplementary Table 16. Changes in esketamine-related CADSS scores from baseline to each predefined visit time point
Supplementary Table 17. Subgroup analyses of esketamine dose on changes in CADSS scores at 40 minutes post-dose at each visit time point
Supplementary Table 18. Changes in ketamine-related BPRS+ scores from baseline to each predefined time point
Supplementary Table 19. Ketamine-related changes in SBP and DBP from baseline to each predefined time point
Supplementary Table 20. Meta-analyses for the number of participants with treatment-emergent SBP or DBP abnormalities after ketamine administration
Supplementary Table 21. Esketamine-related changes in SBP and DBP from baseline to each predefined visit time point
Supplementary Table 22. Meta-analyses for the number of participants with treatment-emergent SBP or DBP abnormalities after esketamine administration
Supplementary Table 23. Meta-analyses for the number of participants with treatment-emergent abnormal ECG values after esketamine administration
Supplementary Table 24. Meta-analyses for the effects of ketamine on cognitive performance
Supplementary Table 25. Meta-analyses for the effects of esketamine on cognitive performance
Supplementary Table 26. The potential withdrawal symptoms after cessation of esketamine treatment
Supplementary Figure 1. Forest plot of comparisons between ketamine and controls for the dropout

rate due to AEs

Supplementary Figure 2. Forest plot of comparisons between ketamine and controls for the number of participants experiencing at least one AE

Supplementary Figure 3. Forest plot of comparisons between ketamine and controls for the number of participants experiencing at least one serious AE

Supplementary Figure 4. Forest plot of comparisons between esketamine and controls for the dropout rate due to AEs

Supplementary Figure 5. Forest plot of comparisons between esketamine and controls for the number of participants experiencing at least one AE

Supplementary Figure 6. Forest plot of comparisons between esketamine and controls for the number of participants experiencing at least one serious AE

Supplementary Figure 7. Forest plot of changes in esketamine-related BPRS+ scores from baseline to each predefined visit time point

Supplementary Figure 8. Forest plot of changes in ketamine-related YMRS scores from baseline to each predefined visit time point

Supplementary Figure 9. Forest plot of changes in ketamine-related VAS-high scores from baseline to each predefined visit time point

Supplementary Figure 10. Forest plot of ketamine-related changes in heart rate from baseline to each predefined visit time point

Supplementary Figure 11. Forest plot of the number of participants with treatment-emergent heart rate abnormalities after esketamine administration

Supplementary Figure 12. Forest plot of ketamine-related changes in respiratory rate at 40 minutes post-dose

Supplementary Figure 13. Forest plot of ketamine-related changes in oximetry at 40 minutes post-dose

Supplementary Figure 14. Forest plot of the number of participants with abnormally low oximetry after esketamine administration

Supplementary Figure 15. Forest plot of the number of participants with different sedation severity after esketamine administration

Supplementary Figure 16. Forest plot of the number of participants with treatment-emergent abnormal laboratory values after esketamine administration

Supplementary Figure 17. Forest plot of changes in ketamine-related BPIC-SS scores from baseline to Week 4

Supplementary Figure 18. Forest plot of the number of participants with nasal symptoms after esketamine administration

Supplementary Figure 19. Forest plot of the number of participants reporting moderate or severe nasal symptoms after esketamine administration

Supplementary Figure 20. Forest plot of the number of participants considered not ready for discharge after esketamine administration

Appendix 2. Search strategies

Supplementary Table 1

The detailed characteristics of studies included in the meta-analysis.

Author	Study characteristics		Patient characteristics		Treatment characteristics			
	Design	Group size (Treatment[a])	Age (years)	Diagnostic Inclusion (Baseline depression severity[d])	Dose (Route)	Frequency	Control	Time frame
**Ketamine**								
Arabzadeh 2018	Parallel (two-arm)	81 (41)	18–60	MDD, DSM-5; Ketamine HDRS: 24.17 ± 2.31; Placebo: 24.62 ± 3.52	50 mg/day (O)	Repeated[b] (Daily)	Placebo	6 weeks
Berman 2000	Crossover (two-arm)	9 (5)	23–56	MDD, DSM-IV; Ketamine HDRS: 33.0 ± 6.7; Placebo: 26.9 ± 5.8	0.5 mg/kg (IV)	Single[c]	Placebo	72 hours
Colla 2024	Parallel (three-arm)	27 (17)	18–70	TRD, ICD-10; Ketamine 160 mg MADRS: 30.4 ± 2.84; 240 mg: 26.2 ± 1.89; Placebo: 29.6 ± 2.16	160 mg/day (O); 240 mg/day O)	Repeated[b] (Daily)	Placebo	2 weeks
Domany 2019	Parallel (two-arm)	40 (22)	18–75	TRD, MINI; Ketamine MADRS: 33.4 ± 5.5; Placebo: 29.99 ± 7.4	1 mg/kg (O)	Repeated[b] (Thrice-weekly)	Placebo	3 weeks
Downey 2016	Parallel (two-arm)	40 (21)	18–45	MDD, DSM-IV; Ketamine MADRS: 23.2 ± 5.65;	0.5 mg/kg (IV)	Single[c]	Placebo	42 days

Author	Study characteristics		Patient characteristics		Treatment characteristics			
	**Design**	**Group size (Treatment[a])**	**Age (years)**	**Diagnostic Inclusion (Baseline depression severity[d])**	**Dose (Route)**	**Frequency**	**Control**	**Time frame**
				Placebo: 20 ± 7.4				
Ekstrand 2022	Parallel (two-arm)	186 (95)	18–85	MDD, DSM-IV; Ketamine MADRS: 33.1 ± 6.3; ECT: 34.5 ± 5.7	0.5 mg/kg (IV)	Repeated[b] (Thrice-weekly)	ECT	Maximum 12 times
Fava 2020	Parallel (five-arm)	99 (80)	18–70	TRD, DSM-IV-TR; Ketamine 0.1 mg/kg MADRS: 33.8 ± 5.9; 0.2 mg/kg: 34.5 ± 8.5; 0.5 mg/kg: 31.6 ± 3.9; 1.0 mg/kg: 32.7 ± 5.9; Midazolam: 33.6 ± 7.1.	0.1, 0.2, 0.5 or 1 mg/kg (IV)	Single[c]	Midazolam	3 days
Gálvez 2018	Parallel (two-arm)	5 (3)	Unclear	MDD, DSM-5; unclear	100 mg (IN)	Repeated[b] (Three times a week for 2 weeks, then weekly for 2 weeks)	Midazolam	4 weeks
Ghasemi 2014	Parallel (two-arm)	18 (9)	18–75	MDD, DSM-IV; Ketamine HDRS: 30.22 ± 5.78; ECT: 35.88±6.47	0.5 mg/kg (IV)	Repeated[b] (Three times a week)	ECT	1 week
Glue 2024	Parallel (five-arm)	168 (131)	18–80	TRD, DSM-5; Ketamine 30mg	30, 60, 120 or 180 mg (O)	Repeated[b] (twice weekly)	Placebo	12 weeks

Author	Study characteristics		Patient characteristics		Treatment characteristics			
	Design	Group size (Treatment[a])	Age (years)	Diagnostic Inclusion (Baseline depression severity[d])	Dose (Route)	Frequency	Control	Time frame
				MADRS: 29.9 ± 4.14; 60mg: 29.3 ± 5.80; 120mg: 31.4 ± 5.19; 180mg: 29.9 ± 4.61; Placebo: 30.2 ± 4.48				
Grunebaum 2018	Parallel (two-arm)	80 (40)	18–65	MDD, DSM-IV; Ketamine 0.5 mg/kg HAM-D: 22.2 ± 4.6; Midazolam: 22.6 ± 3.9	0.5 mg/kg (IV)	Single[c]	Midazolam	1 day
Hu 2016	Parallel (two-arm)	27 (13)	18–60	MDD, DSM-IV; Ketamine MADRS: 36.5 ± 7.8; Placebo:32.3 ± 6.5	0.5 mg/kg (IV)	Single[c]	Placebo	4 weeks
Ionescu 2018	Parallel (two-arm)	26 (13)	18–65	TRD, DSM-IV; Ketamine HDRS: 31.6 ± 5.2; Placebo 26.3 ± 4.8	0.5 mg/kg (IV)	Repeated[b] (Twice weekly)	Placebo	3 weeks
Keilp 2021	Parallel (two-arm)	78 (39)	Unclear	MDD, DSM-5; Ketamine HDRS-24: 29.1 ± 5.9; Midazolam: 30.0 ± 5.9	0.5 mg/kg (IV)	Single[c]	Midazolam	1 day
Kheirabadi 2020	Parallel (three-arm)	39 (27)	18–70	MDD, DSM-5; Oral ketamine HDRS: 21 ± 2.73;	0.5 mg/kg (IM); 1 mg/kg (O)	Repeated[b] (Twice or three times weekly)	ECT	3 weeks

Author	Study characteristics		Patient characteristics		Treatment characteristics			
	Design	Group size (Treatment[a])	Age (years)	Diagnostic Inclusion (Baseline depression severity[d])	Dose (Route)	Frequency	Control	Time frame
Lapidus 2014	Crossover (two-arm)	20 (10)	21–65	IM ketamine: 21 ± 2.9; ECT: 21.83 ± 4.63 MDD, DSM-IV; All participants IDS-C: 42.7 ± 8.5	50 mg (IN)	Single[c]	Placebo	1 week
Li 2016	Parallel (three-arm)	48 (32)	21–65	TRD, DSM-IV-TR; Ketamine 0.2 mg/kg $HDRS_{17}$: 20.9 ± 5.6; 0.5mg/kg: 22.6 ± 5.8; Placebo: 22.8 ± 3.9	0.2 or 0.5 mg/kg (IV)	Single[c]	Placebo	4 hours
Lijffijt 2022	Parallel (four-arm)	33 (20)	≥ 55	TRD, DSM-5; Ketamine 0.1mg/kg MADRS: 35.5 ± 4.93; 0.25mg/kg: 35.80 ± 2.05; 0.5mg/kg: 32.55 ± 2.42; Midazolam: 35.00 ± 5.64	0.1, 0.25 or 0.5 mg/kg (IV)	Single[c]	Midazolam	4 weeks
Loo 2023	Parallel (three-arm)	174 (86)	≥ 18	TRD, DSM-5; Ketamine 0.5 mg/kg MADRS: 30.7 ± 4.8; 0.5–0.9 mg/kg: 28.9 ± 5.7;	Fixed 0.5 mg/kg (subcutaneous); flexible 0.5–0.9 mg/kg (subcutaneous)	Repeated[b] (Twice weekly)	Midazolam	4 weeks

Author	Study characteristics		Patient characteristics		Treatment characteristics			
	Design	Group size (Treatment[a])	Age (years)	Diagnostic Inclusion (Baseline depression severity[d])	Dose (Route)	Frequency	Control	Time frame
				Midazolam: 30.50 ± 5.63				
Murrough 2013	Parallel (two-arm)	73 (48)	21–80	TRD, DSM-IV; Ketamine MADRS: 32.6 ± 6.1; Midazolam: 31.1 ± 5.6	0.5 mg/kg (IV)	Single[c]	Midazolam	1 week
Murrough 2015	Parallel (two-arm)	62 (43)	21–80	TRD, DSM-IV; Ketamine MADRS: 32.5 ± 6.0; Midazolam: 31.0 ± 5.1	0.5 mg/kg (IV)	Single[c]	Midazolam	1 week
Ohtani 2024	Parallel (two-arm)	34 (17)	20–59	TRD, DSM-5; Ketamine MADRS: 29.2 ± 7.9; Placebo: 26.9 ± 7.1	0.5 mg/kg (IV)	Repeated[b] (Twice weekly)	Placebo	2 weeks
Pattanaseri 2024	Parallel (two-arm)	20 (11)	18–64	TRD; Ketamine MADRS: 33.73 ± 7.90; Midazolam:35.33 ± 6.08	0.5 mg/kg (IV)	Repeated[b]	Midazolam	3 days
Price 2014	Parallel (two-arm)	57 (36)	21–80	TRD, DSM-IV; Ketamine MADRS: 33.3 ± 5.6; Midazolam: 32.4 ± 4.8	0.5 mg/kg (IV)	Single[c]	Midazolam	1 week
Seraj 2025	Parallel (two-arm)	80 (40)	18–60	MDD, DSM-5;	3 mg/kg (O)	Single[c]	Midazolam	1 week

Author	Study characteristics		Patient characteristics		Treatment characteristics			
	Design	Group size (Treatment[a])	Age (years)	Diagnostic Inclusion (Baseline depression severity[d])	Dose (Route)	Frequency	Control	Time frame
				Ketamine HAM-D: 25.1 ± 6.4; Midazolam: 22.8 ± 4.6				
Shiroma 2020	Parallel (two-arm)	54 (25)	18–75	TRD, DSM-IV; Ketamine MADRS: 34.88 ± 7.80; Midazolam: 33.82 ± 5.02	0.5 mg/kg (IV)	Repeated[b] (Three times weekly)	Midazolam	2 weeks
Singh 2016 b	Parallel (four-arm)	68 (35)	18–64	TRD, DSM-IV-TR; Ketamine twice weekly MADRS: 33.3 ± 4.9; Three times weekly: 35.4 ± 5.3; Placebo twice weekly: 35.6 ± 3.8; Three times weekly: 36.8 ± 5.8	0.5 mg/kg (IV)	Repeated[b] (two or three times weekly)	Placebo	4 weeks
Sos 2013	Crossover (two-arm)	30 (11)	18–65	MDD, DSM-IV; Ketamine MADRS: 20.4 ± 4.7; Placebo: 24.6 ± 4.8	0.54 mg/kg (IV)	Single[c]	Placebo	1 week

Author	Study characteristics		Patient characteristics		Treatment characteristics			
	Design	Group size (Treatment[a])	Age (years)	Diagnostic Inclusion (Baseline depression severity[d])	Dose (Route)	Frequency	Control	Time frame
Su 2017	Parallel (three-arm)	71 (47)	40–50	TRD, DSM-IV; Ketamine 0.5 mg/kg $HAMD_{17}$: 23.0 ± 4.9; 0.2 mg/kg: 23.1 ± 4.8; Placebo: 23.3 ± 4.1	0.2 or 0.5 mg/kg (IV)	Single[c]	Placebo	1 week
Su 2023	Parallel (two-arm)	84 (42)	20–64	TRD, DSM-5; Ketamine MADRS: 35.83 ± 4.53 Midazolam:38.26±3.83	0.5 mg/kg (IV)	Single[c]	Midazolam	2 weeks
Tiger 2020	Parallel (two-arm)	30 (20)	20–80	MDD, MINI; Ketamine MADRS: 26.3 ± 6.58; Placebo: 30.8 ± 4.92	0.5 mg/kg (IV)	Single[c]	Placebo	1 day
Zarate 2006	Crossover (two-arm)	18 (9)	18–65	TRD, DSM-IV; Ketamine HRSD: 24.89; Placebo: 24.44	0.5 mg/kg (IV)	Single[c]	Placebo	1 week
Zolghadriha 2024	Parallel (two-arm)	64 (32)	25–60	TRD, DSM-5; Ketamine MADRS: 35.16 ± 8.13; Placebo: 32.51 ± 5.66	0.5 mg/kg (IV)	Single[c]	Placebo	8 weeks
**Esketamine**								

Author	Study characteristics		Patient characteristics		Treatment characteristics			
	Design	Group size (Treatment[a])	Age (years)	Diagnostic Inclusion (Baseline depression severity[d])	Dose (Route)	Frequency	Control	Time frame
Canuso 2018	Parallel (two-arm)	66 (35)	19–64	MDD, DSM-IV-TR; Esketamine MADRS: 38.5 ± 6.17; Placebo: 38.8 ± 7.02	84 mg (IN)	Repeated[b] (Twice weekly)	Placebo	4 weeks
Chen 2023	Parallel (two-arm)	252 (124)	18–64	TRD, DSM-5; Esketamine MADRS: 36.5 ± 5.21; Placebo: 35.9 ± 4.50	Flexible 56-84 mg (IN)	Repeated[b] (Twice weekly)	Placebo	4 weeks
Daly 2018	Parallel (four-arm)	67 (34)	20–64	MDD, DSM-IV-TR; Esketamine 28 mg MADRS: 31.3 ± 7.09; 56 mg: 34.9 ± 6.13; 84mg: 30.4 ± 4.67; Placebo: 29.3 ± 5.79	28, 56, or 84 mg (IN)	Repeated[b] (Twice weekly)	Placebo	2 weeks
Daly 2019	Parallel (two-arm)	297 (152)	18–64	TRD, DSM-5; Esketamine MADRS: 40.1 ± 5.56; Placebo: 38.9 ± 4.92	Flexible 56-84 mg (IN)	Repeated[b] (Once weekly or every 2 weeks)	Placebo	32 weeks
Fedgchin 2019	Parallel (three-arm)	346 (233)	18–64	TRD, DSM-5; Esketamine 56 mg MADRS: 37.4 ± 4.76;	56 or 84 mg (IN)	Repeated[b] (Twice weekly)	Placebo	4 weeks

Author	Study characteristics		Patient characteristics		Treatment characteristics			
	Design	Group size (Treatment[a])	Age (years)	Diagnostic Inclusion (Baseline depression severity[d])	Dose (Route)	Frequency	Control	Time frame
				84mg: 37.8 ± 5.58; Placebo: 37.5 ± 6.16				
Fu 2020	Parallel (two-arm)	226 (114)	18–64	MDD, DSM-5; Esketamine MADRS: 41.3 ± 5.87; Placebo: 41.0 ± 6.29	84 mg (IN)	Repeated[b] (Twice weekly)	Placebo	4 weeks
Hong 2025	Parallel (two-arm)	236 (118)	18–65	MDD, DSM-5; Esketamine HDRS-24: 24.2 ± 3.1; Midazolam: 23.9 ± 3.3	0.5 mg/kg (IN)	Repeated[b] (Once weekly)	Placebo	8 weeks
Ionescu 2021	Parallel (two-arm)	230 (115)	18–64	MDD, DSM-5; Esketamine MADRS: 39.5 ± 5.19; Placebo: 39.9 ± 5.76	84 mg (IN)	Repeated[b] (Twice weekly)	Placebo	4 weeks
Ochs-Ross 2020	Parallel (two-arm)	137 (72)	≥ 65	TRD, DSM-5; Esketamine MADRS: 35.5 ± 5.91; Placebo: 34.8 ± 6.44	Flexible 28-84 mg (IN)	Repeated[b] (Twice weekly)	Placebo	4 weeks
Popova 2019	Parallel (two-arm)	227 (116)	18–64	TRD, DSM-5; Esketamine MADRS: 37.0 ± 5.69;	Flexible 56-84 mg (IN)	Repeated[b] (Twice weekly)	Placebo	4 weeks

Author	Study characteristics		Patient characteristics		Treatment characteristics			
	**Design**	**Group size (Treatment[a])**	**Age (years)**	**Diagnostic Inclusion (Baseline depression severity[d])**	**Dose (Route)**	**Frequency**	**Control**	**Time frame**
				Placebo: 37.3 ± 5.66				
Reif 2023	Parallel (two-arm)	676 (336)	18–74	TRD, DSM-5; Esketamine MADRS: 31.4 ± 6.06; Placebo: 31.0 ± 5.83	Flexible 28-84 mg (IN)	Repeated[b] (Twice weekly during weeks 1–4, weekly during weeks 5–8)	Quetiapine	8 weeks
Singh 2016a	Parallel (three-arm)	30 (20)	18–64	TRD, DSM-IV-TR; Esketamine 0.20 mg/kg MADRS: 33.1 ± 3.55; 0.40 mg/kg: 33.7 ± 5.82; Placebo: 33.9 ± 4.15	0.2 or 0.4 mg/kg (IV)	Repeated[b] (Twice weekly)	Placebo	1week
Smith-Apeldoorn 2024	Parallel (two-arm)	111 (57)	18–80	MDD, DSM-5; Esketamine $HDRS_{17}$: 23.2 ± 3.48; Placebo: 23.2 ± 3.49	30mg (O)	Repeated[b] (Three times per day)	Placebo	6 weeks
Takahashi 2021	Parallel (four-arm)	202 (122)	20–64	TRD, DSM-5; Esketamine 28mg MADRS: 38.4 ± 6.07; 56mg: 37.9 ± 5.41; 84mg: 35.9 ± 5.28; Placebo: 37.7 ± 5.65	28, 56, or 84 mg (IN)	Repeated[b] (Twice weekly)	Placebo	4 weeks

[a]Number of participants in the treatment group. [b]Repeated dosing. [c]Single dosing. [d]Baseline depression severity scores are presented by Mean ± SD. DSM-IV-TR, diagnostic and statistical manual of mental disorders, fourth edition-text revision; DSM-5, diagnostic and statistical manual of mental disorders fifth edition; ECT, electroconvulsive therapy; HAM-D, Hamilton rating scale for depression; $HDRS_{17}$, 17-item Hamilton depression rating scale; HDRS-24, 24-item Hamilton depression rating scale; ICD-10, the 10th revision of the international classification of diseases; IDS-C, inventory of depressive symptoms–clinician rated; IM, intramuscular; IN, intranasal; IV, intravenous; MADRS, Montgomery-Åsberg depression rating scale; MDD, major depressive disorder; MINI, Mini international neuropsychiatric interview; N, number of participants; O, oral; TRD, treatment-resistant depression.

Supplementary Table 2

Reported measures of safety outcomes in included trials.

Study	Primary Safety Outcomes				Secondary Safety Outcomes			
	Dropouts due to AEs	Number of participants with at least one AE	Number of participants with at least one serious AE	Number of participants with the specific AE	Psychiatric or Psychotomimetic side-effects	Cardiovascular side-effects	Neurological or Cognitive side-effects	Other side-effects
**Ketamine**								
Arabzadeh 2018	N/A	N/A	N/A	Reported	N/A	N/A	N/A	N/A
Berman 2000	N/A	N/A	N/A	N/A	BPRS, VAS-high	N/A	N/A	N/A
Colla 2024	N/A	N/A	N/A	Reported	DSS-4	BP, HR	N/A	Laboratory tests
Domany 2019	Reported	N/A	N/A	Reported	N/A	BP, HR, oximetry	N/A	N/A
Downey 2016	N/A	N/A	N/A	N/A	CADSS	N/A	N/A	N/A
Ekstrand 2022	Reported	Reported	Reported	Reported	CADSS, BPRS	N/A	N/A	N/A
Fava 2020	N/A	Reported	Reported	Reported	CADSS	BP, HR, ECG	N/A	Laboratory tests, CBC
Gálvez 2018	N/A	Reported	N/A	Reported	CADSS, BPRS, YMRS	BP, HR	Orientation, CogState computerized battery	LFTs, BPIC-SS
Ghasemi 2014	N/A	N/A	N/A	Reported	N/A	BP, HR, oximetry	N/A	N/A
Glue 2024	N/A	N/A	Reported	Reported	BPRS+, CADSS	BP, ECG	MoCA, Verbal Fluency tests	BPIC-SS, Laboratory tests
Grunebaum 2018	N/A	N/A	N/A	Reported	CADSS, BPRS	BP, RR, oximetry	N/A	N/A
Hu 2016	N/A	Reported	Reported	Reported	CADSS, BPRS, YMRS	BP, HR, ECG, RR, oximetry	N/A	N/A

Study	Primary Safety Outcomes				Secondary Safety Outcomes			
	Dropouts due to AEs	Number of participants with at least one AE	Number of participants with at least one serious AE	Number of participants with the specific AE	Psychiatric or Psychotomimetic side-effects	Cardiovascular side-effects	Neurological or Cognitive side-effects	Other side-effects
Ionescu 2018	Reported	N/A	N/A	Reported	CADSS	BP, HR, oximetry	N/A	N/A
Keilp 2021	N/A	N/A	N/A	N/A	N/A	BP, HR, RR, oximetry	Neuropsycholigical measures	N/A
Kheirabadi 2020	N/A	N/A	N/A	Reported	N/A	HR, RR, BP, oximetry	N/A	N/A
Lapidus 2014	N/A	N/A	N/A	N/A	CADSS, BPRS+, YMRS	BP, HR	N/A	N/A
Li 2016	N/A	N/A	N/A	Reported	BPRS+	N/A	N/A	N/A
Lijffijt 2022	N/A	Reported	Reported	Reported	CADSS, BPRS	BP, HR	N/A	N/A
Loo 2023	Reported	N/A	Reported	Reported	CADSS, BPRS+, YMRS	BP, HR	CogState computerized battery	BPIC-SS, Liver function tests, Craving & Recreational Use Questionnaire
Murrough 2013	N/A	N/A	Reported	Reported	CADSS, BPRS+, YMRS	BP, HR, ECG oximetry	N/A	N/A
Murrough 2015	N/A	N/A	N/A	N/A	N/A	N/A	MCCB	N/A
Ohtani 2024	N/A	Reported	N/A	Reported	N/A	BP	N/A	N/A
Pattanaseri 2024	N/A	N/A	N/A	Reported	Dissociative Experiences Measure (Oxford)	BP, HR	N/A	N/A

Study	Primary Safety Outcomes				Secondary Safety Outcomes			
	Dropouts due to AEs	Number of participants with at least one AE	Number of participants with at least one serious AE	Number of participants with the specific AE	Psychiatric or Psychotomimetic side-effects	Cardiovascular side-effects	Neurological or Cognitive side-effects	Other side-effects
Price 2014	N/A	N/A	N/A	N/A	N/A	N/A	N/A	N/A
Seraj 2025	N/A	Reported	N/A	Reported	N/A	N/A	N/A	N/A
Shiroma 2020	N/A	N/A	Reported	Reported	CADSS, BPRS, YMRS	HR, BP, oximetry, RR	Cognitive performance (CogState)	N/A
Singh 2016b	Reported	Reported	Reported	Reported	CADSS, BPRS+	BP, HR, ECG, oximetry	N/A	Laboratory tests
Sos 2013	N/A	N/A	N/A	Reported	BPRS	N/A	N/A	N/A
Su 2017	N/A	N/A	N/A	N/A	BPRS	BP, HR, oximetry	N/A	N/A
Su 2023	N/A	N/A	Reported	Reported	CADSS	N/A	N/A	N/A
Tiger 2020	N/A	N/A	N/A	Reported	N/A	BP, HR	N/A	N/A
Zarate 2006	N/A	N/A	N/A	N/A	BPRS+, YMRS	N/A	N/A	N/A
Zolghadriha 2024	N/A	N/A	N/A	Reported	N/A	BP, HR, oximetry	N/A	N/A
**Esketamine**								
Canuso 2018	Reported	Reported	Reported	Reported	CADSS	BP	N/A	N/A
Chen 2023	Reported	Reported	Reported	Reported	CADSS	BP, HR, ECG, oximetry, RR	N/A	PWC-20, laboratory tests, nasal examination,
Daly 2018	Reported	Reported	N/A	Reported	CADSS, BPRS	BP, HR	N/A	Laboratory tests

Study	Primary Safety Outcomes				Secondary Safety Outcomes			
	Dropouts due to AEs	Number of participants with at least one AE	Number of participants with at least one serious AE	Number of participants with the specific AE	Psychiatric or Psychotomimetic side-effects	Cardiovascular side-effects	Neurological or Cognitive side-effects	Other side-effects
Daly 2019	Reported	Reported	Reported	Reported	CADSS, BPRS	BP, ECG	Computerized cognitive battery, HVLT-R	Laboratory tests, PWC-20
Fedgchin 2019	Reported	Reported	Reported	Reported	CADSS, BPRS	ECG, BP	Cognitive testing, MOAA/S	Laboratory tests, PWC-20, CGADR, nasal examination, nasal symptom questionnaire
Fu 2020	Reported	Reported	Reported	Reported	CADSS	BP, HR, ECG, oximetry	MOAA/S	Laboratory tests, nasal examination
Hong 2025	Reported	N/A	N/A	Reported	CADSS, BPRS+	HR, BP, oximetry	MoCA, MOAA/S	N/A
Ionescu 2021	Reported	Reported	Reported	Reported	CADSS	HR, BP, ECG, oximetry	MOAA/S	Laboratory tests, nasal examination
Ochs-Ross 2020	Reported	Reported	Reported	Reported	CADSS, BPRS	BP, oximetry, RR, ECG	Computerized cognitive battery, HVLT-R, MOAA/S	PWC-20, CGADR, Laboratory tests, BPIC-SS
Popova 2019	Reported	Reported	Reported	Reported	CADSS, BPRS	BP, ECG	Cognitive testing, MOAA/S	PWC-20, CGADR, Laboratory tests, BPIC-SS, nasal examination, nasal symptom questionnaire,

Study	Primary Safety Outcomes				Secondary Safety Outcomes			
	Dropouts due to AEs	Number of participants with at least one AE	Number of participants with at least one serious AE	Number of participants with the specific AE	Psychiatric or Psychotomimetic side-effects	Cardiovascular side-effects	Neurological or Cognitive side-effects	Other side-effects
								abuse or addiction
Reif 2023	Reported	Reported	Reported	Reported	N/A	N/A	N/A	N/A
Singh 2016a	Reported	Reported	Reported	Reported	CADSS, BPRS	HR, BP, ECG, oximetry, RR	MGH-CPFQ	Laboratory tests,
Smith-Apeldoorn 2024	Reported	N/A	Reported	Reported	DSS, QPE, ISDI	BP	N/A	Liver enzyme levels
Takahashi 2021	Reported	Reported	Reported	Reported	CADSS, BPRS	BP, ECG	MOAA/S	Laboratory tests, PWC-20, abuse or addiction

N/A: not available. AEs, adverse events; BP, blood pressure; BPIC-SS, bladder pain-interstitial cystitis symptoms scale; BPRS, brief psychiatric rating scale; BPRS+, brief psychiatric rating scale positive symptom subscale; CADSS, clinician administered dissociative states scale; CBC, complete blood count; CGADR, clinical global assessment of discharge readiness; DSS-4, dissociative symptom scale-4; DSS, dissociation tension scale; ECG, electrocardiography; HR, heart rate; HVLT-R, Hopkins verbal learning test-revised; ISDI, Iowa sleep disturbance inventory; LFTs, liver function tests; MCCB, MATRICS consensus cognitive battery; MGH-CPFQ, Massachusetts General Hospital-cognitive and physical functioning questionnaire; MOAA/S, modified observer's assessment of alertness/sedation; MoCA, Montreal cognitive assessment; PWC-20, physician withdrawal checklist; QPE, questionnaire for psychotic experiences; RR, respiratory rate; VAS-high, visual analogue scales score for intoxication "high"; YMRS, Young mania rating scale.

Supplementary Table 3

Combined effect of ketamine after excluding each individual study in turn.

**Outcome**	**Culled references**	**Risk Ratio (M-H, Random, 95% CI)**	***p*[a] value**	***I*²/%**	***p*[b] value**
Number of participants with at least one AE	Ekstrand 2022	1.59 [0.91, 2.76]	0.10	91	< 0.00001
	Fava 2020	1.41 [1.02, 1.95]	0.04	91	< 0.00001
	Gálvez 2018	1.42 [1.03, 1.96]	0.03	91	< 0.00001
	Hu 2016	1.28 [0.96, 1.69]	0.09	88	< 0.00001
	Lijffijt 2022	1.57 [0.94, 2.64]	0.09	90	< 0.00001
	Ohtani 2024	1.20 [0.95, 1.51]	0.12	81	< 0.0001
	Seraj 2025	1.31 [0.97, 1.76]	0.08	89	< 0.00001
	Singh 2016 b	1.33 [0.91, 1.81]	0.07	89	< 0.00001

[a] This *p* value is the statistical value for overall effect. [b] This *p* value is the statistical value for heterogeneity. AE, adverse event.

Supplementary Table 4

Meta-analyses for each specific AE associated with ketamine.

SOC	AEs	Number of studies	Number of participants	Risk Ratio (95% CI)	*p* value
Psychiatric disorders	Abnormal dreams	1	99	0.24 [0.02, 3.63]	0.30
	**Affect lability**	**1**	**181**	**4.29 [1.26, 14.53]**	**0.02**
	Agitation	3	102	1.12 [0.18, 6.78]	0.91
	**Anxiety**	**12**	**962**	**2.06 [1.41, 3.01]**	**0.0002**
	Blunted affect	1	30	15.00 [0.88, 254.88]	0.06
	Bruxism	1	27	1.83 [0.08, 41.17]	0.70
	Confusional state	3	385	3.12 [0.55, 17.65]	0.20
	**Depersonalisation/ Derealisation disorder**	**3**	**338**	**4.58 [2.35, 8.92]**	**< 0.00001**
	Depression	3	334	1.48 [0.45, 4.89]	0.52
	Disorientation	1	67	0.31 [0.01, 7.24]	0.46
	**Dissociation**	**12**	**987**	**3.91 [2.89, 5.28]**	**< 0.00001**
	Dissociative disorder	2	131	14.55 [0.49, 428.06]	0.12
	**Emotional disorder**	**1**	**80**	**17.00 [1.01, 284.96]**	**0.05**
	**Euphoric mood**	**6**	**526**	**9.69 [3.24, 28.92]**	**< 0.0001**
	Hallucination	2	56	3.84 [0.47, 31.19]	0.21
	Hallucination, tactile	1	67	2.75 [0.12, 65.18]	0.53
	Hallucination, visual	2	131	4.79 [0.57, 39.90]	0.15
	Initial insomnia	1	99	0.08 [0.00, 1.95]	0.12
	Insomnia	4	253	1.53 [0.66, 3.57]	0.33
	Intentional self-injury	1	99	0.08 [0.00, 1.95]	0.12
	libido decreased	2	38	1.62 [0.27, 9.62]	0.60
	Irritability	2	147	0.96 [0.10, 9.01]	0.97
	**Moodiness**	**1**	**174**	**10.74 [2.60, 44.43]**	**0.001**
	Nervousness	1	81	0.65 [0.11, 3.69]	0.63
	Nightmare	2	94	2.98 [0.32, 27.43]	0.34
	Paranoia	2	248	1.24 [0.14, 10.85]	0.84
	Poor quality sleep	2	166	1.80 [0.20, 15.89]	0.60
	**Restlessness**	**8**	**513**	**1.50 [1.08, 2.09]**	**0.02**
	Sleep Disorder	1	181	4.95 [0.24, 101.60]	0.30
	Suicidal ideation	1	99	1.23 [0.06, 24.71]	0.89
	Suicide attempt	2	248	0.80 [0.25, 2.51]	0.70
Nervous system disorders	**Amnesia**	**2**	**248**	**0.18 [0.09, 0.35]**	**< 0.00001**
	Ataxia	2	105	1.07 [0.22, 5.26]	0.93
	**Autonomic nervous system imbalance**	**1**	**80**	**3.33 [1.01, 11.22]**	**0.05**
	Cognitive disorder	1	67	2.75 [0.12, 65.18]	0.53
	Disturbance in attention	4	164	1.30 [0.39, 4.28]	0.67
	**Dizziness**	**17**	**1210**	**2.40 [1.60, 3.59]**	**< 0.0001**

SOC	AEs	Number of studies	Number of participants	Risk Ratio (95% CI)	*p* value
	Dizziness postural	3	172	0.71 [0.22, 2.25]	0.56
	Dysarthria	1	67	2.75 [0.12, 65.18]	0.53
	**Dysgeusia**	**2**	**241**	**2.34 [1.32, 4.15]**	**0.004**
	Headache	15	1159	1.22 [0.62, 2.38]	0.57
	Hypertonia	1	181	0.25 [0.03, 2.17]	0.21
	**Hypoaesthesia**	**2**	**101**	**8.42 [1.10, 64.21]**	**0.04**
	Loss of consciousness	3	206	0.37 [0.06, 2.26]	0.28
	Memory impairment	2	119	0.25 [0.00, 29.94]	0.57
	Migraine	1	67	0.31 [0.01, 7.24]	0.46
	**Movement Disorder**	**1**	**174**	**4.09 [1.61, 10.41]**	**0.003**
	Myoclonus	1	181	0.33 [0.01, 7.99]	0.50
	Nystagmus	1	39	2.32 [0.12, 44.99]	0.58
	**Paraesthesia**	**4**	**502**	**3.25 [1.11, 9.46]**	**0.03**
	**Paralysis**	**2**	**254**	**5.35 [3.08, 9.29]**	**< 0.00001**
	Presyncope	1	99	0.74 [0.03, 17.51]	0.85
	Sedation	3	321	1.22 [0.68, 2.20]	0.51
	Seizure	1	181	0.20 [0.01, 4.06]	0.29
	Sensory disturbance	1	27	1.83 [0.08, 41.17]	0.70
	Sleep deficit	1	5	0.67 [0.08, 5.54]	0.71
	Somnolence	8	472	1.49 [0.66, 3.39]	0.34
	Tremor	4	240	1.44 [0.59, 3.51]	0.43
Gastrointestinal disorders	Abdominal discomfort	1	67	2.75 [0.12, 65.18]	0.53
	Abdominal pain	2	262	0.44 [0.10, 1.96]	0.28
	Abdominal pain upper	1	67	0.31 [0.01, 7.24]	0.46
	**Constipation**	**6**	**487**	**2.70 [1.00, 7.32]**	**0.05**
	Diarrhoea	7	559	1.16 [0.62, 2.15]	0.65
	Dry mouth	8	626	1.61 [0.73, 3.56]	0.24
	Dyspepsia	2	166	1.80 [0.20, 15.89]	0.60
	Dysphagia	1	80	3.00 [0.13, 71.51]	0.50
	Hypoaesthesia Oral	1	67	4.58 [0.23, 92.00]	0.32
	**Nausea**	**19**	**1455**	**1.61 [1.08, 2.39]**	**0.02**
	Paraesthesia oral	1	67	2.75 [0.12, 65.18]	0.53
	**Salivary hypersecretion**	**3**	**382**	**16.42 [4.66, 57.88]**	**< 0.0001**
	Throat irritation	1	181	0.20 [0.02, 1.66]	0.14
	Vomiting	10	736	1.38 [0.72, 2.65]	0.33
General disorders and administration site conditions	Asthenia	1	99	0.74 [0.03, 17.51]	0.85
	**Chest discomfort**	**1**	**174**	**4.30 [1.70, 10.88]**	**0.002**
	Chest pain	1	72	1.06 [0.21, 5.41]	0.94
	Crying	2	132	3.19 [0.23, 44.77]	0.39
	Decreased energy	3	159	1.19 [0.68, 2.11]	0.54
	Fatigue	10	861	1.09 [0.63, 1.88]	0.76

SOC	AEs	Number of studies	Number of participants	Risk Ratio (95% CI)	*p* value
	Feeling abnormal	2	235	1.91 [0.51, 7.11]	0.33
	Feeling cold	3	321	1.29 [0.31, 5.36]	0.72
	Feeling drunk	1	34	1.00 [0.07, 14.72]	1.00
	**Feeling hot**	**1**	**174**	**2.22 [1.20, 4.11]**	**0.01**
	Inflammation	1	67	2.75 [0.12, 65.18]	0.53
	Infusion site pain	1	67	4.58 [0.23, 92.00]	0.32
	Injection site extravasation	1	67	0.31 [0.01, 7.24]	0.46
	Malaise	6	297	0.99 [0.43, 2.26]	0.97
	swelling	1	181	0.49 [0.05, 5.36]	0.56
Ear and labyrinth disorders	Hyperacusis	1	67	2.75 [0.12, 65.18]	0.53
	Tinnitus	6	519	1.62 [0.72, 3.63]	0.25
	**Vertigo**	**3**	**454**	**3.01 [1.91, 4.73]**	**< 0.00001**
Eye disorders	Altered visual depth perception	1	67	2.75 [0.12, 65.18]	0.53
	**Diplopia**	**2**	**248**	**10.61 [2.94, 38.32]**	**0.0003**
	**Vision blurred**	**10**	**763**	**4.12 [2.58, 6.58]**	**< 0.00001**
Skin and subcutaneous tissue disorders	Acne	1	67	0.31 [0.01, 7.24]	0.46
	Dermatitis	1	99	0.08 [0.00, 1.95]	0.12
	Dermatitis contact	2	166	0.48 [0.05, 4.46]	0.52
	Dry skin	2	105	1.28 [0.58, 2.87]	0.54
	**Hyperhidrosis**	**4**	**333**	**2.29 [1.33, 3.95]**	**0.003**
	Pruritus	5	231	2.11 [0.75, 5.90]	0.16
	Rash	3	172	1.68 [0.37, 7.60]	0.50
	Skin irritation	1	181	2.97 [0.31, 27.99]	0.34
	Urticaria	1	67	2.75 [0.12, 65.18]	0.53
Investigations	Blood bilirubin increased	1	27	0.20 [0.01, 4.57]	0.32
	Blood Potassium Decreased	1	67	2.75 [0.12, 65.18]	0.53
	Blood pressure increased	5	284	3.15 [0.96, 10.38]	0.06
	Blood Pressure Systolic Increased	1	18	7.00 [0.41, 118.69]	0.18
	Blood testosterone abnormal	1	99	0.74 [0.03, 17.51]	0.85
	Drug screen positive	1	67	0.31 [0.01, 7.24]	0.46
	Electrocardiogram St-T change	1	67	2.75 [0.12, 65.18]	0.53
	Heart rate increased	2	98	4.81 [0.58, 39.72]	0.15
	Hepatic enzyme increased	2	126	1.58 [0.18, 13.65]	0.68
	**Oxygen saturation decreased**	**1**	**181**	**0.33 [0.11, 0.98]**	**0.05**
	Weight decreased	1	67	0.31 [0.01, 7.24]	0.46

SOC	AEs	Number of studies	Number of participants	Risk Ratio (95% CI)	*p* value
	White blood cell count decreased	1	99	0.74 [0.03, 17.51]	0.85
Musculoskeletal disorders	Arthralgia	1	99	0.08 [0.00, 1.95]	0.12
	Back pain	1	99	0.24 [0.02, 3.63]	0.30
	Bone pain	1	67	2.75 [0.12, 65.18]	0.53
	Intervertebral disc degeneration	1	67	0.31 [0.01, 7.24]	0.46
	Musculoskeletal pain	2	119	0.71 [0.09, 5.79]	0.75
	**Myalgia**	**1**	**181**	**0.23 [0.15, 0.35]**	**< 0.00001**
	Pain in extremity	1	99	1.23 [0.06, 24.71]	0.89
Cardiac disorders	Bradycardia	1	181	0.08 [0.00, 1.33]	0.08
	Cardiac flutter	1	67	0.31 [0.01, 7.24]	0.46
	Palpitations	5	373	1.80 [0.84, 3.86]	0.13
	Tachycardia	2	280	0.30 [0.02, 4.94]	0.40
	Ventricular extrasystoles	1	67	0.31 [0.01, 7.24]	0.46
Vascular disorders	Flushing	1	99	0.74 [0.03, 17.51]	0.85
	Hot flush	1	99	0.74 [0.03, 17.51]	0.85
	Hypertension	2	166	2.37 [0.27, 20.47]	0.43
	Hypotension	1	181	0.33 [0.01, 7.99]	0.50
	Orthostatic hypotension	1	67	2.75 [0.12, 65.18]	0.53
	Thrombosis	1	181	0.49 [0.05, 5.36]	0.56
Renal and urinary disorders	Dysuria	3	193	0.93 [0.14, 6.10]	0.94
	Micturition urgency	1	67	2.83 [0.11, 71.89]	0.53
	Pollakiuria	2	105	3.03 [0.47, 19.38]	0.24
	Urinary retention	1	181	3.00 [0.12, 74.62]	0.50
Infections and infestations	Gastroenteritis viral	1	99	0.08 [0.00, 1.95]	0.12
	Groin abscess	1	67	2.75 [0.12, 65.18]	0.53
	Infection	1	181	2.97 [0.12, 71.89]	0.50
	Pharyngitis	1	67	2.75 [0.12, 65.18]	0.53
	Respiratory tract infection	1	99	0.74 [0.03, 17.51]	0.85
	Sinusitis	1	99	0.08 [0.00, 1.95]	0.12
	Tooth abscess	1	99	0.74 [0.03, 17.51]	0.85
	Upper respiratory tract infection	2	267	0.71 [0.25, 2.01]	0.52
	Urinary tract infection	1	67	0.91 [0.06, 14.02]	0.95
	Viral upper respiratory tract infection	1	99	0.74 [0.03, 17.51]	0.85
Injury, poisoning and procedural complications	Animal bite	1	67	2.75 [0.12, 65.18]	0.53
	Exposure to toxic agent	1	99	0.74 [0.03, 17.51]	0.85
	Fall	1	99	0.74 [0.03, 17.51]	0.85
	Ligament sprain	1	67	0.31 [0.01, 7.24]	0.46

SOC	AEs	Number of studies	Number of participants	Risk Ratio (95% CI)	*p* value
	Overdose	1	99	0.74 [0.03, 17.51]	0.85
	Wound	1	67	2.75 [0.12, 65.18]	0.53
Respiratory, thoracic and mediastinal disorders	Asthma	1	67	0.31 [0.01, 7.24]	0.46
	Bronchospasm	1	181	0.09 [0.01, 1.60]	0.10
	Cough	1	99	0.74 [0.03, 17.51]	0.85
	**Dyspnoea**	**2**	**241**	**3.49 [1.26, 9.61]**	**0.02**
	Laryngospasm	1	181	0.09 [0.01, 1.60]	0.10
	Nasal congestion	2	166	0.26 [0.03, 2.09]	0.21
	Respiratory distress	1	67	2.75 [0.12, 65.18]	0.53
Metabolism and nutrition disorders	Decreased appetite	1	81	0.98 [0.14, 6.59]	0.98
	Fluid retention	1	67	0.31 [0.01, 7.24]	0.46
	Increased appetite	1	99	0.74 [0.03, 17.51]	0.85
Reproductive system and breast disorders	Sexual dysfunction	1	54	1.16 [0.18, 7.64]	0.88

AEs, adverse events; SOC, system organ class.

Supplementary Table 5

Subgroup analyses by comparators for each specific AE associated with ketamine.

Comparator interventions	SOC	AEs	Number of studies	Number of participants	Risk Ratio (M-H, Random, 95% CI)	*p* value
**Placebo**						
	Psychiatric disorders	Agitation	2	97	1.58 [0.07, 34.17]	0.77
		**Anxiety**	**5**	**363**	**4.37 [1.60, 11.96]**	**0.004**
		Blunted affect	1	30	15.00 [0.88, 254.88]	0.06
		Bruxism	1	27	1.83 [0.08, 41.17]	0.70
		Confusional state	1	30	11.67 [0.66, 206.89]	0.09
		Depression	2	235	1.35 [0.31, 5.90]	0.69
		Disorientation	1	67	0.31 [0.01, 7.24]	0.46
		**Dissociation**	**7**	**414**	**9.02 [3.05, 26.69]**	**< 0.0001**
		Dissociative disorder	2	131	14.55 [0.49, 428.06]	0.12
		**Euphoric mood**	**4**	**171**	**6.71 [1.56, 28.95]**	**0.01**
		Hallucination	2	56	3.84 [0.47, 31.19]	0.21
		Hallucination, tactile	1	67	2.75 [0.12, 65.18]	0.53
		Hallucination, visual	2	131	4.79 [0.57, 39.90]	0.15
		Insomnia	1	67	0.18 [0.01, 3.68]	0.27
		Irritability	1	67	2.75 [0.12, 65.18]	0.53
		Nervousness	1	81	0.65 [0.11, 3.69]	0.63
		Nightmare	2	94	2.98 [0.32, 27.43]	0.34
		Paranoia	1	67	4.58 [0.23, 92.00]	0.32
		Poor quality sleep	1	67	2.75 [0.12, 65.18]	0.53
		Restlessness	3	175	2.16 [0.56, 8.34]	0.27
		Suicide attempt	1	67	2.75 [0.12, 65.18]	0.53
	Nervous system disorders	Amnesia	1	67	0.31 [0.01, 7.24]	0.46
		Cognitive disorder	1	67	2.75 [0.12, 65.18]	0.53
		**Dizziness**	**8**	**529**	**3.48 [1.55, 7.80]**	**0.002**
		Dizziness Postural	1	67	0.31 [0.01, 7.24]	0.46
		Dysarthria	1	67	2.75 [0.12, 65.18]	0.53
		Dysgeusia	1	67	4.58 [0.23, 92.00]	0.32
		**Headache**	**7**	**427**	**3.16 [1.01, 9.88]**	**0.05**
		Loss of consciousness	1	27	0.20 [0.01, 4.57]	0.32
		**Hypoaesthesia**	**2**	**101**	**8.42 [1.10, 64.21]**	**0.04**
		Migraine	1	67	0.31 [0.01, 7.24]	0.46
		Paraesthesia	1	67	6.42 [0.34, 119.61]	0.21
		Sedation	1	67	4.58 [0.23, 92.00]	0.32
		Sensory disturbance	1	27	1.83 [0.08, 41.17]	0.70

**Comparator interventions**	**SOC**	**AEs**	**Number of studies**	**Number of participants**	**Risk Ratio (M-H, Random, 95% CI)**	***p* value**
		Somnolence	3	141	3.10 [0.65, 14.82]	0.16
		Tremor	1	81	1.30 [0.31, 5.45]	0.72
	Gastrointestinal disorders	Abdominal dscomfort	1	67	2.75 [0.12, 65.18]	0.53
		Abdominal pain	1	81	0.65 [0.11, 3.69]	0.63
		Abdominal pain upper	1	67	0.31 [0.01, 7.24]	0.46
		Constipation	1	67	2.75 [0.12, 65.18]	0.53
		Diarrhoea	2	94	2.24 [0.24, 20.58]	0.48
		Dry mouth	1	27	0.36 [0.02, 8.06]	0.52
		Dyspepsia	1	67	2.75 [0.12, 65.18]	0.53
		Hypoaesthesia oral	1	67	4.58 [0.23, 92.00]	0.32
		Nausea	9	559	1.82 [0.92, 3.61]	0.08
		Paraesthesia oral	1	67	2.75 [0.12, 65.18]	0.53
		Salivary hypersecretion	1	27	11.79 [0.72, 194.19]	0.08
		Vomiting	3	137	1.29 [0.22, 7.65]	0.78
	General disorders	Crying	1	48	1.00 [0.10, 10.22]	1.00
		Fatigue	3	262	0.80 [0.26, 2.43]	0.69
		Feeling abnormal	2	235	1.91 [0.51, 7.11]	0.33
		Feeling cold	1	67	2.75 [0.12, 65.18]	0.53
		Feeling drunk	1	34	1.00 [0.07, 14.72]	1.00
		Inflammation	1	67	2.75 [0.12, 65.18]	0.53
		Infusion site pain	1	67	4.58 [0.23, 92.00]	0.32
		Injection site extravasation	1	67	0.31 [0.01, 7.24]	0.46
	Ear and labyrinth disorders	Hyperacusis	1	67	2.75 [0.12, 65.18]	0.53
	Eye disorders	Altered visual depth perception	1	67	2.75 [0.12, 65.18]	0.53
		Diplopia	1	67	2.75 [0.12, 65.18]	0.53
		**Vision blurred**	**5**	**249**	**3.45 [0.98, 12.13]**	**0.05**
	Skin disorders	Acne	1	67	0.31 [0.01, 7.24]	0.46
		Dermatitis contact	1	67	0.31 [0.01, 7.24]	0.46
		Pruritus	1	67	2.75 [0.12, 65.18]	0.53
		Rash	1	67	0.31 [0.01, 7.24]	0.46
		Urticaria	1	67	2.75 [0.12, 65.18]	0.53
	Investigations	Blood bilirubin increased	1	27	0.20 [0.01, 4.57]	0.32
		Blood potassium decreased	1	67	2.75 [0.12, 65.18]	0.53

Comparator interventions	SOC	AEs	Number of studies	Number of participants	Risk Ratio (M-H, Random, 95% CI)	*p* value
		**Blood pressure increased**	**3**	**131**	**10.44 [1.98, 54.95]**	**0.006**
		Drug screen positive	1	67	0.31 [0.01, 7.24]	0.46
		Electrocardiogram St-T change	1	67	2.75 [0.12, 65.18]	0.53
		Hepatic enzyme increased	1	27	3.06 [0.16, 57.93]	0.46
		Weight decreased	1	67	0.31 [0.01, 7.24]	0.46
	Musculoskeletal and connective tissue disorders	Bone pain	1	67	2.75 [0.12, 65.18]	0.53
		Intervertebral disc degeneration	1	67	0.31 [0.01, 7.24]	0.46
	Cardiac disorders	Cardiac flutter	1	67	0.31 [0.01, 7.24]	0.46
		Palpitations	2	94	0.99 [0.15, 6.70]	1.00
		Ventricular extrasystoles	1	67	0.31 [0.01, 7.24]	0.46
	Vascular disorders	Hypertension	1	67	6.42 [0.34, 119.61]	0.21
		Orthostatic hypotension	1	67	2.75 [0.12, 65.18]	0.53
	Renal and urinary disorders	Dysuria	1	67	0.31 [0.01, 7.24]	0.46
		Micturition urgency	1	67	2.75 [0.12, 65.18]	0.53
	Infections and infestations	Groin abscess	1	67	2.75 [0.12, 65.18]	0.53
		Pharyngitis	1	67	2.75 [0.12, 65.18]	0.53
		Upper respiratory tract infection	1	168	0.71 [0.23, 2.12]	0.54
		Urinary tract infection	1	67	0.91 [0.06, 14.02]	0.95
	Injury, poisoning and procedural complications	Animal bite	1	67	2.75 [0.12, 65.18]	0.53
		Ligament sprain	1	67	0.31 [0.01, 7.24]	0.46
		Wound	1	67	2.75 [0.12, 65.18]	0.53
	Respiratory, thoracic and mediastinal disorders	Asthma	1	67	0.31 [0.01, 7.24]	0.46
		Dyspnoea	1	67	2.75 [0.12, 65.18]	0.53
		Nasal congestion	1	67	0.31 [0.01, 7.24]	0.46
		Respiratory distress	1	67	2.75 [0.12, 65.18]	0.53
	Metabolism and nutrition disorders	Decreased appetite	1	81	0.98 [0.14, 6.59]	0.98
		Fluid retention	1	67	0.31 [0.01, 7.24]	0.46
**Midazolam**						
	Psychiatric disorders	Abnormal dreams	1	99	0.24 [0.02, 3.63]	0.30
		Agitation	1	5	0.67 [0.08, 5.54]	0.71
		Anxiety	6	418	1.49 [0.81, 2.74]	0.20

Comparator interventions	SOC	AEs	Number of studies	Number of participants	Risk Ratio (M-H, Random, 95% CI)	*p* value
		**Autonomic nervous system imbalance**	**1**	**80**	**3.33 [0.99, 11.22]**	**0.05**
		**Confusional state**	**1**	**174**	**6.01 [3.02, 11.96]**	**< 0.00001**
		**Depersonalisation/ Derealisation disorder**	**3**	**338**	**4.58 [2.35, 8.92]**	**< 0.00001**
		Depression	1	99	1.73 [0.09, 32.13]	0.71
		Dissociation	3	353	3.99 [0.96, 16.62]	0.06
		**Emotional disorder**	**1**	**80**	**17.00 [1.01, 284.96]**	**0.05**
		**Euphoric mood**	**1**	**174**	**9.21 [1.19, 71.14]**	**0.03**
		Initial insomnia	1	99	0.08 [0.00, 1.95]	0.12
		Insomnia	3	186	1.84 [0.76, 4.44]	0.18
		Intentional self-injury	1	99	0.08 [0.00, 1.95]	0.12
		Irritability	1	80	0.33 [0.01, 7.95]	0.50
		libido decreased	2	38	1.62 [0.27, 9.62]	0.60
		**Moodiness**	**1**	**174**	**10.74 [2.60, 44.43]**	**0.001**
		Poor quality sleep	1	99	1.23 [0.06, 24.71]	0.89
		Restlessness	5	338	1.46 [0.90, 2.36]	0.13
		Suicidal ideation	1	99	1.23 [0.06, 24.71]	0.89
	Nervous system disorders	Ataxia	2	105	1.07 [0.22, 5.26]	0.93
		Disturbance in attention	4	164	1.30 [0.39, 4.28]	0.67
		**Dizziness**	**9**	**681**	**2.07 [1.30, 3.31]**	**0.002**
		Dizziness postural	2	105	0.69 [0.14, 3.44]	0.65
		**Dysgeusia**	**1**	**174**	**2.28 [1.27, 4.09]**	**0.006**
		Headache	6	512	1.12 [0.72, 1.74]	0.62
		Loss of consciousness	2	179	0.50 [0.05, 4.67]	0.54
		Memory impairment	1	80	3.00 [0.13, 71.51]	0.50
		**Movement Disorder**	**1**	**174**	**4.09 [1.61, 10.41]**	**0.003**
		**Paraesthesia**	**2**	**254**	**4.81 [1.06, 21.85]**	**0.04**
		**Paralysis**	**2**	**254**	**5.35 [3.08, 9.29]**	**< 0.00001**
		Presyncope	1	99	0.74 [0.03, 17.51]	0.85
		Sedation	2	254	1.15 [0.66, 2.00]	0.61
		Sleep deficit	1	5	0.67 [0.08, 5.54]	0.71
		Somnolence	5	331	1.26 [0.45, 3.51]	0.65
		Tremor	3	159	1.46 [0.40, 5.40]	0.57

**Comparator interventions**	**SOC**	**AEs**	**Number of studies**	**Number of participants**	**Risk Ratio (M-H, Random, 95% CI)**	***p* value**
	Gastrointestinal disorders	Constipation	4	239	2.22 [0.66, 7.42]	0.20
		Diarrhoea	4	284	0.94 [0.47, 1.85]	0.85
		Dry mouth	6	418	1.44 [0.91, 2.29]	0.12
		Dyspepsia	1	99	1.23 [0.06, 24.71]	0.89
		Dysphagia	1	80	3.00 [0.13, 71.51]	0.50
		Nausea	8	676	1.76 [0.97, 3.19]	0.06
		**Salivary hypersecretion**	**1**	**174**	**13.30 [1.78, 99.49]**	**0.01**
		Vomiting	6	418	1.75 [0.87, 3.51]	0.12
	General disorders	Asthenia	1	99	0.74 [0.03, 17.51]	0.85
		Chest pain	1	72	1.06 [0.21, 5.41]	0.94
		**Chest discomfort**	**1**	**174**	**4.30 [1.70, 10.88]**	**0.002**
		Crying	1	84	13.00 [0.76, 223.67]	0.08
		Decreased energy	3	159	1.19 [0.68, 2.11]	0.54
		Fatigue	6	418	1.12 [0.51, 2.47]	0.77
		Feeling cold	2	254	0.95 [0.12, 7.83]	0.96
		**Feeling hot**	**1**	**174**	**2.22 [1.20, 4.11]**	**0.01**
		Malaise	5	263	1.05 [0.40, 2.73]	0.93
	Ear and labyrinth disorders	Tinnitus	5	338	2.00 [0.93, 4.29]	0.08
		**Vertigo**	**2**	**273**	**3.66 [1.49, 8.99]**	**0.005**
	Eye disorders	**Vision blurred**	**4**	**333**	**3.85 [2.24, 6.63]**	**< 0.00001**
	Skin disorders	Dermatitis	1	99	0.08 [0.00, 1.95]	0.12
		Dermatitis contact	1	99	0.74 [0.03, 17.51]	0.85
		Dry skin	2	105	1.28 [0.58, 2.87]	0.54
		**Hyperhidrosis**	**4**	**333**	**2.29 [1.33, 3.95]**	**0.003**
		Pruritus	4	164	2.04 [0.69, 6.06]	0.20
		Rash	2	105	2.77 [0.50, 15.49]	0.25
	Investigations	Blood pressure increased	2	153	1.37 [0.51, 3.73]	0.53
		Blood testosterone abnormal	1	99	0.74 [0.03, 17.51]	0.85
		Heart rate increased	1	80	3.00 [0.13, 71.51]	0.50
		Hepatic enzyme increased	1	99	0.74 [0.03, 17.51]	0.85
		White blood cell count decreased	1	99	0.74 [0.03, 17.51]	0.85
	Musculoskeletal and connective tissue disorders	Arthralgia	1	99	0.08 [0.00, 1.95]	0.12
		Back pain	1	99	0.24 [0.02, 3.63]	0.30
		Musculoskeletal pain	1	80	1.50 [0.26, 8.50]	0.65
		Pain in extremity	1	99	1.23 [0.06, 24.71]	0.89
	Cardiac disorders	Palpitations	3	279	2.02 [0.88, 4.63]	0.10

Comparator interventions	SOC	AEs	Number of studies	Number of participants	Risk Ratio (M-H, Random, 95% CI)	*p* value
		Tachycardia	1	99	1.23 [0.06, 24.71]	0.89
	Vascular disorders	Flushing	1	99	0.74 [0.03, 17.51]	0.85
		Hot flush	1	99	0.74 [0.03, 17.51]	0.85
		Hypertension	1	99	0.74 [0.03, 17.51]	0.85
	Renal and urinary disorders	Dysuria	2	126	1.49 [0.12, 18.21]	0.76
		Pollakiuria	2	105	2.71 [0.48, 15.19]	0.26
	Infections and infestations	Gastroenteritis viral	1	99	0.08 [0.00, 1.95]	0.12
		Respiratory tract infection	1	99	0.74 [0.03, 17.51]	0.85
		Sinusitis	1	99	0.08 [0.00, 1.95]	0.12
		Tooth abscess	1	99	0.74 [0.03, 17.51]	0.85
		Upper respiratory tract infection	1	99	0.74 [0.03, 17.51]	0.85
		Viral upper respiratory tract infection	1	99	0.74 [0.03, 17.51]	0.85
	Injury, poisoning and procedural complications	Exposure to toxic agent	1	99	0.74 [0.03, 17.51]	0.85
		Fall	1	99	0.74 [0.03, 17.51]	0.85
		Overdose	1	99	0.74 [0.03, 17.51]	0.85
	Respiratory, thoramediastinal disorders	Cough	1	99	0.74 [0.03, 17.51]	0.85
		**Dyspnoea**	**1**	**174**	**3.58 [1.23, 10.45]**	**0.02**
		Nasal congestion	1	99	0.24 [0.02, 3.63]	0.30
	Metabolism and nutrition disorders	Increased appetite	1	99	0.74 [0.03, 17.51]	0.85
	Reproductive system and breast disorders	Sexual dysfunction	1	54	1.16 [0.18, 7.64]	0.88
**ECT**						
	Psychiatric disorders	**Affect lability**	**1**	**181**	**4.29 [1.26, 14.53]**	**0.02**
		**Anxiety**	**1**	**181**	**2.36 [1.48, 3.77]**	**0.0003**
		Confusional state	1	181	0.88 [0.55, 1.40]	0.59
		**Dissociation**	**2**	**220**	**5.74 [1.06, 31.16]**	**0.04**
		**Euphoric mood**	**1**	**181**	**40.55 [2.49, 660.53]**	**0.009**
		Paranoia	1	181	0.49 [0.05, 5.36]	0.56
		Sleep disorder	1	181	4.95 [0.24, 101.60]	0.30
		Suicide attempt	1	181	0.66 [0.19, 2.26]	0.51
	Nervous system disorders	**Amnesia**	**1**	**181**	**0.18 [0.09, 0.35]**	**< 0.01**
		**Headache**	**2**	**220**	**0.28 [0.17, 0.46]**	**< 0.01**
		Hypertonia	1	181	0.25 [0.03, 2.17]	0.21

Comparator interventions	SOC	AEs	Number of studies	Number of participants	Risk Ratio (M-H, Random, 95% CI)	*p* value
		**Memory impairment**	**1**	**39**	**0.02 [0.00, 0.39]**	**0.009**
		Myoclonus	1	181	0.33 [0.01, 7.99]	0.50
		Nystagmus	1	39	2.32 [0.12, 44.99]	0.58
		Paraesthesia	1	181	1.48 [0.25, 8.67]	0.66
		Seizure	1	181	0.20 [0.01, 4.06]	0.29
	Gastrointestinal disorders	Abdominal pain	1	181	0.14 [0.01, 2.70]	0.19
		Constipation	1	181	4.95 [0.59, 41.49]	0.14
		Diarrhoea	1	181	4.95 [0.59, 41.49]	0.14
		**Dry mouth**	**1**	**181**	**23.74 [3.28, 171.75]**	**0.002**
		Nausea	2	220	0.78 [0.18, 3.42]	0.74
		**Salivary hypersecretion**	**1**	**181**	**23.74 [3.28, 171.75]**	**0.002**
		Throat irritation	1	181	0.20 [0.02, 1.66]	0.14
		Vomiting	1	181	0.33 [0.07, 1.59]	0.17
	General disorders	Fatigue	1	181	0.95 [0.59, 1.53]	0.83
		Swelling	1	181	0.49 [0.05, 5.36]	0.56
	Ear and labyrinth disorders	Tinnitus	1	181	0.33 [0.03, 3.11]	0.33
		**Vertigo**	**1**	**181**	**2.53 [1.81, 3.54]**	**< 0.01**
	Eye disorders	**Diplopia**	**1**	**181**	**13.85 [3.40, 56.41]**	**0.0002**
		**Vision blurred**	**1**	**181**	**42.53 [2.62, 691.67]**	**0.008**
	Skin disorders	Skin irritation	1	181	2.97 [0.31, 27.99]	0.34
	Investigations	Blood pressure systolic increased	1	18	7.00 [0.41, 118.69]	0.18
		Heart rate increased	1	18	7.00 [0.41, 118.69]	0.18
		**Oxygen saturation decreased**	**1**	**181**	**0.33 [0.11, 0.98]**	**0.05**
	Musculoskeletal and connective tissue disorders	Musculoskeletal pain	1	39	0.15 [0.01, 3.55]	0.24
		**Myalgia**	**1**	**181**	**0.23 [0.15, 0.35]**	**< 0.01**
	Cardiac disorders	Bradycardia	1	181	0.08 [0.00, 1.33]	0.08
		Tachycardia	1	181	0.08 [0.00, 1.33]	0.08
	Vascular disorders	Hypotension	1	181	0.33 [0.01, 7.99]	0.50
		Thrombosis	1	181	0.49 [0.05, 5.36]	0.56
	Renal and urinary disorders	Urinary retention	1	181	2.97 [0.12, 71.89]	0.50
	Infections and infestations	Infection	1	181	2.97 [0.12, 71.89]	0.50
	Respiratory, thoracic and mediastinal disorders	Bronchospasm	1	181	0.09 [0.01, 1.60]	0.10
		Laryngospasm	1	181	0.09 [0.01, 1.60]	0.10

AEs, adverse events; ECT, electroconvulsive therapy; SOC, system organ class.

Supplementary Table 6

Subgroup analyses comparing the primary outcomes between repeated and single dosing of ketamine.

Outcome	Repeated dosing		Single dosing	
	**Risk Ratio (95% CI)**	***p* value**	**Risk Ratio (95% CI)**	***p* value**
Dropout due to AEs	**4.06 [1.63, 10.11]**	**0.003**	N/A	
Number of subjects with at least one AE	1.52 [0.76, 3.02]	0.24	1.44 [0.69, 3.00]	0.34
Number of subjects with at least one serious AE	0.65 [0.39, 1.10]	0.11	0.88 [0.24, 3.21]	0.85

AEs, adverse events; N/A, Not available.

Supplementary Table 7

Subgroup analyses comparing the primary outcomes between low-dose and very low-dose ketamine.

Outcome	Low-dose (0.5-1.0 mg/kg iv)		Very low-dose (< 0.5 mg/kg iv)	
	**Risk Ratio (95% CI)**	***p* value**	**Risk Ratio (95% CI)**	***p* value**
Dropout due to AEs	**4.25 [1.64, 11.02]**	**0.003**	N/A	
Number of subjects with at least one AE	**1.52 [1.01, 2.28]**	**0.04**	0.94 [0.63, 1.39]	0.74
Number of subjects with at least one serious AE	0.67 [0.41, 1.11]	0.12	0.84 [0.09, 7.64]	0.88
Treatment-emergent AEs[a]				
Affect lability	**4.29 [1.26, 14.53]**	**0.02**	N/A	
Anxiety	**1.96 [1.25, 3.07]**	**0.003**	0.48 [0.06, 3.92]	0.49
Depersonalisation/Derealisation disorder	**4.27 [2.15, 8.48]**	**< 0.0001**	N/A	
Dissociation	**4.44 [2.81, 7.02]**	**< 0.0001**	2.73 [0.30, 25.05]	0.38
Euphoric Mood	**10.14 [3.10, 33.12]**	**0.0001**	7.43 [0.43, 129.55]	0.17
Moodiness	**10.74 [2.60, 44.43]**	**0.001**	N/A	
Restlessness	**1.56 [1.11, 2.19]**	**0.01**	0.29 [0.04, 2.07]	0.22
Dizziness	**3.05 [1.81, 5.15]**	**< 0.0001**	0.78 [0.21, 2.86]	0.71
Dysgeusia	**2.34 [1.32, 4.15]**	**0.004**	N/A	
Hypoaesthesia	**8.42 [1.10, 64.21]**	**0.04**	N/A	
Movement Disorder	**4.09 [1.61, 10.41]**	**0.003**	N/A	
Paraesthesia	**3.25 [1.11, 9.46]**	**0.03**	N/A	
Paralysis	**5.35 [3.08, 9.29]**	**< 0.0001**	N/A	
Constipation	2.57 [0.91, 7.24]	0.07	2.89 [0.31, 27.27]	0.35
Nausea	**1.77 [1.06, 2.95]**	**0.03**	0.69 [0.23, 2.09]	0.51
Salivary hypersecretion	**16.42 [4.66, 57.88]**	**< 0.0001**	N/A	
Chest discomfort	**4.30 [1.70, 10.88]**	**0.002**	N/A	
Feeling hot	**2.22 [1.20, 4.11]**	**0.01**	N/A	
Vertigo	**3.02 [2.09, 4.38]**	**< 0.0001**	N/A	
Diplopia	**10.61 [2.94, 38.32]**	**0.0003**	N/A	
Vision Blurred	**4.22 [2.59, 6.89]**	**< 0.0001**	2.74 [0.44, 17.04]	0.28
Hyperhidrosis	**2.27 [1.31, 3.91]**	**0.003**	12.60 [0.76, 208.61]	0.08
Dyspnoea	**3.49 [1.26, 9.61]**	**0.02**	N/A	

[a]The threshold for conducting subgroup analyses by dose for treatment-emergent AEs was that the incidences were significantly higher in the ketamine group than those reported in the control group from the meta-analyses. AEs, adverse events; N/A, Not available; IV, intravenous.

Supplementary Table 8

The GRADE assessments of the evidence certainty for the primary outcomes of ketamine.

Quality assessment							No of patients		Effect		Quality	Importance
**No of studies**	**Design**	**Risk of bias**	**Inconsistency**	**Indirectness**	**Imprecision**	**Other considerations**	**Ketamine**	**Control**	**Relative (95% CI)**	**Absolute**		
**OUTCOME: Dropouts due to AEs (follow-up 3 to 4 weeks; assessed with: Number of participants withdrew early due to AE)**												
5	randomised trials	serious[1]	no serious inconsistency	no serious indirectness	serious[2]	strong association[3]	23/251 (9.2%)	4/243 (1.6%)	RR 4.06 (1.63 to 10.11)	50 more per 1000 (from 10 more to 150 more)	⊕⊕⊕⊖ MODERATE	CRITICAL
								0%[4]		-		
**OUTCOME: Number of patients experiencing at least one AE (follow-up 3~28 days; assessed with: Total number of patients experiencing at least one AE)**												
8	randomised trials	serious[1]	serious[5]	no serious indirectness	no serious imprecision	none	219/299 (73.2%)	145/227 (63.9%)	RR 1.36 (1.01 to 1.84)	230 more per 1000 (from 6 more to 537 more)	⊕⊕⊖⊖ LOW	CRITICAL
								28.6%[6]		103 more per 1000 (from 3 more to 240 more)		
								94.4%[6]		340 more per 1000 (from 9 more to 793 more)		

**OUTCOME: Number of patients experiencing at least one serious AE (follow-up 3~84 days; assessed with: Total number of patients experiencing at least one serious AE)**												
10	randomised trials	serious[1]	no serious inconsistency	no serious indirectness	no serious imprecision	none	28/570 (4.9%)	33/389 (8.5%)	RR 0.68 (0.42 to 1.11)	27 fewer per 1000 (from 49 fewer to 9 more)	⊕⊕⊕⊖ MODERATE	CRITICAL
								3.4%[6]		11 fewer per 1000 (from 20 fewer to 4 more)		
								7.7%[6]		25 fewer per 1000 (from 45 fewer to 8 more)		

[1] The randomization method of the included studies were assessed as high risk.

[2] The confidence interval of the pooled result was relatively wide.

[3] The effect size (risk ratio, RR) was greater than 2.

[4] There was little variation in baseline risks among the studies included in the meta-analysis.

[5] There was high heterogeneity among the included studies.

[6] There was a large variation in baseline risks among the studies included in the meta-analysis.

Supplementary Table 9

Combined effect of esketamine after excluding each individual study in turn.

Outcome	Culled references	Risk Ratio (M-H, Random, 95% CI)	p^a value	I^2/%	p^b value
Number of participants with at least one AE	Canuso 2018	1.37 [1.23, 1.52]	< 0.00001	79	< 0.00001
	Chen 2023	1.35 [1.21, 1.52]	< 0.00001	80	< 0.00001
	Daly 2018	1.35 [1.21, 1.50]	< 0.00001	80	< 0.00001
	**Daly 2019**	**1.28 [1.20, 1.37]**	**< 0.00001**	**47**	**0.12**
	Fedgchin 2019	1.34 [1.21, 1.49]	< 0.00001	79	< 0.00001
	Fu 2020	1.37 [1.22, 1.53]	< 0.00001	79	< 0.00001
	Ionescu 2021	1.37 [1.23, 1.54]	< 0.00001	79	< 0.00001
	Ochs-Ross 2020	1.36 [1.23, 1.52]	< 0.00001	80	< 0.00001
	Popova 2019	1.33 [1.20, 1.47]	< 0.00001	76	< 0.00001
	Reif 2023	1.38 [1.23, 1.54]	< 0.00001	75	< 0.0001
	Singh 2016 a	1.35 [1.22, 1.50]	< 0.00001	80	< 0.00001
	Takahashi 2021	1.34 [1.21, 1.50]	< 0.00001	79	< 0.00001

[a] This *p* value is the statistical value for overall effect. [b] This *p* value is the statistical value for heterogeneity. AE, adverse event.

Supplementary Table 10

Meta-analyses for each specific AE associated with esketamine.

SOC	AEs	Number of studies	Number of participants	Risk Ratio (M-H, Random, 95% CI)	*p* value
Psychiatric disorders	Aggression	1	225	0.33 [0.01, 8.03]	0.50
	Agitation	1	66	8.00 [0.45, 142.90]	0.16
	Anxiety	8	1771	1.59 [0.97, 2.61]	0.07
	Apathy	1	111	1.11 [0.40, 3.08]	0.85
	**Confusion**	**2**	**967**	**19.43 [3.79, 99.75]**	**0.0004**
	**Depersonalisation/ Derealisation disorder**	**1**	**227**	**20.82 [1.23, 351.07]**	**0.04**
	Depression	3	796	0.80 [0.14, 4.61]	0.81
	Depression suicidal	1	225	1.98 [0.18, 21.55]	0.57
	Depressive symptom	1	66	2.67 [0.11, 63.17]	0.54
	**Dissociation**	**13**	**3020**	**7.36 [4.73, 11.43]**	**< 0.00001**
	Dissociative disorder	1	89	8.95 [0.53, 151.77]	0.13
	Dysphoria	1	137	8.14 [0.45, 148.28]	0.16
	**Euphoric mood**	**5**	**1091**	**4.19 [1.81, 9.67]**	**0.0008**
	Hallucination	1	202	4.61 [0.24, 88.06]	0.31
	Hallucination, Visual	1	252	7.00 [0.87, 56.07]	0.07
	Insomnia	8	1563	0.95 [0.66, 1.37]	0.80
	Irritability	1	111	3.79 [0.44, 32.84]	0.23
	Nervousness	1	111	10.43 [0.59, 184.24]	0.11
	Panic attack	1	66	0.18 [0.01, 3.57]	0.26
	Sleep Disorder	1	111	1.16 [0.52, 2.57]	0.72
	Suicidal ideation	4	720	0.82 [0.25, 2.68]	0.75
	Suicide attempt	3	1122	1.18 [0.36, 3.88]	0.78
Nervous system disorders	Ataxia	1	111	6.63 [0.84, 52.13]	0.07
	Disturbance in attention	1	111	4.74 [0.23, 96.56]	0.31
	**Dizziness**	**14**	**3131**	**3.94 [3.13, 4.95]**	**< 0.00001**
	**Dizziness postural**	**8**	**1695**	**5.06 [2.48, 10.31]**	**< 0.00001**
	Dysarthria	1	202	4.61 [0.24, 88.06]	0.31
	**Dysgeusia**	**10**	**2530**	**1.88 [1.24, 2.86]**	**0.003**
	Dyslalia	1	202	3.29 [0.16, 67.70]	0.44
	**Headache**	**14**	**3131**	**1.41 [1.10, 1.80]**	**0.006**
	Hypersomnia	1	89	2.98 [0.15, 60.30]	0.48
	**Hypoaesthesia**	**10**	**2643**	**6.51 [3.75, 11.29]**	**< 0.00001**
	Hypotonia	1	202	4.61 [0.24, 88.06]	0.31
	Lethargy	1	344	5.87 [0.77, 44.59]	0.09
	**Mental impairment**	**3**	**657**	**5.53 [1.29, 23.64]**	**0.02**
	**Paraesthesia**	**9**	**2127**	**4.38 [2.39, 8.02]**	**< 0.00001**
	**Sedation**	**8**	**2120**	**3.63 [1.40, 9.45]**	**0.008**
	**Somnolence**	**11**	**2879**	**1.93 [1.17, 3.19]**	**0.01**
	Tremor	2	410	1.93 [0.52, 7.20]	0.33

SOC	AEs	Number of studies	Number of participants	Risk Ratio (M-H, Random, 95% CI)	*p* value
	Tunnel vision	1	89	2.98 [0.15, 60.30]	0.48
Gastrointestinal disorders	Abdominal discomfort	1	111	1.58 [0.40, 6.29]	0.52
	Abdominal pain	1	66	0.18 [0.01, 3.57]	0.26
	Constipation	4	770	1.18 [0.42, 3.28]	0.75
	Diarrhoea	5	946	1.42 [0.82, 2.45]	0.22
	Dry mouth	5	679	1.51 [0.70, 3.23]	0.29
	Flatulence	1	66	0.44 [0.04, 4.65]	0.50
	**Hypoaesthesia oral**	**9**	**1837**	**7.42 [3.66, 15.05]**	**< 0.00001**
	**Nausea**	**14**	**3131**	**3.08 [2.12, 4.47]**	**< 0.00001**
	**Paraesthesia oral**	**6**	**1268**	**4.69 [2.28, 9.63]**	**< 0.0001**
	Salivary hypersecretion	1	111	0.19 [0.01, 3.86]	0.28
	Toothache	1	66	0.18 [0.01, 3.57]	0.26
	**Vomiting**	**13**	**3042**	**3.75 [2.31, 6.10]**	**< 0.00001**
General disorders and administration site conditions	**Asthenia**	**2**	**454**	**5.52 [1.44, 21.18]**	**0.01**
	Fatigue	7	1803	1.13 [0.68, 1.89]	0.64
	Feeling abnormal	2	155	5.77 [0.74, 45.12]	0.09
	Feeling cold	1	66	4.44 [0.22, 89.16]	0.33
	**Feeling drunk**	**5**	**1248**	**6.39 [2.64, 15.48]**	**< 0.0001**
	Hot flush	1	111	3.79 [0.44, 32.84]	0.23
	Malaise	1	202	8.56 [0.49, 149.90]	0.14
	Thirst	1	202	3.29 [0.16, 67.70]	0.44
Ear and labyrinth disorders	Hyperacusis	2	268	4.53 [0.55, 37.08]	0.16
	Tinnitus	3	379	1.65 [0.47, 5.76]	0.43
	**Vertigo**	**11**	**2532**	**7.34 [4.75, 11.33]**	**< 0.00001**
Eye disorders	Blepharospasm	1	66	0.18 [0.01, 3.57]	0.26
	**Diplopia**	**4**	**792**	**9.06 [2.13, 38.61]**	**0.003**
	Eye pruritus	1	137	0.30 [0.01, 7.27]	0.46
	**Vision blurred**	**10**	**2504**	**4.26 [2.03, 8.95]**	**0.0001**
Skin and subcutaneous tissue disorders	Acne	1	66	4.44 [0.22, 89.16]	0.33
	Hyperhidrosis	2	293	2.28 [0.66, 7.86]	0.19
	Rash	3	229	0.77 [0.22, 2.72]	0.69
Investigations	Blood pressure diastolic increased	1	202	0.66 [0.09, 4.56]	0.67
	**Blood pressure increased**	**10**	**2643**	**3.17 [2.33, 4.30]**	**< 0.00001**
	Respiratory rate decreased	1	202	3.29 [0.16, 67.70]	0.44
	Weight increased	4	1049	0.80 [0.20, 3.22]	0.75
Musculoskeletal and connective tissue disorders	Muscular weakness	1	202	5.93 [0.32, 108.61]	0.23
	Muscle Contracture	1	111	1.52 [0.53, 4.35]	0.44
	Back pain	1	670	1.90 [0.86, 4.20]	0.11
	Arrhythmia	1	227	0.33 [0.01, 8.03]	0.50

SOC	AEs	Number of studies	Number of participants	Risk Ratio (M-H, Random, 95% CI)	*p* value
Cardiac disorders	Arrhythmia	1	227	0.33 [0.01, 8.03]	0.50
	Bradycardia	1	236	0.33 [0.01, 8.10]	0.50
	Palpitations	1	202	3.28 [0.39, 27.55]	0.27
	Pericardial effusion	1	227	0.33 [0.01, 8.03]	0.50
	**Tachycardia**	**1**	**236**	**4.50 [1.00, 20.39]**	**0.05**
Vascular disorders	Cyanosis	1	236	3.00 [0.12, 72.90]	0.50
	Hypertension	2	325	2.33 [0.38, 14.25]	0.36
	Hypotension	1	236	5.00 [0.24, 103.04]	0.30
	Thrombophlebitis	1	52	0.77 [0.03, 17.58]	0.87
Renal and urinary disorders	Dysuria	1	111	1.26 [0.30, 5.38]	0.75
	Pollakiuria	3	521	1.07 [0.31, 3.68]	0.91
	Polyuria	1	89	2.98 [0.15, 60.30]	0.48
Infections and infestations	Nasopharyngitis	2	722	1.88 [0.94, 3.76]	0.07
	Tooth infection	1	52	0.09 [0.00, 1.95]	0.12
	Upper respiratory tract infection	2	289	0.23 [0.03, 2.08]	0.19
	Urinary tract infection	1	137	5.42 [0.67, 43.81]	0.11
	Viral upper respiratory tract infection	1	297	0.87 [0.40, 1.92]	0.74
Respiratory, thoracic and mediastinal disorders	Chest pain	1	111	2.84 [0.30, 26.49]	0.36
	Dyspnoea	1	236	4.00 [0.45, 35.26]	0.21
	Epistaxis	1	66	0.18 [0.01, 3.57]	0.26
	Intranasal paraesthesia	1	66	0.18 [0.01, 3.57]	0.26
	Nasal congestion	1	66	0.44 [0.04, 4.65]	0.50
	Nasal discomfort	7	1357	1.43 [0.77, 2.67]	0.26
	Oropharyngeal pain	5	636	1.36 [0.60, 3.08]	0.46
	Pharyngeal hypoaesthesia	1	66	4.44 [0.22, 89.16]	0.33
	Pneumothorax	1	227	0.33 [0.01, 8.03]	0.50
	Respiratory distress	1	111	2.84 [0.60, 13.48]	0.19
	Rhinalgia	1	66	0.18 [0.01, 3.57]	0.26
	Rhinorrhoea	1	66	0.18 [0.01, 3.57]	0.26
	**Throat irritation**	**7**	**1498**	**2.28 [1.29, 4.02]**	**0.005**
Metabolism and nutrition disorders	Decreased appetite	2	334	1.30 [0.36, 4.69]	0.69
	Diabetic ketoacidosis	1	225	2.97 [0.12, 72.23]	0.50
Hepatobiliary disorders	Hypertransaminasaemia	1	225	0.33 [0.01, 8.03]	0.50

AEs, adverse events; SOC, system organ class.

Supplementary Table 11

Subgroup analyses for each specific AEs associated with esketamine.

Comparator interventions	SOC	AE	Number of studies	Number of participants	Risk Ratio (M-H, Random, 95% CI)	*p* value
**Placebo**						
	Psychiatric disorders	Aggression	1	225	0.33 [0.01, 8.03]	0.50
		Agitation	1	66	8.00 [0.45, 142.90]	0.16
		Anxiety	8	1771	1.59 [0.97, 2.61]	0.07
		Apathy	1	111	1.11 [0.40, 3.08]	0.85
		**Confusion**	**1**	**297**	**18.13 [1.06, 308.70]**	**0.05**
		**Depersonalisation/derealisation disorder**	**1**	**227**	**20.82 [1.23, 351.07]**	**0.04**
		Depression	3	796	0.80 [0.14, 4.61]	0.81
		Depression suicidal	1	225	1.98 [0.18, 21.55]	0.57
		Depressive symptom	1	66	2.67 [0.11, 63.17]	0.54
		**Dissociation**	**12**	**2350**	**6.19 [4.50, 8.52]**	**< 0.00001**
		Dissociative disorder	1	89	8.95 [0.53, 151.77]	0.13
		Dysphoria	1	137	8.14 [0.45, 148.28]	0.16
		**Euphoric mood**	**5**	**1091**	**4.19 [1.81, 9.67]**	**0.0008**
		Hallucination	1	202	4.61 [0.24, 88.06]	0.31
		Hallucination, Visual	1	252	7.00 [0.87, 56.07]	0.07
		Insomnia	8	1563	0.95 [0.66, 1.37]	0.80
		Irritability	1	111	3.79 [0.44, 32.84]	0.23
		Nervousness	1	111	10.43 [0.59, 184.24]	0.11
		Panic attack	1	66	0.18 [0.01, 3.57]	0.26
		Sleep Disorder	1	111	1.16 [0.52, 2.57]	0.72
		Suicidal ideation	4	720	0.82 [0.25, 2.68]	0.75
		Suicide attempt	2	452	0.99 [0.25, 3.90]	0.99
	Nervous system disorders	Ataxia	1	111	6.63 [0.84, 52.13]	0.07
		Disturbance in attention	1	111	4.74 [0.23, 96.56]	0.31
		**Dizziness**	**13**	**2461**	**3.57 [2.88, 4.44]**	**< 0.00001**
		**Dizziness postural**	**8**	**1695**	**5.06 [2.48, 10.31]**	**< 0.0001**
		Dysarthria	1	202	4.61 [0.24, 88.06]	0.31
		**Dysgeusia**	**9**	**1860**	**1.68 [1.23, 2.31]**	**0.001**
		Dyslalia	1	202	3.29 [0.16, 67.70]	0.44
		**Headache**	**13**	**2461**	**1.33 [1.02, 1.72]**	**0.03**
		Hypersomnia	1	89	2.98 [0.15, 60.30]	0.48

Comparator interventions	SOC	AE	Number of studies	Number of participants	Risk Ratio (M-H, Random, 95% CI)	*p* value
		**Hypoaesthesia**	**9**	**1973**	**5.96 [3.36, 10.57]**	**< 0.00001**
		Hypotonia	1	202	4.61 [0.24, 88.06]	0.31
		Lethargy	1	344	5.87 [0.77, 44.59]	0.09
		**Mental impairment**	**3**	**657**	**5.53 [1.29, 23.64]**	**0.02**
		**Paraesthesia**	**8**	**1457**	**3.28 [2.09, 5.16]**	**< 0.00001**
		**Sedation**	**7**	**1450**	**4.96 [2.55, 9.64]**	**< 0.00001**
		**Somnolence**	**10**	**2209**	**2.17 [1.46, 3.23]**	**0.0001**
		Tremor	2	410	1.93 [0.52, 7.20]	0.33
		Tunnel vision	1	89	2.98 [0.15, 60.30]	0.48
	Gastrointestinal disorders	Abdominal discomfort	1	111	1.58 [0.40, 6.29]	0.52
		Abdominal pain	1	66	0.18 [0.01, 3.57]	0.26
		Constipation	4	770	1.18 [0.42, 3.28]	0.75
		Diarrhoea	5	946	1.42 [0.82, 2.45]	0.22
		Dry mouth	5	679	1.51 [0.70, 3.23]	0.29
		Flatulence	1	66	0.44 [0.04, 4.65]	0.50
		**Hypoaesthesia oral**	**9**	**1837**	**7.42 [3.66, 15.05]**	**< 0.00001**
		**Nausea**	**13**	**2461**	**2.69 [1.96, 3.69]**	**< 0.00001**
		**Paraesthesia oral**	**6**	**1268**	**4.69 [2.28, 9.63]**	**< 0.0001**
		Salivary hypersecretion	1	111	0.19 [0.01, 3.86]	0.28
		Toothache	1	66	0.18 [0.01, 3.57]	0.26
		**Vomiting**	**12**	**2372**	**3.34 [2.00, 5.56]**	**< 0.00001**
	General disorders and administration site conditions	**Asthenia**	**2**	**454**	**5.52 [1.44, 21.18]**	**0.01**
		Fatigue	6	1133	1.46 [0.90, 2.39]	0.12
		Feeling abnormal	2	155	5.77 [0.74, 45.12]	0.09
		Feeling cold	1	66	4.44 [0.22, 89.16]	0.33
		**Feeling drunk**	**5**	**1248**	**6.39 [2.64, 15.48]**	**< 0.0001**
		Hot flush	1	111	3.79 [0.44, 32.84]	0.23
		Malaise	1	202	8.56 [0.49, 149.90]	0.14
		Thirst	1	202	3.29 [0.16, 67.70]	0.44
	Ear and labyrinth disorders	Hyperacusis	2	268	4.53 [0.55, 37.08]	0.16
		Tinnitus	3	379	1.65 [0.47, 5.76]	0.43
		**Vertigo**	**10**	**1862**	**6.15 [3.85, 9.84]**	**< 0.00001**
	Eye disorders	Blepharospasm	1	66	0.18 [0.01, 3.57]	0.26
		**Diplopia**	**4**	**792**	**9.06 [2.13, 38.61]**	**0.003**
		Eye pruritus	1	137	0.30 [0.01, 7.27]	0.46
		**Vision blurred**	**9**	**1834**	**4.03 [1.75, 9.28]**	**0.001**
		Acne	1	66	4.44 [0.22, 89.16]	0.33
		Hyperhidrosis	2	293	2.28 [0.66, 7.86]	0.19

**Comparator interventions**	**SOC**	**AE**	**Number of studies**	**Number of participants**	**Risk Ratio (M-H, Random, 95% CI)**	***p* value**
	Skin and subcutaneous tissue disorders	Rash	3	229	0.77 [0.22, 2.72]	0.69
	Investigations	Blood pressure diastolic increased	1	202	0.66 [0.09, 4.56]	0.67
		**Blood pressure increased**	**9**	**1973**	**2.93 [2.13, 4.04]**	**< 0.00001**
		Respiratory rate decreased	1	202	3.29 [0.16, 67.70]	0.44
		Weight increased	3	379	1.32 [0.47, 3.70]	0.60
	Musculoskeletal and connective tissue disorders	Muscular weakness	1	202	5.93 [0.32, 108.61]	0.23
		Muscle Contracture	1	111	1.52 [0.53, 4.35]	0.44
	Cardiac disorders	Arrhythmia	1	227	0.33 [0.01, 8.03]	0.50
		Bradycardia	1	236	0.33 [0.01, 8.10]	0.50
		Palpitations	1	202	3.28 [0.39, 27.55]	0.27
		Pericardial effusion	1	227	0.33 [0.01, 8.03]	0.50
		**Tachycardia**	**1**	**236**	**4.50 [1.00, 20.39]**	**0.05**
	Vascular disorders	Cyanosis	1	236	3.00 [0.12, 72.90]	0.50
		Hypertension	2	325	2.33 [0.38, 14.25]	0.36
		Hypotension	1	236	5.00 [0.24, 103.04]	0.30
		Thrombophlebitis	1	52	0.77 [0.03, 17.58]	0.87
	Renal and urinary disorders	Dysuria	1	111	1.26 [0.30, 5.38]	0.75
		Pollakiuria	3	521	1.07 [0.31, 3.68]	0.91
		Polyuria	1	89	2.98 [0.15, 60.30]	0.48
	Infections and infestations	Nasopharyngitis	1	52	1.28 [0.07, 24.76]	0.87
		Tooth infection	1	52	0.09 [0.00, 1.95]	0.12
		Upper respiratory tract infection	2	289	0.23 [0.03, 2.08]	0.19
		Urinary tract infection	1	137	5.42 [0.67, 43.81]	0.11
		Viral upper respiratory tract infection	1	297	0.87 [0.40, 1.92]	0.74
	Respiratory, thoracic and mediastinal disorders	Chest pain	1	111	2.84 [0.30, 26.49]	0.36
		Dyspnoea	1	236	4.00 [0.45, 35.26]	0.21
		Epistaxis	1	66	0.18 [0.01, 3.57]	0.26
		Intranasal paraesthesia	1	66	0.18 [0.01, 3.57]	0.26
		Nasal congestion	1	66	0.44 [0.04, 4.65]	0.50

Comparator interventions	SOC	AE	Number of studies	Number of participants	Risk Ratio (M-H, Random, 95% CI)	*p* value
		Nasal discomfort	7	1357	1.43 [0.77, 2.67]	0.26
		Oropharyngeal pain	5	636	1.36 [0.60, 3.08]	0.46
		Pharyngeal hypoaesthesia	1	66	4.44 [0.22, 89.16]	0.33
		Pneumothorax	1	227	0.33 [0.01, 8.03]	0.50
		Respiratory distress	1	111	2.84 [0.60, 13.48]	0.19
		Rhinalgia	1	66	0.18 [0.01, 3.57]	0.26
		Rhinorrhoea	1	66	0.18 [0.01, 3.57]	0.26
		**Throat irritation**	**7**	**1498**	**2.28 [1.29, 4.02]**	**0.005**
	Metabolism and nutrition disorders	Decreased appetite	2	334	1.30 [0.36, 4.69]	0.69
		Diabetic ketoacidosis	1	225	2.97 [0.12, 72.23]	0.50
	Hepatobiliary disorders	Hypertransaminasaemia	1	225	0.33 [0.01, 8.03]	0.50
**Quetiapine**						
	Psychiatric disorders	**Confusion**	**1**	**670**	**20.12 [2.72, 149.06]**	**0.003**
		**Dissociation**	**1**	**670**	**47.28 [11.75, 190.28]**	**< 0.00001**
		Suicide attempt	1	670	2.01 [0.18, 22.08]	0.57
	Nervous system disorders	**Dizziness**	**1**	**670**	**5.60 [3.86, 8.14]**	**< 0.00001**
		**Dysgeusia**	**1**	**670**	**40.24 [5.56, 291.01]**	**0.0003**
		**Headache**	**1**	**670**	**1.92 [1.37, 2.69]**	**0.0001**
		**Hypoaesthesia**	**1**	**670**	**19.11 [2.57, 141.96]**	**0.004**
		**Paraesthesia**	**1**	**670**	**18.61 [4.52, 76.59]**	**< 0.0001**
		Sedation	1	670	0.76 [0.45, 1.30]	0.32
		**Somnolence**	**1**	**670**	**0.66 [0.48, 0.91]**	**0.01**
	Gastrointestinal disorders	**Nausea**	**1**	**670**	**8.22 [4.60, 14.67]**	**< 0.00001**
		**Vomiting**	**1**	**670**	**7.24 [2.88, 18.23]**	**< 0.0001**
	General disorders and administration site conditions	**Fatigue**	**1**	**670**	**0.56 [0.33, 0.97]**	**0.04**
	Ear and labyrinth disorders	**Vertigo**	**1**	**670**	**21.13 [6.70, 66.60]**	**< 0.00001**
	Eye disorders	**Vision blurred**	**1**	**670**	**7.04 [2.12, 23.38]**	**0.001**
	Investigations	**Blood pressure increased**	**1**	**670**	**7.04 [2.50, 19.86]**	**0.0002**
		**Weight increased**	**1**	**670**	**0.22 [0.11, 0.44]**	**< 0.0001**

**Comparator interventions**	**SOC**	**AE**	**Number of studies**	**Number of participants**	**Risk Ratio (M-H, Random, 95% CI)**	***p* value**
	Musculoskeletal and connective tissue disorders	Back pain	1	670	1.90 [0.86, 4.20]	0.11
	Infections and infestations	Nasopharyngitis	1	670	1.92 [0.94, 3.92]	0.07

AEs, adverse events; SOC, system organ class.

Supplementary Table 12

Subgroup analyses by dose comparing the primary outcomes of esketamine.

Outcome	Esketamine dose (28 mg)		Esketamine dose (56mg)		Esketamine dose (84mg)	
	Risk Ratio (95% CI)	*p* value	Risk Ratio (95% CI)	*p* value	Risk Ratio (95% CI)	*p* value
Dropout due to AEs	1.33 [0.19, 9.12]	0.77	1.94 [0.61, 6.14]	0.26	**2.11 [1.10, 4.05]**	**0.02**
Number of subjects with at least one AE	1.13 [0.91, 1.41]	0.28	**1.47 [1.31, 1.66]**	**< 0.00001**	**1.30 [1.17, 1.43]**	**< 0.00001**
Number of subjects with at least one serious AE	1.95 [0.13, 30.40]	0.63	1.87 [0.21, 16.72]	0.58	0.96 [0.44, 2.09]	0.91
Treatment-emergent AEs[a]						
Depersonalisation/Derealisation Disorder	N/A		N/A		**20.82 [1.23, 351.07]**	**0.04**
Dissociation	2.76 [0.64, 11.90]	0.17	**5.04 [2.19, 11.59]**	**0.0001**	**5.50 [3.78, 8.01]**	**< 0.00001**
Euphoric Mood	N/A		**3.45 [0.91, 13.15]**	**0.07**	**5.43 [1.41, 20.97]**	**0.01**
Dizziness	**4.68 [1.91, 11.45]**	**0.0007**	**5.00 [2.41, 10.37]**	**< 0.0001**	**3.21 [2.17, 4.74]**	**< 0.00001**
Dizziness postural	N/A		1.10 [0.24, 5.10]	0.91	**5.67 [2.07, 15.53]**	**0.0007**
Dysgeusia	0.50 [0.11, 2.15]	0.35	0.92 [0.53, 1.60]	0.77	**1.44 [1.06, 1.97]**	**0.02**
Headache	1.14 [0.87, 1.49]	0.36	1.43 [0.89, 2.32]	0.14	**3.67 [1.47, 9.19]**	**0.006**
Hypoaesthesia	**3.41 [1.06, 11.00]**	**0.04**	**4.84 [1.97, 11.88]**	**0.0006**	**4.77 [2.31, 9.87]**	**< 0.0001**
Mental impairment	13.50 [0.71, 255.28]	0.08	**6.93 [1.24, 38.81]**	**0.03**	N/A	
Paraesthesia	N/A		**6.22 [1.89, 20.45]**	**0.003**	**3.50 [1.84, 6.66]**	**0.0001**
Sedation	5.43 [0.58, 50.92]	0.14	**8.44 [1.92, 37.00]**	**0.005**	**4.78 [2.35, 9.71]**	**< 0.0001**
Somnolence	1.39 [0.68, 2.86]	0.37	**1.81 [1.15, 2.85]**	**0.01**	**1.79 [1.29, 2.47]**	**0.0004**
Hypoaesthesia oral	**8.58 [1.00, 73.70]**	**0.05**	**9.02 [2.75, 29.57]**	**0.0003**	**5.22 [2.10, 12.97]**	**0.0004**
Nausea	1.71 [0.73, 4.00]	0.21	**2.34 [1.44, 3.80]**	**0.0006**	**2.34 [1.71, 3.20]**	**< 0.00001**
Paraesthesia oral	N/A		4.42 [0.98, 20.02]	0.05	2.61 [0.67, 10.25]	0.17
Vomiting	0.65 [0.07, 6.06]	0.71	2.59 [0.86, 7.77]	0.09	**2.98 [1.41, 6.29]**	**0.004**
Asthenia	9.64 [0.47, 196.30]	0.14	**28.93 [1.69, 494.32]**	**0.02**	N/A	
Feeling drunk	1.95 [0.13, 30.40]	0.63	**11.29 [2.07, 61.70]**	**0.005**	**5.35 [1.34, 21.43]**	**0.02**

**Outcome**	**Esketamine dose (28 mg)**		**Esketamine dose (56mg)**		**Esketamine dose (84mg)**	
	**Risk Ratio (95% CI)**	***p* value**	**Risk Ratio (95% CI)**	***p* value**	**Risk Ratio (95% CI)**	***p* value**
Vertigo	**8.04 [1.40, 46.22]**	**0.02**	**11.04 [3.69, 33.04]**	**< 0.0001**	**10.55 [4.41, 25.26]**	**< 0.00001**
Diplopia	5.79 [0.24, 138.97]	0.28	13.50 [0.71, 255.28]	0.08	7.75 [0.98, 61.54]	0.05
Vision blurred	N/A		16.71 [0.98, 286.07]	0.05	**2.97 [1.58, 5.57]**	**0.0007**
Blood pressure increased	**2.93 [1.30, 6.59]**	**0.009**	**2.91 [1.02, 8.35]**	**0.05**	**3.32 [2.10, 5.25]**	**< 0.00001**
Throat irritation	5.10 [0.22, 119.32]	0.31	1.23 [0.34, 4.46]	0.75	**2.24 [1.02, 4.88]**	**0.04**

[a]The threshold for conducting subgroup analyses by dose for treatment-emergent AEs was that the incidences were significantly higher in the esketamine group than those reported in the control group from the meta-analyses. AEs, adverse events; N/A, Not available.

Supplementary Table 13

The GRADE assessments of the evidence certainty for the primary outcomes of esketamine.

Quality assessment							No of patients		Effect		Quality	Importance
**No of studies**	**Design**	**Risk of bias**	**Inconsistency**	**Indirectness**	**Imprecision**	**Other considerations**	**Esketamine**	**Control**	**Relative (95% CI)**	**Absolute**		
**OUTCOME: Dropouts due to AEs (follow-up 1~32 weeks; assessed with: Number of participants withdrew early due to AE)**												
14	randomised trials	serious[1]	no serious inconsistency	no serious indirectness	no serious imprecision	dose response gradient[2]	86/1689 (5.1%)	61/1445 (4.2%)	-	42 fewer per 1000 (from 42 fewer to 42 fewer)	⊕⊕⊕⊕ HIGH	CRITICAL
								2.4%[3]		24 fewer per 1000 (from 24 fewer to 24 fewer)		
**OUTCOME: Number of patients experiencing at least one AE (follow-up 1~32 weeks; assessed with: Total number of patients experiencing at least one AE)**												
12	randomised trials	serious[1]	serious[4]	no serious indirectness	no serious imprecision	none	1287/1511 (85.2%)	822/1273 (64.6%)	RR 1.35 (1.22 to 1.49)	226 more per 1000 (from 142 more to 316 more)	⊕⊕⊝⊝ LOW	CRITICAL
								49.5%[5]		173 more per 1000 (from 109 more to 243 more)		

								77.9%[5]		273 more per 1000 (from 171 more to 382 more)		
**OUTCOME: Number of patients experiencing at least one serious AE (follow-up 1~32 weeks; assessed with: Total number of patients experiencing at least one serious AE)**												
12	randomised trials	serious[1]	no serious inconsistency	no serious indirectness	no serious imprecision	none	51/1512 (3.4%)	38/1294 (2.9%)	RR 1.14 (0.74 to 1.73)	4 more per 1000 (from 8 fewer to 21 more)	⊕⊕⊕⊝ MODERATE	CRITICAL
								1.6%[3]		2 more per 1000 (from 4 fewer to 12 more)		

[1] The randomization method of the included studies were assessed as high risk.

[2] The included studies showed that there was a dose-response relationship for this indicator.

[3] There was little variation in baseline risks among the studies included in the meta-analysis, taking the median of the control group risk.

[4] There was high heterogeneity among the included studies.

[5] There was a large variation in baseline risks among the studies included in the meta-analysis, taking the second lowest risk and the second highest risk of the control group.

Supplementary Table 14

Changes in ketamine-related CADSS scores from baseline to each predefined time point.

Measurement time post-dose	Number of Studies	Number of Participants	MD (IV, Random, 95% CI)	*p* value
**40 minutes**	**7**	**296**	**6.83 [2.71, 10.94]**	**0.001**
**60 minutes**	**1**	**174**	**6.50 [4.42, 8.58]**	**< 0.00001**
80 minutes	1	99	-0.03 [-0.85, 0.79]	0.94
2 hours	2	273	0.48 [-0.77, 1.72]	0.45
4 hours	2	60	0.12 [-1.27, 1.51]	0.87
1 week	1	27	1.63 [-2.94, 6.20]	0.48
2 weeks	1	27	1.93 [-2.63, 6.49]	0.41

CADSS, clinician administered dissociative states scale.

Supplementary Table 15

Subgroup analyses of ketamine dose on changes in CADSS scores at 40 minutes post-dose.

Ketamine dose	Number of Studies	Number of Participants	MD (IV, Random, 95% CI)	*p* value
**1 mg/kg**	**1**	**39**	**1.71 [0.97, 2.46]**	**< 0.00001**
**0.5 mg/kg**	**5**	**204**	**1.14 [0.71, 1.57]**	**< 0.00001**
0.25 mg/kg	2	57	0.90 [-0.18, 1.99]	0.10
0.1 mg/kg	2	54	0.08 [-0.48, 0.64]	0.77

CADSS, clinician administered dissociative states scale.

Supplementary Table 16

Changes in esketamine-related CADSS scores from baseline to each predefined visit time point.

Visit day	Measurement time post-dose	Number of studies	Number of participants	MD (IV, Random, 95% CI)	*p* value
Day 1	**40 minutes**	**8**	**1363**	**7.25 [4.80, 9.70]**	**< 0.00001**
	**1.5 hours**	**5**	**970**	**0.52 [0.02, 1.01]**	**0.04**
	2 hours	2	107	0.44 [-0.05, 0.94]	0.08
	4 hours	1	29	0.31 [-0.57, 1.19]	0.49
Day 4	**40 minutes**	**6**	**1015**	**5.27 [4.14, 6.41]**	**< 0.00001**
	**1.5 hours**	**4**	**914**	**0.78 [0.41, 1.15]**	**< 0.0001**
	2 hours	2	102	0.14 [-0.26, 0.55]	0.49
Day 8	**40 minutes**	**5**	**961**	**5.21 [4.51, 5.91]**	**< 0.00001**
	**1.5 hours**	**4**	**902**	**0.78 [0.40, 1.17]**	**< 0.0001**
	2 hours	1	59	0.14 [-0.46, 0.74]	0.65
Day 11	**40 minutes**	**4**	**685**	**4.20 [3.47, 4.93]**	**< 0.00001**
	**1.5 hours**	**3**	**634**	**0.61 [0.20, 1.02]**	**0.003**
	2 hours	1	51	-0.13 [-0.79, 0.53]	0.70
Day 15	**40 minutes**	**5**	**937**	**4.08 [3.53, 4.63]**	**< 0.00001**

**Visit day**	**Measurement time post-dose**	**Number of studies**	**Number of participants**	**MD (IV, Random, 95% CI)**	***p* value**
	**1.5 hours**	**4**	**882**	**0.45 [0.11, 0.78]**	**0.009**
	2 hours	1	54	0.04 [-0.41, 0.49]	0.86
Day 18	**40 minutes**	**4**	**659**	**3.98 [3.09, 4.86]**	**< 0.00001**
	**1.5 hours**	**3**	**608**	**0.41 [0.03, 0.79]**	**0.03**
	2 hours	1	49	0.36 [-0.62, 1.34]	0.47
Day 22	**40 minutes**	**5**	**921**	**3.53 [2.94, 4.12]**	**< 0.00001**
	**1.5 hours**	**4**	**867**	**0.43 [0.10, 0.76]**	**0.01**
	2 hours	1	51	0.11 [-0.35, 0.57]	0.64
Day 25	**40 minutes**	**5**	**898**	**3.13 [2.54, 3.72]**	**< 0.00001**
	1.5 hours	4	851	0.30 [-0.00, 0.61]	0.05
	2 hours	1	46	0.65 [-0.03, 1.33]	0.06

CADSS, clinician administered dissociative states scale.

Supplementary Table 17

Subgroup analyses of esketamine dose on changes in CADSS scores at 40 minutes post-dose at each visit time point.

**Visit day**	**Esketamine dose**	**Number of Studies**	**Number of Participants**	**MD (IV, Random, 95% CI)**	***p* value**
Day 1	**84 mg**	**3**	**310**	**8.45 [5.32, 11.57]**	**< 0.00001**
	**56 mg**	**2**	**245**	**6.49 [5.00, 7.98]**	**< 0.00001**
	28 mg	1	18	2.56 [-0.09, 5.21]	0.06
Day 4	**84 mg**	**3**	**293**	**7.28 [5.46, 9.09]**	**< 0.00001**
	**56 mg**	**2**	**242**	**4.16 [2.92, 5.41]**	**< 0.00001**
	28 mg	1	18	1.15 [-0.26, 2.56]	0.11
Day 8	**84 mg**	**2**	**266**	**5.97 [4.33, 7.61]**	**< 0.00001**
	**56 mg**	**1**	**216**	**3.74 [2.47, 5.01]**	**< 0.00001**
Day 11	**84 mg**	**2**	**251**	**5.15 [3.86, 6.44]**	**< 0.00001**
	**56 mg**	**1**	**213**	**2.64 [1.55, 3.73]**	**< 0.00001**
Day 15	**84 mg**	**2**	**258**	**4.38 [3.21, 5.55]**	**< 0.00001**
	**56 mg**	**1**	**211**	**3.57 [2.52, 4.62]**	**< 0.00001**
Day 18	**84 mg**	**2**	**244**	**4.23 [3.14, 5.33]**	**< 0.00001**
	**56 mg**	**1**	**208**	**2.62 [1.76, 3.48]**	**< 0.00001**
Day 22	**84 mg**	**2**	**247**	**4.44 [3.09, 5.80]**	**< 0.00001**
	**56 mg**	**1**	**206**	**2.54 [1.62, 3.46]**	**< 0.00001**
Day 25	**84 mg**	**2**	**240**	**4.60 [3.05, 6.16]**	**< 0.00001**
	**56 mg**	**1**	**204**	**1.96 [1.03, 2.89]**	**< 0.0001**

CADSS, clinician administered dissociative states scale.

Supplementary Table 18

Changes in ketamine-related BPRS+ scores from baseline to each predefined time point.

Measurement time post-dose	Number of Studies	Number of Participants	MD (IV, Random, 95% CI)	*p* value
**10 minutes**	**1**	**7**	**4.30 [0.97, 7.63]**	**0.01**
**40 minutes**	**2**	**25**	**3.46 [2.38, 4.53]**	**< 0.00001**
**60 minutes**	**2**	**231**	**0.80 [0.40, 1.20]**	**< 0.0001**
80 minutes	2	25	0.57 [-0.77, 1.90]	0.40
110 minutes	2	25	-0.14 [-0.94, 0.66]	0.73
120 minutes	1	174	0.06 [-0.25, 0.37]	0.70
240 minutes	3	197	0.06 [-0.31, 0.43]	0.76
24 hours	2	189	-0.03 [-0.54, 0.49]	0.92

BPRS+, brief psychiatric rating scale positive symptom subscale.

Supplementary Table 19

Ketamine-related changes in SBP and DBP from baseline to each predefined time point.

Outcomes	Measurement time post-dose	Number of Studies	Number of Participants	MD (IV, Random, 95% CI)	*p* value
Increase in SBP	**15 minutes**	**1**	**174**	**10.55 [6.42, 14.68]**	**< 0.00001**
	**40 minutes**	**6**	**316**	**13.64 [7.96, 19.32]**	**< 0.00001**
	**60 minutes**	**1**	**174**	**8.78 [4.80, 12.76]**	**< 0.0001**
	120 minutes	2	245	0.18 [-2.30, 2.67]	0.89
	240 minutes	2	110	2.85 [-12.54, 18.23]	0.72
	1 week	1	27	4.33 [-7.20, 15.86]	0.46
	2 weeks	1	27	-3.09 [-16.62, 10.44]	0.65
Increase in DBP	**15 minutes**	**1**	**174**	**6.58 [4.00, 9.16]**	**< 0.00001**
	**40 minutes**	**6**	**316**	**8.97 [4.93, 13.01]**	**< 0.00001**
	**60 minutes**	**1**	**174**	**5.96 [3.42, 8.50]**	**< 0.00001**
	120 minutes	2	245	1.82 [-1.91, 5.56]	0.34
	240 minutes	2	110	0.62 [-3.07, 4.31]	0.74
	1 week	1	27	-3.51 [-12.10, 5.08]	0.42
	2 weeks	1	27	-6.05 [-15.46, 3.36]	0.21

SBP, systolic blood pressure; DBP, diastolic blood pressure.

Supplementary Table 20

Meta-analyses for the number of participants with treatment-emergent SBP or DBP abnormalities after ketamine administration.

Outcomes	Number of Studies	Number of Participants	Risk Ratio (M-H, Random, 95% CI)	*p* value
**SBP above 150 mmHg**	**4**	**312**	**7.84 [2.24, 27.46]**	**0.001**
**SBP above 180 mmHg**	**2**	**208**	**8.33 [1.54, 44.95]**	**0.01**
**Increases in SBP > 20 mmHg**	**3**	**234**	**4.01 [1.22, 13.14]**	**0.02**
**DBP above 99 mmHg**	**3**	**307**	**7.34 [1.73, 31.08]**	**0.007**
**DBP above 110 mmHg**	**2**	**208**	**8.33 [1.54, 44.95]**	**0.01**
**Increases in DBP > 15 mmHg**	**2**	**208**	**8.33 [1.54, 44.95]**	**0.01**

SBP, systolic blood pressure; DBP, diastolic blood pressure.

Supplementary Table 21

Esketamine-related changes in SBP and DBP from baseline to each predefined visit time point.

**Outcomes**	**Dosing day**	**Measurement time post-dose**	**Number of Studies**	**Number of Participants**	**MD (IV, Random, 95% CI)**	***p* value**
Increase in SBP	Day 1	**40 minutes**	**9**	**1815**	**9.24 [7.92, 10.56]**	**< 0.00001**
		**60 minutes**	**1**	**252**	**6.18 [2.17, 10.19]**	**0.003**
		**1.5 hours**	**7**	**1706**	**3.68 [2.36, 5.00]**	**< 0.00001**
		2 hours	1	41	3.05 [-6.82, 12.92]	0.54
	Day 4	**40 minutes**	**7**	**1385**	**9.22 [7.70, 10.74]**	**< 0.00001**
		**60 minutes**	**1**	**252**	**5.38 [1.70, 9.06]**	**0.004**
		**1.5 hours**	**6**	**1344**	**4.45 [2.57, 6.33]**	**< 0.00001**
		2 hours	1	41	5.76 [-5.55, 17.07]	0.32
	Day 8	**40 minutes**	**6**	**1324**	**8.69 [7.15, 10.24]**	**< 0.00001**
		**60 minutes**	**1**	**252**	**5.69 [1.69, 9.69]**	**0.005**
		**1.5 hours**	**6**	**1324**	**2.83 [1.40, 4.27]**	**0.0001**
	Day 11	**40 minutes**	**6**	**1277**	**8.89 [7.33, 10.44]**	**< 0.00001**
		**60 minutes**	**1**	**252**	**6.17 [2.58, 9.76]**	**0.0007**
		**1.5 hours**	**6**	**1278**	**3.14 [1.60, 4.67]**	**< 0.0001**
	Day 15	**40 minutes**	**6**	**1276**	**9.24 [7.64, 10.84]**	**< 0.00001**
		**60 minutes**	**1**	**252**	**7.27 [3.63, 10.91]**	**< 0.0001**
		**1.5 hours**	**6**	**1277**	**3.60 [2.14, 5.06]**	**< 0.00001**
	Day 18	**40 minutes**	**6**	**1229**	**8.16 [6.67, 9.65]**	**< 0.00001**
		**60 minutes**	**1**	**252**	**5.70 [1.75, 9.65]**	**0.005**
		**1.5 hours**	**6**	**1229**	**2.10 [0.58, 3.61]**	**0.007**
	Day 22	**40 minutes**	**6**	**1252**	**7.60 [6.03, 9.17]**	**< 0.00001**
		**60 minutes**	**1**	**252**	**5.22 [1.43, 9.01]**	**0.007**
		**1.5 hours**	**6**	**1251**	**3.00 [1.48, 4.51]**	**0.0001**
	Day 25	**40 minutes**	**6**	**1217**	**8.12 [6.52, 9.71]**	**< 0.00001**
		**60 minutes**	**1**	**252**	**6.49 [3.00, 9.98]**	**0.0003**
		**1.5 hours**	**6**	**1215**	**3.02 [1.48, 4.55]**	**0.0001**
Increase in DBP	Day 1	**40 minutes**	**8**	**1672**	**6.00 [4.96, 7.04]**	**< 0.00001**
		**60 minutes**	**1**	**252**	**4.90 [2.37, 7.43]**	**0.0001**
		**1.5 hours**	**6**	**1564**	**2.44 [1.29, 3.59]**	**< 0.0001**
		2 hours	1	41	-2.63 [-9.90, 4.64]	0.48
	Day 4	**40 minutes**	**6**	**1251**	**6.90 [5.78, 8.01]**	**< 0.00001**
		**60 minutes**	**1**	**252**	**4.76 [1.97, 7.55]**	**0.0008**
		**1.5 hours**	**5**	**1210**	**3.65 [2.43, 4.88]**	**< 0.00001**
		2 hours	1	41	0.35 [-5.71, 6.41]	0.91
	Day 8	**40 minutes**	**5**	**1197**	**5.46 [4.37, 6.55]**	**< 0.00001**
		**60 minutes**	**1**	**252**	**3.88 [1.44, 6.32]**	**0.002**
		**1.5 hours**	**5**	**1197**	**1.74 [0.65, 2.83]**	**0.002**
	Day 11	**40 minutes**	**5**	**1152**	**5.43 [4.30, 6.56]**	**< 0.00001**
		**60 minutes**	**1**	**252**	**2.96 [0.48, 5.44]**	**0.02**
		**1.5 hours**	**5**	**1152**	**1.89 [0.78, 3.00]**	**0.0009**

Outcomes	Dosing day	Measurement time post-dose	Number of Studies	Number of Participants	MD (IV, Random, 95% CI)	*p* value
	Day 15	**40 minutes**	**5**	**1158**	**5.88 [4.77, 7.00]**	**< 0.00001**
		**60 minutes**	**1**	**252**	**4.40 [1.85, 6.95]**	**0.0007**
		**1.5 hours**	**5**	**1159**	**2.46 [1.42, 3.50]**	**< 0.0001**
	Day 18	**40 minutes**	**5**	**1111**	**5.87 [4.32, 7.42]**	**< 0.00001**
		**60 minutes**	**1**	**252**	**3.90 [1.19, 6.61]**	**0.005**
		**1.5 hours**	**5**	**1111**	**2.51 [1.41, 3.61]**	**< 0.0001**
	Day 22	**40 minutes**	**5**	**1136**	**5.53 [4.41, 6.64]**	**< 0.00001**
		**60 minutes**	**1**	**252**	**3.60 [1.19, 6.01]**	**0.003**
		**1.5 hours**	**5**	**1135**	**2.66 [1.63, 3.69]**	**< 0.0001**
	Day 25	**40 minutes**	**5**	**1101**	**5.64 [4.53, 6.75]**	**< 0.00001**
		**60 minutes**	**1**	**252**	**5.83 [3.47, 8.19]**	**< 0.00001**
		**1.5 hours**	**5**	**1099**	**2.26 [1.02, 3.49]**	**0.0004**

SBP, systolic blood pressure; DBP, diastolic blood pressure.

Supplementary Table 22

Meta-analyses for the number of participants with treatment-emergent SBP or DBP abnormalities after esketamine administration.

Outcomes	Number of Studies	Number of Participants	Risk Ratio (M-H, Random, 95% CI)	*p* value
SBP above 180 mmHg	2	434	2.13 [0.32, 14.32]	0.44
Increases in SBP > 20 mmHg	3	563	2.09 [0.85, 5.16]	0.11
DBP above 110 mmHg	2	434	2.40 [0.72, 7.98]	0.15
Increases in DBP > 15 mmHg	3	563	2.47 [0.47, 12.89]	0.28

SBP, systolic blood pressure; DBP, diastolic blood pressure.

Supplementary Table 23

Meta-analyses for the number of participants with treatment-emergent abnormal ECG values after esketamine administration.

Outcomes	Number of Studies	Number of Participants	Risk Ratio (M-H, Random, 95% CI)	*p* value
Abnormally high pulse rate interval	2	450	1.39 [0.45, 4.36]	0.57
Abnormally high QRS interval	1	225	0.34 [0.01, 8.17]	0.50

Abnormally high pulse rate interval and QRS interval are defined as ≥ 210 and ≥ 120 milliseconds, respectively. ECG, electrocardiography.

Supplementary Table 24

Meta-analyses for the effects of ketamine on cognitive performance.

Visit day	Measurements	Outcomes	Number of Studies	Number of Participants	MD (IV, Random, 95% CI)	*p* value
Day 1	Neuropsycholigical measures	Choice reaction time	1	78	0.44 [-0.21, 1.09]	0.19
		Digit symbol	1	78	-0.05 [-0.52, 0.42]	0.83
		Continuous performance test	1	78	-0.04 [-0.51, 0.43]	0.87
		Stroop interference	1	78	0.39 [-0.09, 0.87]	0.11
		Buschke SRT immediate	1	78	0.00 [-0.60, 0.60]	1.00
		**Buschke SRT delayed**	**1**	**78**	**1.03 [0.18, 1.88]**	**0.02**
		A Not B reaction	1	78	-0.25 [-0.78, 0.28]	0.35
		Letter fluency	1	78	0.15 [-0.33, 0.63]	0.54
		Category fluency	1	78	0.14 [-0.31, 0.59]	0.54
		Go-No Go Commission Error	1	78	-0.02 [-0.48, 0.44]	0.93
Day 7	MCCB	Working memory (WMS-III Spatial span, letter-number)	1	62	2.13 [-3.53, 7.79]	0.46
		Verbal learning (HVLT learning and delay)	1	62	0.20 [-5.91, 6.31]	0.95
		Processing speed (category fluency, Trails A, BACS Digit symbol)	1	62	-1.94 [-7.39, 3.51]	0.49
		Visual learning (BVMT learning)	1	62	1.75 [-5.02, 8.52]	0.61
		Reasoning/Problem solving (NAB Mazes)	1	62	3.12 [-1.67, 7.91]	0.20
Day 28	CogState computerised-battery	Detection - Attention (simple reaction time)	1	5	0.01 [-2.03, 2.05]	0.99
		Identification - Attention (choice reaction time)	1	5	-0.53 [-3.66, 2.60]	0.74
		One card learning - visual learning	1	5	-1.18 [-3.00, 0.64]	0.20
		2 back-working memory	1	5	1.57 [-2.21, 5.35]	0.42
		**Set shifting task- executive function**	**1**	**5**	**-1.28 [-2.56, -0.00]**	**0.05**
		International shopping list task	1	5	-0.88 [-2.55, 0.79]	0.30

Buschke SRT, Buschke selective reminding test; MCCB, MATRICS consensus cognitive battery; HVLT, Hopkins verbal learning test.

Supplementary Table 25

Meta-analyses for the effects of esketamine on cognitive performance.

**Visit day**	**Outcomes**	**Number of Studies**	**Number of Participants**	**MD (IV, Random, 95% CI)**	***p* value**
Day 1	**MGH-CPFQ**	**1**	**30**	**-5.65 [-10.27, -1.03]**	**0.02**
Day 28	Detection-attention (simple reaction time)	1	137	0.01 [-0.04, 0.05]	0.79
	Identification - attention (choice reaction time)	1	137	0.01 [-0.01, 0.04]	0.26
	One card learning - visual learning	1	137	-0.02 [-0.06, 0.02]	0.26
	One back - working memory	1	137	-0.02 [-0.05, 0.00]	0.11
	Groton maze learning test - executive function	1	137	5.30 [-1.14, 11.74]	0.11
	HVLT-R: Total recall	1	137	1.30 [-0.30, 2.90]	0.11
	HVLT-R: Delayed recall	1	137	0.10 [-0.72, 0.92]	0.81
Month 1	**MoCA**	**1**	**236**	**1.12 [0.67, 1.57]**	**< 0.00001**
Month 2	**MoCA**	**1**	**236**	**1.70 [1.22, 2.18]**	**< 0.00001**
Month 8	**MoCA**	**1**	**236**	**2.01 [1.55, 2.47]**	**< 0.00001**

HVLT-R, Hopkins verbal learning test-revised; MGH-CPFQ, Massachusetts general hospital-cognitive and physical functioning questionnaire; MoCA, Montreal cognitive assessment.

Supplementary Table 26

The potential withdrawal symptoms after cessation of esketamine treatment.

Follow-up phase	Symptoms of new or worsened	Number of Studies	Number of Participants	Risk Ratio (M-H, Random, 95% CI)	*p* value
Week 1	Dysphoric Mood/Depression	1	65	0.93 [0.40, 2.20]	0.87
	**Fatigue/Lethargy/Lack of energy**	**1**	**65**	**28.56 [1.72, 473.84]**	**0.02**
	Insomnia	1	65	0.07 [0.00, 1.09]	0.06
	Irritability	1	65	0.07 [0.00, 1.19]	0.07
	**Loss of appetite**	**1**	**65**	**21.84 [1.28, 371.39]**	**0.03**
	**Restlessness/Agitation**	**1**	**65**	**21.84 [1.28, 371.39]**	**0.03**
	**Weakness**	**1**	**65**	**25.20 [1.50, 422.60]**	**0.02**
Week 2	Anxiety/Nervousness	2	62	5.38 [0.43, 67.26]	0.19
	Diaphoresis	1	47	0.10 [0.01, 1.65]	0.11
	Difficulty concentrating/Remembering	2	62	0.39 [0.01, 13.23]	0.60
	Dizziness/Lightheadedness	1	15	0.06 [0.00, 1.03]	0.05
	Dysphoric mood/Depression	1	47	0.10 [0.01, 1.65]	0.11
	**Fatigue/Lethargy/Lack of energy**	**1**	**47**	**22.39 [1.34, 374.56]**	**0.03**
	Insomnia	1	47	0.06 [0.00, 1.01]	0.05
	Loss of appetite	2	62	0.39 [0.02, 6.35]	0.51
	Muscle aches or stiffness	1	47	0.07 [0.00, 1.20]	0.07
	Nausea/Vomiting	1	15	1.54 [0.09, 25.86]	0.76
	Poor coordination and irritability	1	47	0.09 [0.01, 1.47]	0.09
	**Restlessness/Agitation**	**1**	**47**	**18.94 [1.11, 323.02]**	**0.04**
	Weakness	1	47	0.10 [0.01, 1.65]	0.11

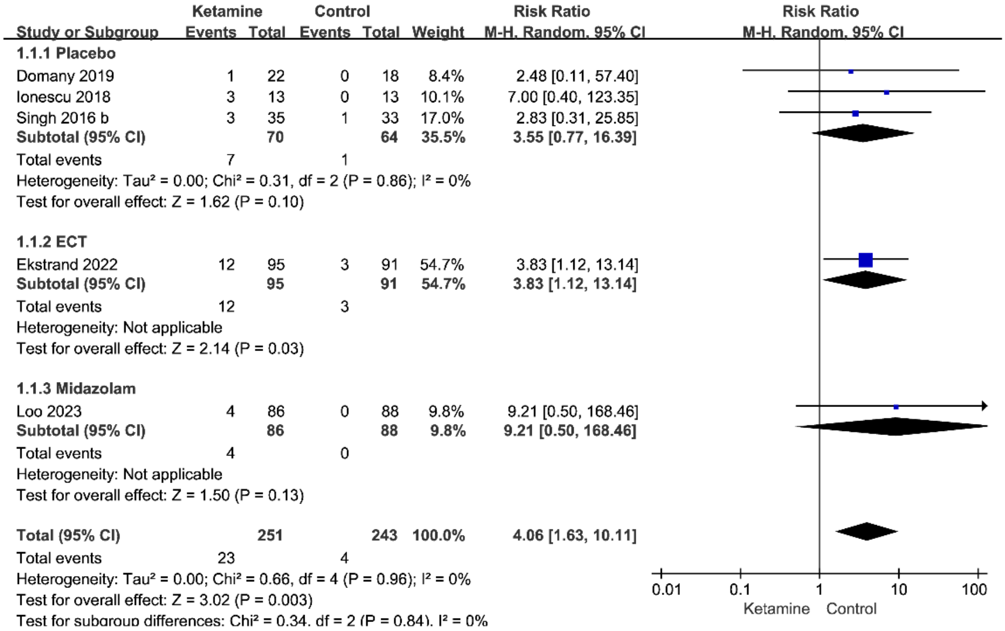

Supplementary Figure 1. Forest plot of comparisons between ketamine and controls for the dropout rate due to AEs.

Data from subgroup analyses are also presented by dividing controls into placebo, midazolam, and ECT. AEs, adverse events; ECT, electroconvulsive therapy.

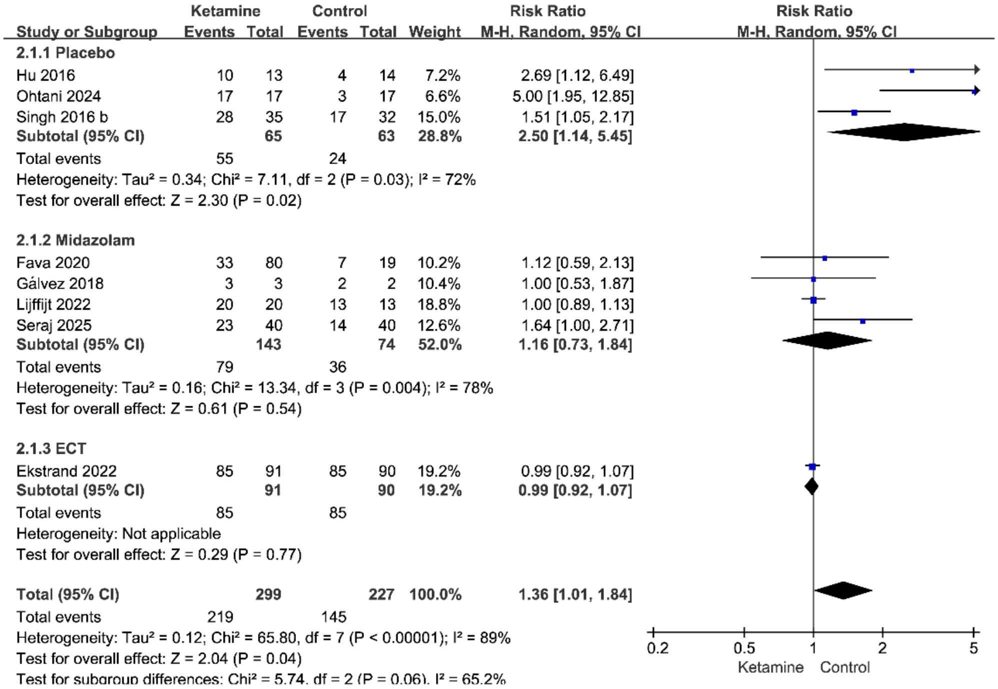

Supplementary Figure 2. Forest plot of comparisons between ketamine and controls for the number of participants experiencing at least one AE.

Data from subgroup analyses are also presented by dividing controls into placebo, midazolam, and ECT. AE, adverse event; ECT, electroconvulsive therapy.

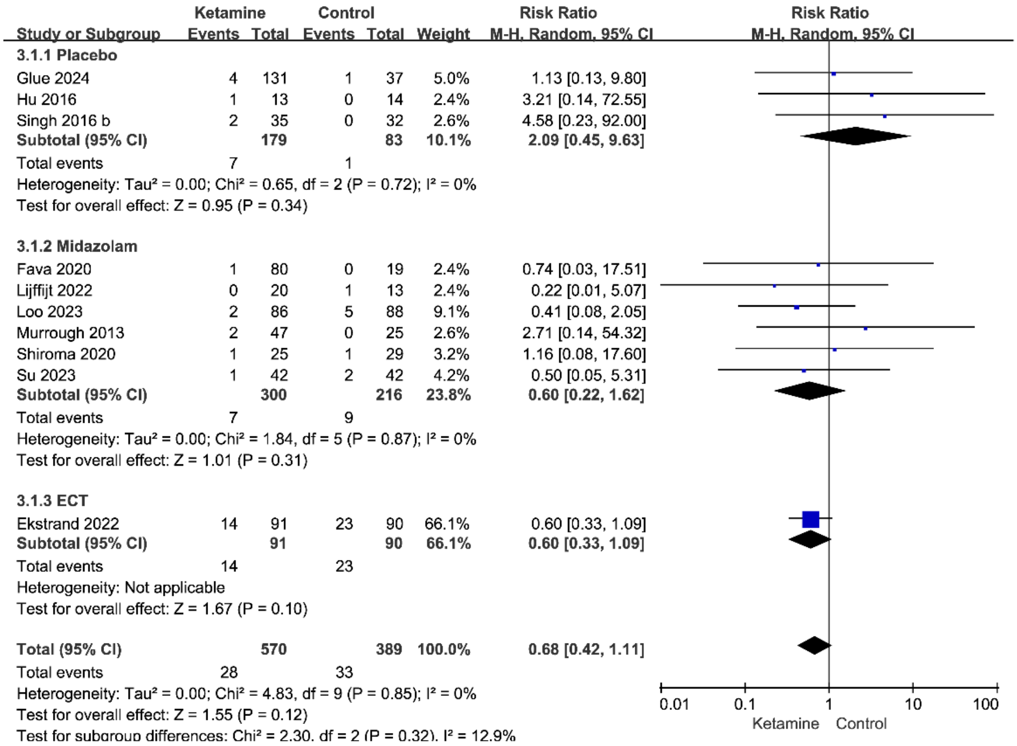

Supplementary Figure 3. Forest plot of comparisons between ketamine and controls for the number of participants experiencing at least one serious AE.

Data from subgroup analyses are also presented by dividing controls into placebo, midazolam, and ECT. AE, adverse event; ECT, electroconvulsive therapy.

Study or Subgroup	Esketamine Events	Esketamine Total	Control Events	Control Total	Weight	Risk Ratio M-H, Random, 95% CI
**4.1.1 Placebo**						
Canuso 2018	5	35	1	31	5.4%	4.43 [0.55, 35.87]
Chen 2023	7	126	2	126	7.7%	3.50 [0.74, 16.52]
Daly 2018	3	56	0	33	3.3%	4.18 [0.22, 78.40]
Daly 2019	4	152	3	145	8.0%	1.27 [0.29, 5.59]
Fedgchin 2019	8	233	2	113	7.8%	1.94 [0.42, 8.99]
Fu 2020	5	113	5	112	9.5%	0.99 [0.30, 3.33]
Hong 2025	4	118	0	118	3.4%	9.00 [0.49, 165.32]
Ionescu 2021	9	114	3	113	9.1%	2.97 [0.83, 10.70]
Ochs-Ross 2020	4	72	2	65	7.1%	1.81 [0.34, 9.53]
Popova 2019	8	115	1	109	5.5%	7.58 [0.96, 59.63]
Singh 2016 a	1	42	0	10	3.0%	0.77 [0.03, 17.58]
Smith-Apeldoorn 2024	7	57	2	54	7.8%	3.32 [0.72, 15.26]
Takahashi 2021	7	122	3	80	8.9%	1.53 [0.41, 5.74]
**Subtotal (95% CI)**		**1355**		**1109**	**86.6%**	**2.21 [1.39, 3.49]**
Total events	72		24			
Heterogeneity: Tau² = 0.00; Chi² = 6.87, df = 12 (P = 0.87); I² = 0%						
Test for overall effect: Z = 3.38 (P = 0.0007)						
**4.1.2 Quetiapine**						
Reif 2023	14	334	37	336	13.4%	0.38 [0.21, 0.69]
**Subtotal (95% CI)**		**334**		**336**	**13.4%**	**0.38 [0.21, 0.69]**
Total events	14		37			
Heterogeneity: Not applicable						
Test for overall effect: Z = 3.18 (P = 0.001)						
**Total (95% CI)**		**1689**		**1445**	**100.0%**	**1.85 [1.01, 3.40]**
Total events	86		61			
Heterogeneity: Tau² = 0.62; Chi² = 27.88, df = 13 (P = 0.009); I² = 53%						
Test for overall effect: Z = 1.99 (P = 0.05)						
Test for subgroup differences: Chi² = 20.95, df = 1 (P < 0.00001), I² = 95.2%						

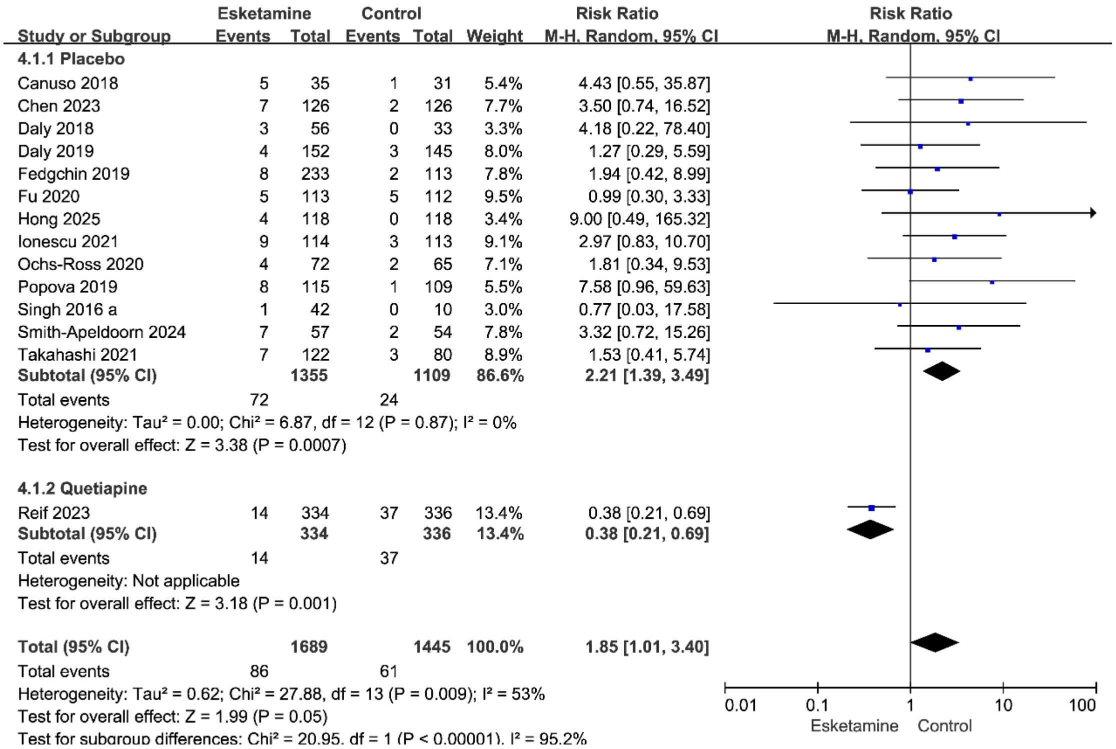

Supplementary Figure 4. Forest plot of comparisons between esketamine and controls for the dropout rate due to AEs.

Data from subgroup analyses are also presented by dividing controls into placebo and quetiapine. AEs, adverse events.

Study or Subgroup	Esketamine Events	Esketamine Total	Control Events	Control Total	Weight	Risk Ratio M-H, Random, 95% CI
**5.1.1 Placebo**						
Canuso 2018	33	35	25	31	8.7%	1.17 [0.97, 1.41]
Chen 2023	120	126	89	126	10.7%	1.35 [1.20, 1.52]
Daly 2018	42	56	18	33	5.0%	1.38 [0.97, 1.94]
Daly 2019	114	152	45	145	6.8%	2.42 [1.86, 3.13]
Fedgchin 2019	187	231	64	113	9.2%	1.43 [1.20, 1.70]
Fu 2020	100	113	83	112	10.5%	1.19 [1.05, 1.36]
Ionescu 2021	104	114	87	113	10.8%	1.18 [1.06, 1.33]
Ochs-Ross 2020	51	72	39	65	7.1%	1.18 [0.92, 1.51]
Popova 2019	91	114	54	109	8.1%	1.61 [1.31, 1.99]
Singh 2016 a	27	42	5	10	2.0%	1.29 [0.66, 2.49]
Takahashi 2021	111	122	51	80	9.1%	1.43 [1.20, 1.70]
**Subtotal (95% CI)**		**1177**		**937**	**88.0%**	**1.38 [1.23, 1.54]**
Total events	980		560			
Heterogeneity: Tau² = 0.02; Chi² = 39.49, df = 10 (P < 0.0001); I² = 75%						
Test for overall effect: Z = 5.52 (P < 0.00001)						
**5.1.2 Quetiapine**						
Reif 2023	307	334	262	336	12.0%	1.18 [1.10, 1.26]
**Subtotal (95% CI)**		**334**		**336**	**12.0%**	**1.18 [1.10, 1.26]**
Total events	307		262			
Heterogeneity: Not applicable						
Test for overall effect: Z = 4.95 (P < 0.00001)						
**Total (95% CI)**		**1511**		**1273**	**100.0%**	**1.35 [1.22, 1.49]**
Total events	1287		822			
Heterogeneity: Tau² = 0.02; Chi² = 49.13, df = 11 (P < 0.00001); I² = 78%						
Test for overall effect: Z = 5.84 (P < 0.00001)						
Test for subgroup differences: Chi² = 5.38, df = 1 (P = 0.02), I² = 81.4%						

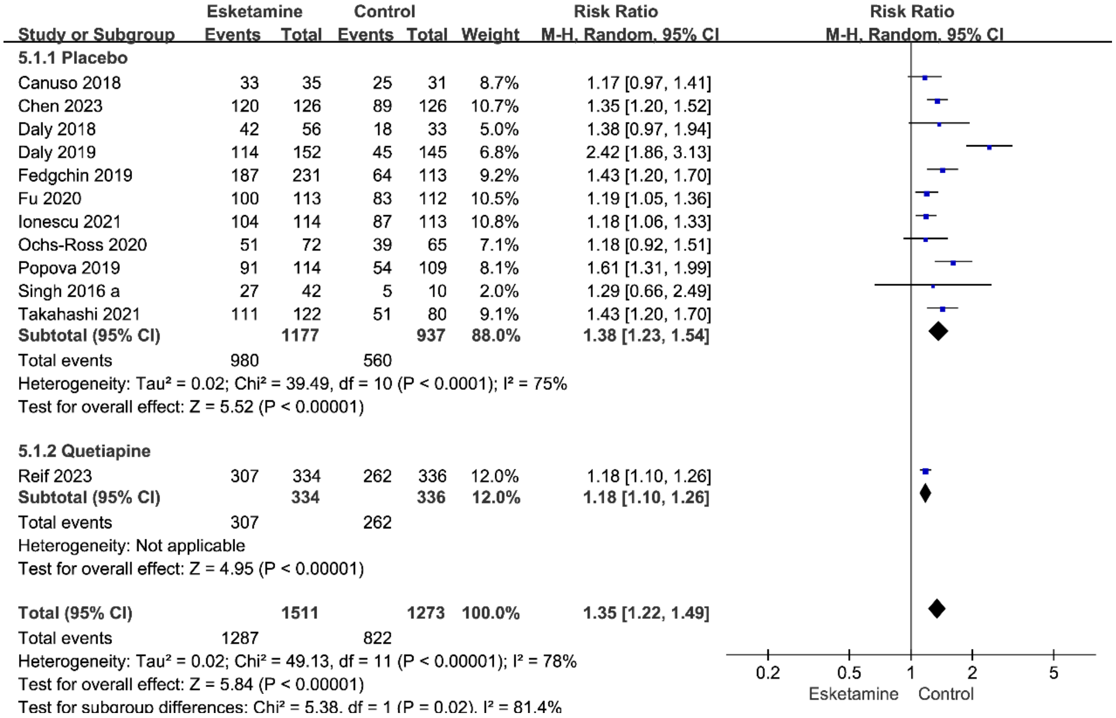

Supplementary Figure 5. Forest plot of comparisons between esketamine and controls for the

number of participants experiencing at least one AE.

Data from subgroup analyses are also presented by dividing controls into placebo and quetiapine. AEs, adverse events.

Study or Subgroup	Esketamine Events	Esketamine Total	Control Events	Control Total	Weight	Risk Ratio M-H, Random, 95% CI
**6.1.1 Placebo**						
Canuso 2018	4	35	0	31	2.2%	8.00 [0.45, 142.90]
Chen 2023	3	126	3	126	7.2%	1.00 [0.21, 4.86]
Daly 2019	4	152	1	145	3.8%	3.82 [0.43, 33.74]
Fedgchin 2019	2	231	0	113	2.0%	2.46 [0.12, 50.75]
Fu 2020	4	113	6	112	11.7%	0.66 [0.19, 2.28]
Ionescu 2021	5	114	6	113	13.3%	0.83 [0.26, 2.63]
Ochs-Ross 2020	3	72	2	65	5.8%	1.35 [0.23, 7.85]
Popova 2019	1	114	1	109	2.3%	0.96 [0.06, 15.10]
Singh 2016 a	3	42	0	10	2.1%	1.79 [0.10, 32.16]
Smith-Apeldoorn 2024	1	57	1	54	2.4%	0.95 [0.06, 14.77]
Takahashi 2021	2	122	1	80	3.1%	1.31 [0.12, 14.22]
**Subtotal (95% CI)**		**1178**		**958**	**55.9%**	**1.14 [0.65, 2.01]**
Total events	32		21			
Heterogeneity: Tau² = 0.00; Chi² = 4.54, df = 10 (P = 0.92); I² = 0%						
Test for overall effect: Z = 0.47 (P = 0.64)						
**6.1.2 Quetiapine**						
Reif 2023	19	334	17	336	44.1%	1.12 [0.59, 2.12]
**Subtotal (95% CI)**		**334**		**336**	**44.1%**	**1.12 [0.59, 2.12]**
Total events	19		17			
Heterogeneity: Not applicable						
Test for overall effect: Z = 0.36 (P = 0.72)						
**Total (95% CI)**		**1512**		**1294**	**100.0%**	**1.14 [0.74, 1.73]**
Total events	51		38			
Heterogeneity: Tau² = 0.00; Chi² = 4.50, df = 11 (P = 0.95); I² = 0%						
Test for overall effect: Z = 0.59 (P = 0.56)						
Test for subgroup differences: Chi² = 0.00, df = 1 (P = 0.97), I² = 0%						

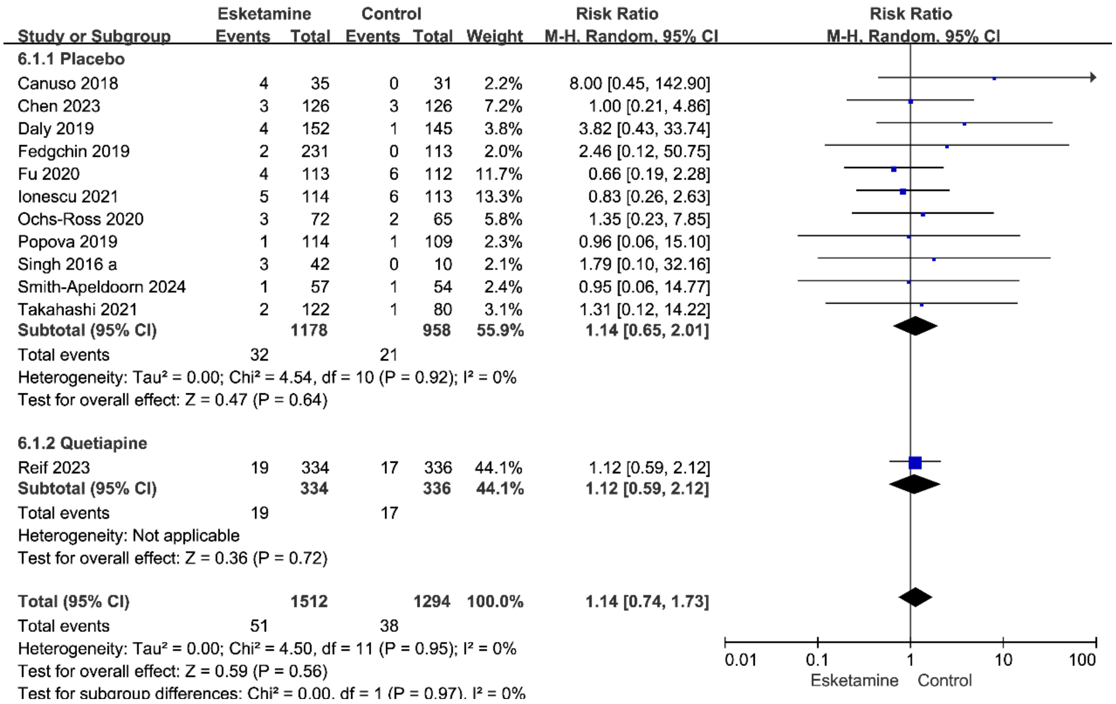

Supplementary Figure 6. Forest plot of comparisons between esketamine and controls for the number of participants experiencing at least one serious AE.

Data from subgroup analyses are also presented by dividing controls into placebo and quetiapine. AEs, adverse events.

Study or Subgroup	Esketamine Mean	Esketamine SD	Esketamine Total	Control Mean	Control SD	Control Total	Weight	Mean Difference IV, Random, 95% CI
**7.1.1 30 minutes post-dose**								
Hong 2025	2.87	1.01	118	0.59	0.6	118	100.0%	2.28 [2.07, 2.49]
**Subtotal (95% CI)**			**118**			**118**	**100.0%**	**2.28 [2.07, 2.49]**
Heterogeneity: Not applicable								
Test for overall effect: Z = 21.08 (P < 0.00001)								
**7.1.2 40 minutes post-dose**								
Singh 2016 a	2.92	3.48	18	0.12	0.53	10	100.0%	2.80 [1.16, 4.44]
**Subtotal (95% CI)**			**18**			**10**	**100.0%**	**2.80 [1.16, 4.44]**
Heterogeneity: Not applicable								
Test for overall effect: Z = 3.34 (P = 0.0008)								
**7.1.3 60 minutes post-dose**								
Hong 2025	3.19	1.08	118	0.5	0.6	118	100.0%	2.69 [2.47, 2.91]
**Subtotal (95% CI)**			**118**			**118**	**100.0%**	**2.69 [2.47, 2.91]**
Heterogeneity: Not applicable								
Test for overall effect: Z = 23.65 (P < 0.00001)								
**7.1.4 90 minutes post-dose**								
Hong 2025	1.6	1.27	118	0.38	0.49	118	100.0%	1.22 [0.97, 1.47]
**Subtotal (95% CI)**			**118**			**118**	**100.0%**	**1.22 [0.97, 1.47]**
Heterogeneity: Not applicable								
Test for overall effect: Z = 9.74 (P < 0.00001)								
**7.1.5 2 hours post-dose**								
Hong 2025	0.78	0.88	118	0.31	0.44	118	53.9%	0.47 [0.29, 0.65]
Singh 2016 a	0.01	0.42	20	0.2	0.58	10	46.1%	-0.19 [-0.59, 0.21]
**Subtotal (95% CI)**			**138**			**128**	**100.0%**	**0.17 [-0.48, 0.81]**
Heterogeneity: Tau² = 0.19; Chi² = 8.60, df = 1 (P = 0.003); I² = 88%								
Test for overall effect: Z = 0.50 (P = 0.61)								

Test for subgroup differences: Chi² = 113.66, df = 4 (P < 0.00001), I² = 96.5%

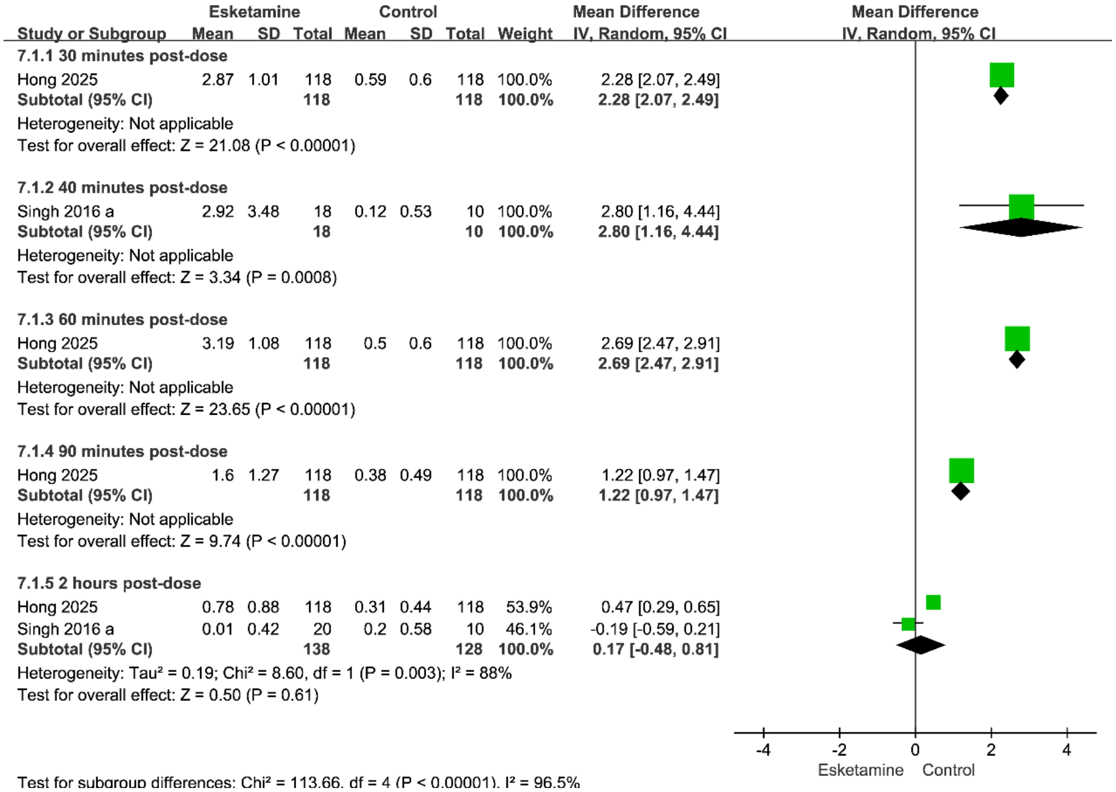

Supplementary Figure 7. Forest plot of changes in esketamine-related BPRS+ scores from baseline to each predefined visit time point.

BPRS+, brief psychiatric rating scale positive symptom subscale.

Study or Subgroup	Ketamine Mean	Ketamine SD	Ketamine Total	Control Mean	Control SD	Control Total	Weight	Mean Difference IV, Random, 95% CI
**8.1.1 40 minutes post-dose**								
Zarate 2006	0.99	2.04	9	-2.2	2.19	9	100.0%	3.19 [1.23, 5.15]
**Subtotal (95% CI)**			**9**			**9**	**100.0%**	**3.19 [1.23, 5.15]**
Heterogeneity: Not applicable								
Test for overall effect: Z = 3.20 (P = 0.001)								
**8.1.2 80 minutes post-dose**								
Zarate 2006	-1.08	2.01	9	-2.22	2.18	9	100.0%	1.14 [-0.80, 3.08]
**Subtotal (95% CI)**			**9**			**9**	**100.0%**	**1.14 [-0.80, 3.08]**
Heterogeneity: Not applicable								
Test for overall effect: Z = 1.15 (P = 0.25)								
**8.1.3 110 minutes post-dose**								
Zarate 2006	-1.64	1.98	9	-2.05	2.11	9	100.0%	0.41 [-1.48, 2.30]
**Subtotal (95% CI)**			**9**			**9**	**100.0%**	**0.41 [-1.48, 2.30]**
Heterogeneity: Not applicable								
Test for overall effect: Z = 0.43 (P = 0.67)								
**8.1.4 230 minutes post-dose**								
Zarate 2006	-1.51	2.04	9	-2.27	2.05	9	100.0%	0.76 [-1.13, 2.65]
**Subtotal (95% CI)**			**9**			**9**	**100.0%**	**0.76 [-1.13, 2.65]**
Heterogeneity: Not applicable								
Test for overall effect: Z = 0.79 (P = 0.43)								
**8.1.5 1 day post-dose**								
Zarate 2006	-2.65	2.04	9	-0.89	2.13	9	100.0%	-1.76 [-3.69, 0.17]
**Subtotal (95% CI)**			**9**			**9**	**100.0%**	**-1.76 [-3.69, 0.17]**
Heterogeneity: Not applicable								
Test for overall effect: Z = 1.79 (P = 0.07)								

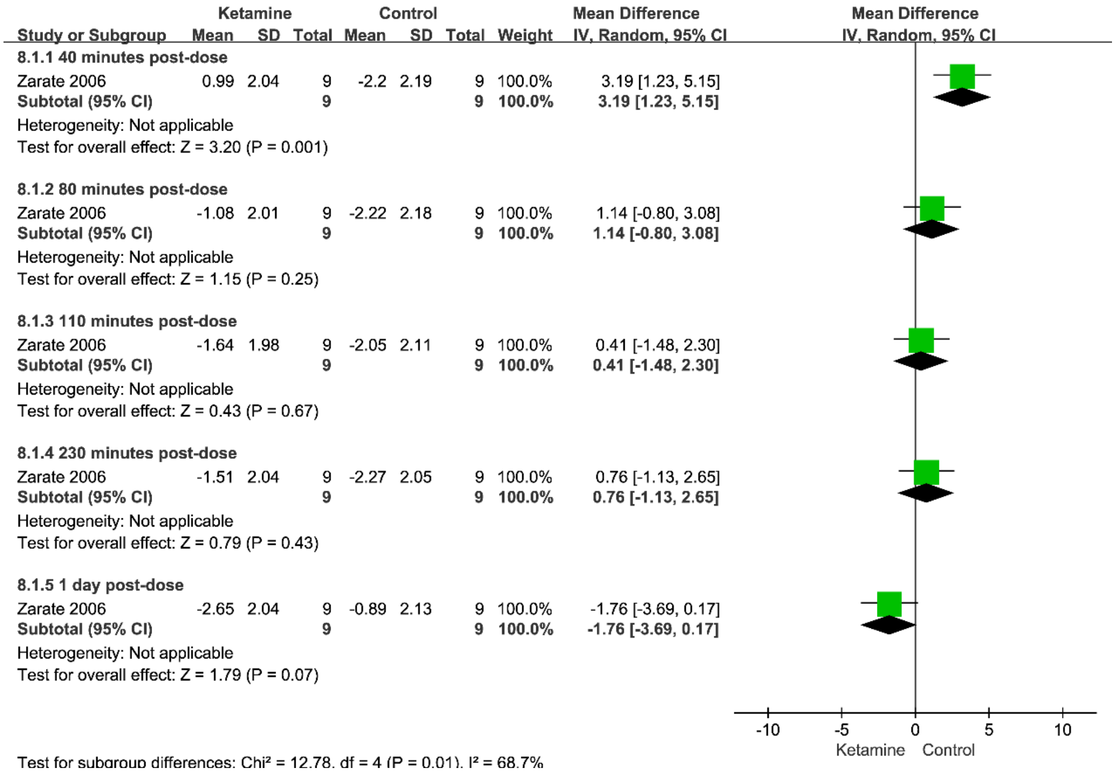

Test for subgroup differences: Chi² = 12.78, df = 4 (P = 0.01), I² = 68.7%

Supplementary Figure 8. Forest plot of changes in ketamine-related YMRS scores from baseline to each predefined visit time point.

YMRS, Young mania rating scale.

Study or Subgroup	Ketamine Mean	Ketamine SD	Ketamine Total	Control Mean	Control SD	Control Total	Weight	Mean Difference IV, Random, 95% CI
**9.1.1 10 minutes post-dose**								
Berman 2000	82.81	22.32	4	0.22	1.76	3	100.0%	82.59 [60.63, 104.55]
**Subtotal (95% CI)**			**4**			**3**	**100.0%**	**82.59 [60.63, 104.55]**
Heterogeneity: Not applicable								
Test for overall effect: Z = 7.37 (P < 0.00001)								
**9.1.2 40 minutes post-dose**								
Berman 2000	44.31	30.12	4	0.44	1.76	3	100.0%	43.87 [14.29, 73.45]
**Subtotal (95% CI)**			**4**			**3**	**100.0%**	**43.87 [14.29, 73.45]**
Heterogeneity: Not applicable								
Test for overall effect: Z = 2.91 (P = 0.004)								
**9.1.3 80 minutes post-dose**								
Berman 2000	12.95	15.62	4	0.22	1.76	3	100.0%	12.73 [-2.71, 28.17]
**Subtotal (95% CI)**			**4**			**3**	**100.0%**	**12.73 [-2.71, 28.17]**
Heterogeneity: Not applicable								
Test for overall effect: Z = 1.62 (P = 0.11)								
**9.1.4 110 minutes post-dose**								
Berman 2000	0.03	0.1	4	0	1.92	3	100.0%	0.03 [-2.14, 2.20]
**Subtotal (95% CI)**			**4**			**3**	**100.0%**	**0.03 [-2.14, 2.20]**
Heterogeneity: Not applicable								
Test for overall effect: Z = 0.03 (P = 0.98)								

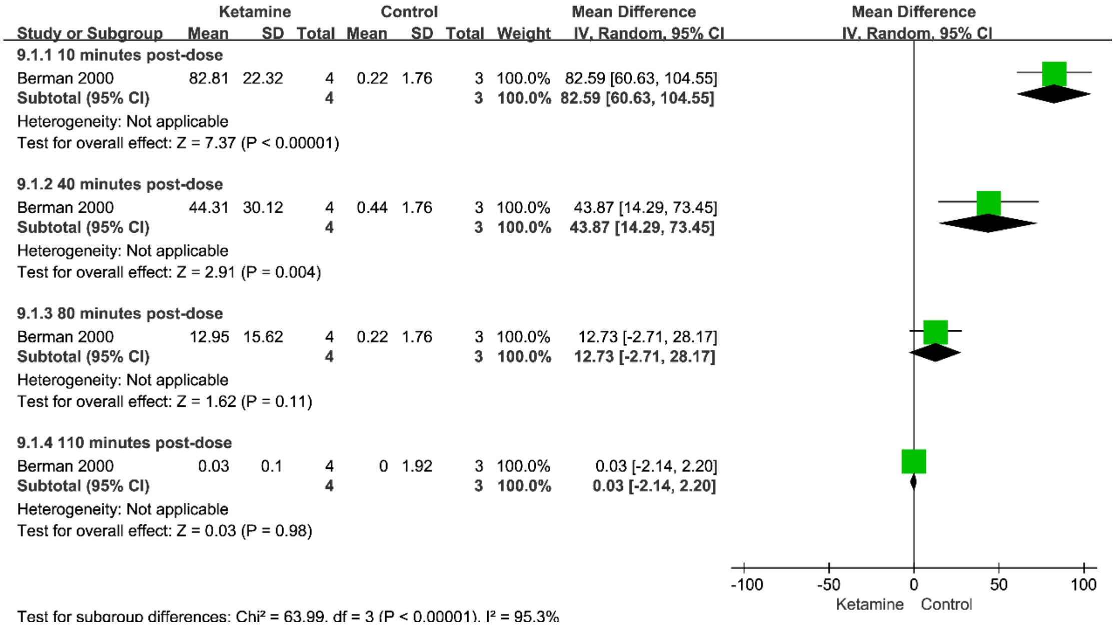

Test for subgroup differences: Chi² = 63.99, df = 3 (P < 0.00001), I² = 95.3%

Supplementary Figure 9. Forest plot of changes in ketamine-related VAS-high scores from baseline to each predefined visit time point.

VAS-high, visual analog scales score for intoxication “high”

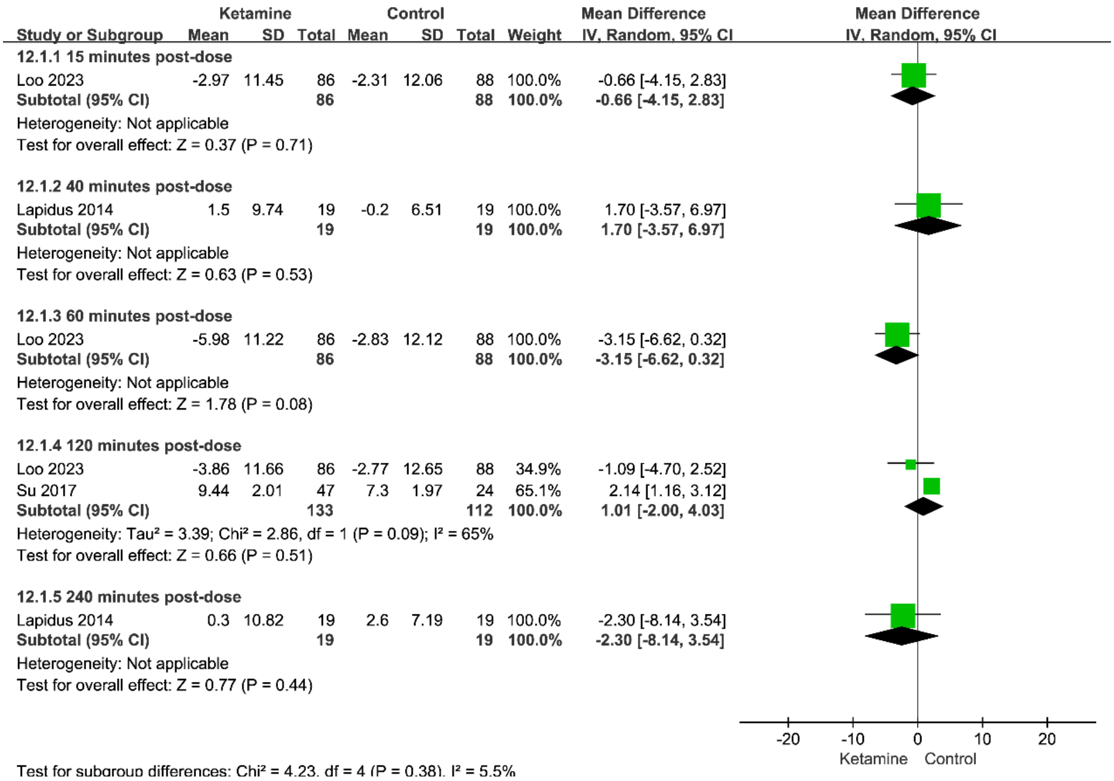

Supplementary Figure 10. Forest plot of ketamine-related changes in heart rate from baseline to each predefined visit time point.

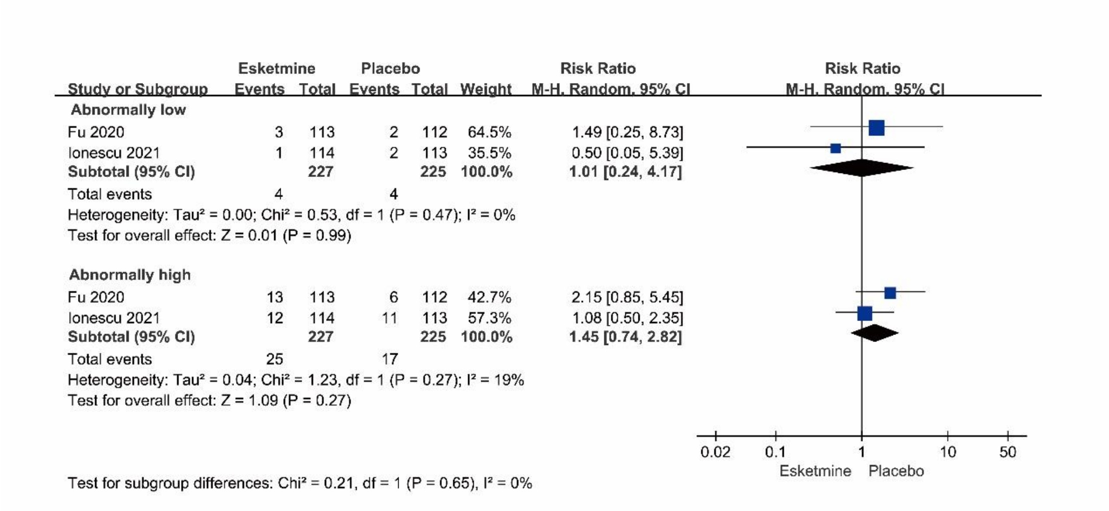

Supplementary Figure 11. Forest plot of the number of participants with treatment-emergent heart rate abnormalities after esketamine administration.

Heart rate abnormalities are classified into abnormally high and abnormally low heart rates. Abnormally low heart rate is defined as a decrease from baseline of ≥ 15 to a value ≤ 50. Abnormally high heart rate is defined as an increase from baseline of ≥ 15 to a value ≥ 100.

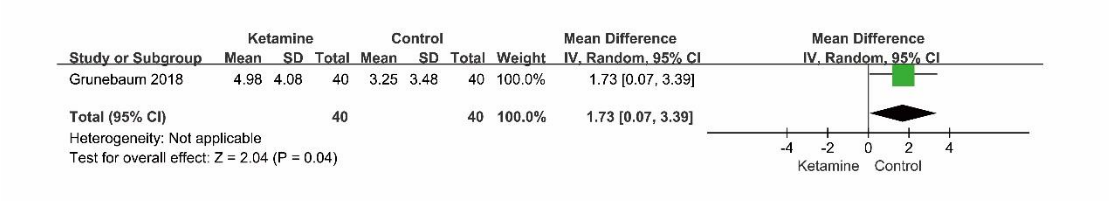

Supplementary Figure 12. Forest plot of ketamine-related changes in respiratory rate at 40 minutes post-dose.

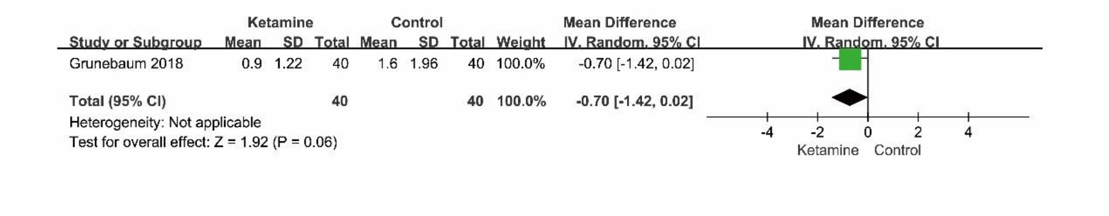

Supplementary Figure 13. Forest plot of ketamine-related changes in oximetry at 40 minutes post-dose.

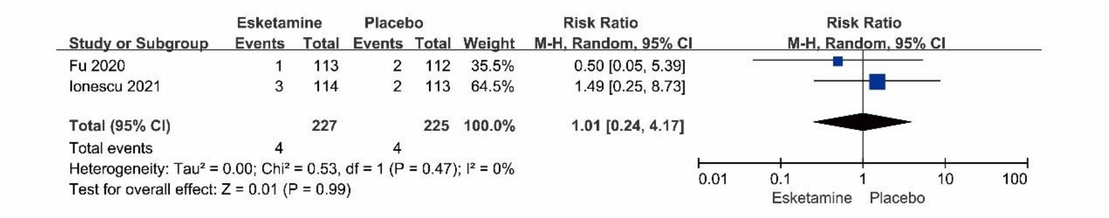

Supplementary Figure 14. Forest plot of the number of participants with abnormally low oximetry after esketamine administration.

Abnormally low oximetry is defined as an arterial oxygen saturation of less than 93%.

Study or Subgroup	Esketamine Events	Esketamine Total	Control Events	Control Total	Weight	Risk Ratio M-H, Random, 95% CI
**17.1.1 MOAA/S score ≤ 4**						
Fu 2020	43	113	20	112	31.8%	2.13 [1.34, 3.38]
Hong 2025	19	118	1	118	6.9%	19.00 [2.59, 139.64]
Ionescu 2021	65	114	20	113	32.8%	3.22 [2.10, 4.94]
Popova 2019	66	115	11	109	28.4%	5.69 [3.18, 10.18]
**Subtotal (95% CI)**		**460**		**452**	**100.0%**	**3.75 [2.11, 6.69]**
Total events	193		52			
Heterogeneity: Tau² = 0.22; Chi² = 10.36, df = 3 (P = 0.02); I² = 71%						
Test for overall effect: Z = 4.49 (P < 0.00001)						
**17.1.2 MOAA/S score ≤ 3**						
Fedgchin 2019	25	231	1	113	16.1%	12.23 [1.68, 89.11]
Fu 2020	13	113	1	112	15.6%	12.88 [1.71, 96.85]
Ionescu 2021	21	114	3	113	45.6%	6.94 [2.13, 22.61]
Ochs-Ross 2020	6	72	1	65	14.6%	5.42 [0.67, 43.81]
Takahashi 2021	14	122	0	80	8.1%	19.10 [1.16, 315.69]
**Subtotal (95% CI)**		**652**		**483**	**100.0%**	**8.77 [3.95, 19.47]**
Total events	79		6			
Heterogeneity: Tau² = 0.00; Chi² = 0.95, df = 4 (P = 0.92); I² = 0%						
Test for overall effect: Z = 5.33 (P < 0.00001)						
**17.1.3 MOAA/S score ≤ 2**						
Fu 2020	3	113	0	112	34.7%	6.94 [0.36, 132.80]
Ionescu 2021	4	114	0	113	35.7%	8.92 [0.49, 163.81]
Popova 2019	1	115	0	109	29.7%	2.84 [0.12, 69.09]
**Subtotal (95% CI)**		**342**		**334**	**100.0%**	**5.82 [1.02, 33.11]**
Total events	8		0			
Heterogeneity: Tau² = 0.00; Chi² = 0.30, df = 2 (P = 0.86); I² = 0%						
Test for overall effect: Z = 1.99 (P = 0.05)						

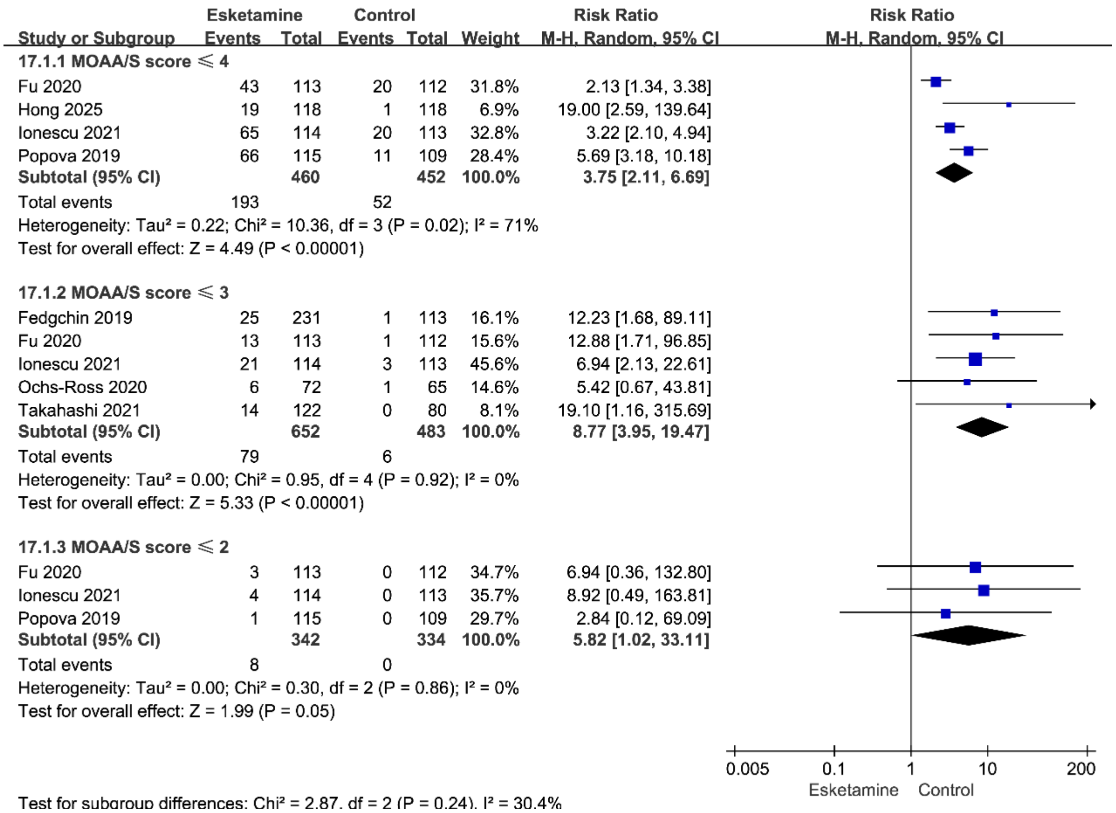

Test for subgroup differences: Chi² = 2.87, df = 2 (P = 0.24), I² = 30.4%

Supplementary Figure 15. Forest plot of the number of participants with different sedation severity after esketamine administration.

Sedation severity is classified as any sedation for a MOAA/S score ≤ 4, moderate or greater sedation for a score ≤ 3, and severe sedation for a score ≤ 2. MOAA/S, modified observer's assessment of alertness/sedation.

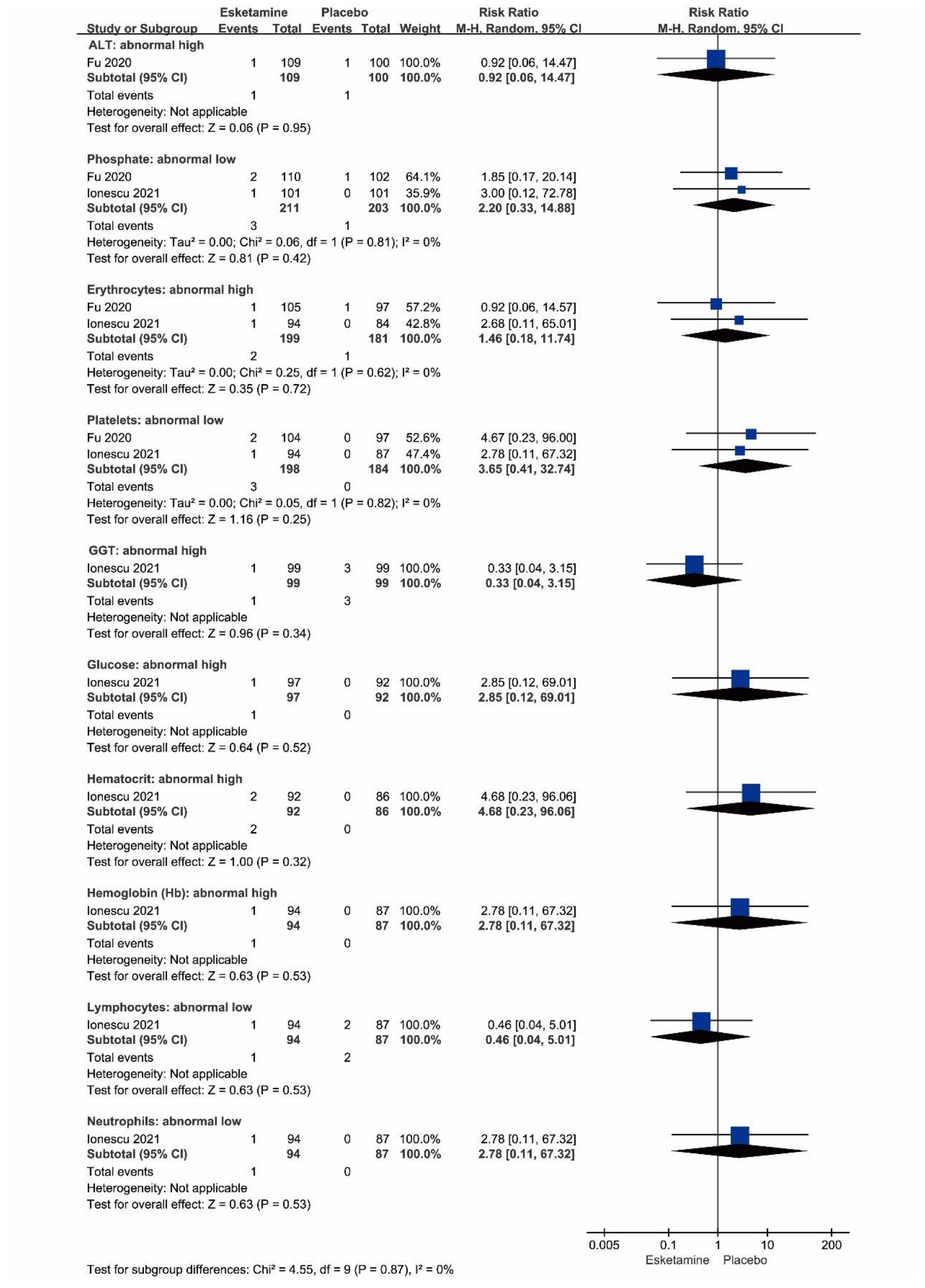

Supplementary Figure 16. Forest plot of the number of participants with treatment-emergent abnormal laboratory values after esketamine administration.

Abnormal laboratory values for variables are defined as ALT (abnormally high value = 200 U/L), phosphate (low = 0.7 mmol/L), erythrocytes (high = $6.4*10^{12}$/L), platelets (low = $100*10^{9}$/L), GGT (high = 300 U/L), glucose (high = 16.7 mmol/L), hematocrit (high = 0.55 fraction); Hb (high = 190 g/L), lymphocytes (low = 10%), and neutrophils (low = 30%). ALT, alanine aminotransferase; GGT, gamma glutamyl transferase; Hb, hemoglobin.

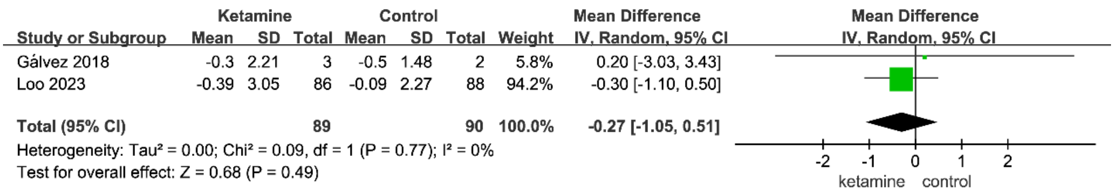

Supplementary Figure 17. Forest plot of changes in ketamine-related BPIC-SS scores from baseline to Week 4.

BPIC-SS, bladder pain-interstitial cystitis symptoms scale.

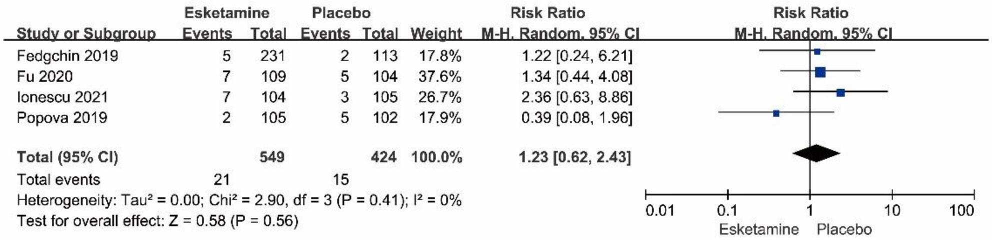

Supplementary Figure 18. Forest plot of the number of participants with nasal symptoms after esketamine administration.

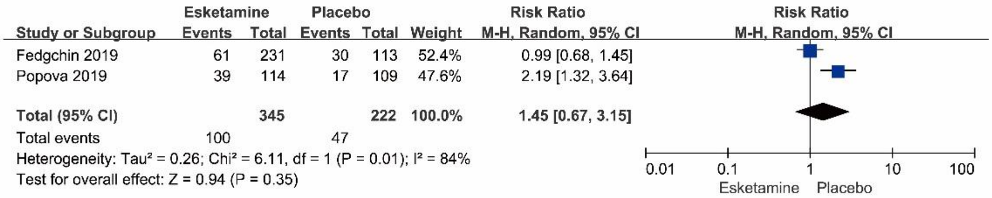

Supplementary Figure 19. Forest plot of the number of participants reporting moderate or severe nasal symptoms after esketamine administration.

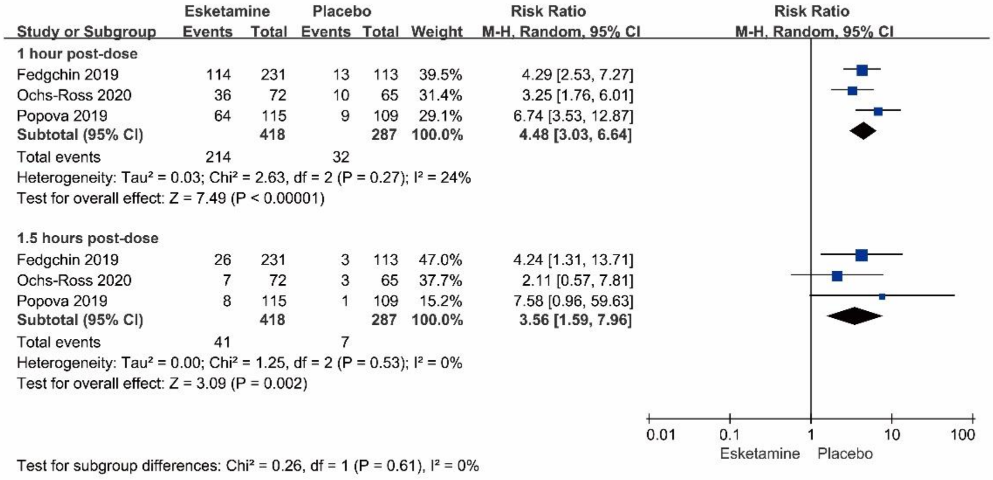

Supplementary Figure 20. Forest plot of the number of participants considered not ready for discharge after esketamine administration.

The outcomes are based on the CGADR scale. CGADR, clinical global assessment of discharge readiness.

Appendix 2. Search strategies (inception to 1 November, 2023)

Pubmed

#1 "depression"[mesh] OR "Mood Disorders"[mesh] OR "Affective Symptoms"[mesh] OR "Adjustment Disorders"[mesh]

#2 depress*[tiab] OR dysthymi*[tiab] OR "affective disorder*"[tiab] OR "affective symptom*"[tiab] OR "mood disorder*"[tiab] OR "adjustment disorder*"[tiab]

#3 #1 OR #2

#4 Ketamine[mesh] OR esketamine[mesh supplementary concept]

#5 ketamin*[tiab] OR ketalar[tiab] OR ketaject[tiab] OR ketanest[tiab]

#6 ketamin*[tiab] AND (enantiomer*[tiab] or "s-enantiomer*" [tiab])

#7 esketamin*[tiab] OR noresketamin*[tiab] OR "S-ketamin*" [tiab] OR Spravato[tiab]

#8 #4 OR #5 OR #6 OR #7

#9 (randomized controlled trial[pt] OR controlled clinical trial[pt] OR randomized[tiab] OR placebo[tiab] OR clinical trials as topic[mesh:noexp] OR randomly[tiab] OR trial[ti]) NOT (animals [mh] NOT (humans [mh] AND animals[mh]))

#10 #3 AND #8 AND #9

Cochrane Central Register of Controlled Trials (CENTRAL)

#1 MeSH descriptor: [Depression] explode all trees

#2 MeSH descriptor: [Mood Disorders] explode all trees

#3 MeSH descriptor: [Affective Symptoms] explode all trees

#4 MeSH descriptor: [Adjustment Disorders] explode all trees

#5 #1 OR #2 OR #3 OR #4

#6 (depress*):ti,ab,kw OR (dysthymi*):ti,ab,kw OR (affective disorder*):ti,ab,kw

#7 (affective symptom*):ti,ab,kw OR (mood disorder*):ti,ab,kw OR (adjustment disorder*):ti,ab,kw

#8 #6 OR #7

#9 #5 OR #8

#10 MeSH descriptor: [Ketamine] explode all trees

#11 (ketamin*):ti,ab,kw OR ("Ketalar"):ti,ab,kw OR (ketaject):ti,ab,kw OR (ketanest):ti,ab,kw

#12 (enantiomer*):ti,ab,kw OR ("s-enantiomer*"):ti,ab,kw

#13 (ketamin*):ti,ab,kw

#14 #12 AND #13

#15 (esketamin*):ti,ab,kw OR (noresketamin*):ti,ab,kw OR ("S-ketamin*"):ti,ab,kw OR (Spravato):ti,ab,kw

#16 #10 OR #11 OR #14 OR #15

#17 #9 AND #16

Embase

#1. 'depression'/exp

#2. 'mood disorder'/exp

#3. 'emotional disorder'/exp

#4. 'adjustment disorder'/exp

#5. #1 OR #2 OR #3 OR #4

#6. depress*:ab,ti
#7. dysthymi*:ab,ti
#8. 'affective disorder*':ab,ti
#9. 'affective symptom*':ab,ti
#10. 'mood disorder*':ab,ti
#11. 'adjustment disorder*':ab,ti
#12. #6 OR #7 OR #8 OR #9 OR #10 OR #11
#13. #5 OR #12
#14. 'ketamine'/exp
#15. 'esketamine'/exp
#16. #14 OR #15
#17. ketamin*:ab,ti
#18. ketalar:ab,ti
#19. ketaject:ab,ti
#20. ketanest:ab,ti
#21. #17 OR #18 OR #19 OR #20
#22. enantiomer*:ab,ti
#23. 's-enantiomer*':ab,ti
#24. #22 OR #23
#25. ketamin*:ab,ti
#26. #24 AND #25
#27. esketamin*:ab,ti
#28. noresketamin*:ab,ti
#29. 's-ketamin*':ab,ti
#30. spravato:ab,ti
#31. #27 OR #28 OR #29 OR #30
#32. #16 OR #21 OR #26 OR #31
#33. 'crossover procedure':de OR 'double-blind procedure':de OR 'randomized controlled trial':de OR 'single-blind procedure':de OR random*:de,ab,ti OR factorial*:de,ab,ti OR crossover*:de,ab,ti OR ((cross NEXT/1 over*):de,ab,ti) OR placebo*:de,ab,ti OR ((doubl* NEAR/1 blind*):de,ab,ti) OR ((singl* NEAR/1 blind*):de,ab,ti) OR assign*:de,ab,ti OR allocat*:de,ab,ti OR volunteer*:de,ab,ti
#34. #13 AND #32 AND #33

PsycINFO
#1 MA depression OR MA "Mood Disorders" OR MA “Affective Symptoms” OR MA “Adjustment Disorders”
#2 AB depress* OR AB dysthymi* OR AB “affective disorder*” OR AB “affective symptom*” OR AB “mood disorder*” OR AB “adjustment disorder*”
#3 #1 OR #2
#4 MA Ketamine OR MA esketamine
#5 AB ketamin* OR AB ketalar OR AB ketaject OR AB ketanest
#6 AB enantiomer* OR AB “s-enantiomer*”
#7 AB ketamin*

#8 #6 AND #7
#9 AB esketamin* OR AB noresketamin* OR AB “S-ketamin*” OR AB Spravato
#10 #4 OR #5 OR #8 OR #9
#11 TX SU.EXACT("Treatment Effectiveness Evaluation") OR SU.EXACT.EXPLODE("Treatment Outcomes") OR SU.EXACT("Placebo") OR SU.EXACT("Followup Studies") OR placebo* OR random* OR "comparative stud*" OR clinical NEAR/3 trial* OR research NEAR/3 design OR evaluat* NEAR/3 stud* OR prospectiv* NEAR/3 stud* OR (singl* OR doubl* OR trebl* OR tripl*) NEAR/3 (blind* OR mask*)

#12 #3 AND #10 AND #11
